# A Comprehensive LC–MS Metabolomics Assay for Quantitative Analysis of Serum and Plasma

**DOI:** 10.3390/metabo14110622

**Published:** 2024-11-14

**Authors:** Lun Zhang, Jiamin Zheng, Mathew Johnson, Rupasri Mandal, Meryl Cruz, Miriam Martínez-Huélamo, Cristina Andres-Lacueva, David S. Wishart

**Affiliations:** 1 Department of Biological Sciences, University of Alberta, Edmonton, AB T6G 2E8, Canada; lun2@ualberta.ca (L.Z.); jiamin3@ualberta.ca (J.Z.); mkj1@ualberta.ca (M.J.); rmandal@ualberta.ca (R.M.); 2 Biomarkers and Nutrimetabolomics Laboratory, Department of Nutrition, Food Sciences and Gastronomy, Nutrition and Food Safety Research Institute (INSA-UB), Faculty of Pharmacy and Food Sciences, University of Barcelona, 08028 Barcelona, Spain; mcruzb@ub.edu (M.C.); mmartinezh@ub.edu (M.M.-H.); candres@ub.edu (C.A.-L.); 3 Centro de Investigación Biomédica en Red de Fragilidad y Envejecimiento Saludable (CIBERFES), Instituto de Salud Carlos III, 28029 Madrid, Spain; 4 Department of Computing Science, University of Alberta, Edmonton, AB T6G 2E9, Canada; 5 Department of Laboratory Medicine and Pathology, University of Alberta, Edmonton, AB T6G 2B7, Canada; 6 Faculty of Pharmacy and Pharmaceutical Sciences, University of Alberta, Edmonton, AB T6G 2H7, Canada

**Keywords:** high-throughput, quantitative metabolomics, targeted metabolomics, LC–MS, plasma, serum

## Abstract

**Background/Objectives:** Targeted metabolomics is often criticized for the limited metabolite coverage that it offers. Indeed, most targeted assays developed or used by researchers measure fewer than 200 metabolites. In an effort to both expand the coverage and improve the accuracy of metabolite quantification in targeted metabolomics, we decided to develop a comprehensive liquid chromatography–tandem mass spectrometry (LC–MS/MS) assay that could quantitatively measure more than 700 metabolites in serum or plasma. **Methods:** The developed assay makes use of chemical derivatization followed by reverse phase LC–MS/MS and/or direct flow injection MS (DFI–MS) in both positive and negative ionization modes to separate metabolites. Multiple reaction monitoring (MRM), in combination with isotopic standards and multi-point calibration curves, is used to detect and absolutely quantify the targeted metabolites. The assay has been adapted to a 96-well plate format to enable automated, high-throughput sample analysis. **Results:** The assay (called MEGA) is able to detect and quantify 721 metabolites in serum/plasma, covering 20 metabolite classes and many commonly used clinical biomarkers. The limits of detection were determined to range from 1.4 nM to 10 mM, recovery rates were from 80% to 120%, and quantitative precision was within 20%. LC–MS/MS metabolite concentrations of the NIST^®^ SRM^®^1950 plasma standard were found to be within 15% of NMR quantified levels. The MEGA assay was further validated in a large dietary intervention study. **Conclusions:** The MEGA assay should make comprehensive quantitative metabolomics much more affordable, accessible, automatable, and applicable to large-scale clinical studies.

## 1. Introduction

There are two general ways of doing metabolomics: untargeted and targeted [1,2]. Untargeted metabolomics is a hypothesis-generating technique that is ideally suited for metabolite discovery. Specifically, untargeted methods attempt to measure all observable spectral features within a given biological sample. Once measured, spectral database queries are performed to tentatively identify the most significant spectral features and/or metabolites [3,4]. Given its potential for making exciting or unexpected compound discoveries, its low upfront cost, and its broad metabolite coverage (often >500 metabolites), untargeted metabolomics has become the most popular approach for performing metabolomics experiments. However, untargeted methods do have their drawbacks. In particular, untargeted metabolomics tends to be very labor intensive, poorly standardized, challenging to reproduce, non-quantitative, and less than ideal for high-throughput studies [5]. In contrast, targeted metabolomics is a hypothesis-driven approach that measures a limited number of predefined metabolites that are suspected of being biologically important [2]. In targeted methods, metabolites are identified and quantified by comparing acquired spectral data to authentic and carefully quantified standards placed directly into the sample or sample series. Targeted approaches can be absolutely quantitative. They are also highly reproducible, easily automated, and well-suited for high-throughput studies. Unfortunately, targeted metabolomics methods tend to have a high upfront cost (labor or material) and often provide much more limited metabolite coverage than untargeted approaches. As a result, targeted metabolomics studies are becoming much less popular.

This is an unfortunate trend. Indeed, for metabolomics discoveries to ultimately make their way into clinical, industrial, or environmental applications, the identified metabolites must be robustly identified and absolutely quantified. In fact, almost all the key discoveries in metabolomics involving biomarker discovery [6], medical diagnostics [7], drug development [8], and environmental testing [9] have employed targeted methods. While most targeted metabolomics studies typically measure <20 metabolites, there are a number of targeted assays that have been developed to identify and quantify many more metabolites. These include targeted nuclear magnetic resonance (NMR)-based methods that can be used to absolutely quantify up to 60 serum or plasma metabolites [10,11], 70 or more stool metabolites [12], and 40–70 wine compounds [13,14]. The most comprehensive targeted NMR assay so far described is a commercial one developed by Nightingale that measures 220 proteins, lipoproteins (sums/ratios), and small molecule metabolites [15]. As highlighted with the Nightingale assay, a key advantage of absolute quantification is that it allows the calculation of a large number of metabolite sums or ratios. This can greatly expand both the coverage and biological meaning of the measured metabolites in any targeted assay.

Targeted, fully quantitative LC–MS/MS methods have also been developed by several academic research groups. These assays can accurately quantify 142 compounds in urine [16], 721 compounds in serum/plasma (this assay), and 560 compounds in serum/plasma [17,18,19]. Additionally, there are a number of excellent commercial LC–MS assays or LC–MS kits that can measure and absolutely quantify between 180–1100 compounds [18,20]. For the most part, targeted metabolomics assays developed by academic researchers have been limited to measuring <200 metabolites, while targeted assays developed by commercial entities typically measure >200 metabolites. This discrepancy is likely because the resources and know-how to make “ultra” comprehensive targeted assays tend to lie exclusively with commercial entities. While commercial metabolomics kits and assays provide users with broad coverage, high standards, good reproducibility, excellent software, and strong support, they are also very expensive. Given the proclivity of metabolomics researchers to seek low-cost, open-access, DIY (do-it-yourself) solutions, this bias may explain why the use of targeted assays has been steadily falling.

In an effort to make targeted metabolomics more appealing to a broader metabolomics (and exposomics) audience, we decided to develop a comprehensive liquid chromatography–tandem mass spectrometry (LC–MS/MS) assay—called MEGA—that could quantitatively measure >700 metabolites in serum or plasma. Our primary goal in developing this MEGA assay was to learn about, and then share with the community, the methods, processes, and validation protocols needed to develop an “ultra” comprehensive, high-throughput, quantitative, targeted LC–MS/MS assay. Our secondary goal was to demonstrate that this assay could be successfully used in a clinical metabolomics/exposomics study.

The assay described here detects and quantifies 721 metabolites covering more than 20 different chemical classes. These include sixty-four amino acids and derivatives, fifty-three organic acids, nineteen biogenic amines, twenty-two nucleobases and nucleosides, four catecholamines, nine kynurenine-tryptophan pathway metabolites, seven ketone and keto acids, nine indole derivatives, three vitamins and derivatives, four sulfates, one dipeptide, two hundred forty-two triglycerides, seventy-five phosphatidylcholines, forty acylcarnitines, twenty-two cholesteryl esters, forty-four diglycerides, thirty-six ceramides, nineteen hexosylceramides, fourteen lysophosphatidylcholines, fourteen sphingomyelins, nine dihexosylceramides, six trihexosylceramides, two sugars, and three miscellaneous metabolites. To enable the high-throughput analysis and facile porting to other labs or platforms, the assay was specifically designed to be compatible with a 96-well plate format. Performance assessments (recovery, accuracy, precision, limits of detection, etc.) were conducted using pooled human serum samples. We also validated the quantitative performance of the MEGA assay by NMR spectroscopy of the well-known plasma metabolite standard NIST^®^ SRM^®^ 1950. To demonstrate its utility in metabolomics and/or exposomics studies, we used the MEGA assay to measure ~645 metabolites in 30 plasma samples obtained from a dietary intervention study aimed at treating mild cognitive impairment (MCI). Details regarding the MEGA assay design, required reagents, components, instrumentation, methods, performance specifications, assessment, and validation are provided in the following pages.

## 2. Materials and Methods

### 2.1. Chemicals, Reagents, and Materials

Optima™ LC/MS grade formic acid, Optima™ LC/MS grade water, methanol, and acetonitrile, Optima™ LC/MS grade ammonium acetate, MP Bio-medicals™ Serum (Human, Pooled) and phosphate-buffered saline were purchased from Fisher Scientific (Ottawa, ON, Canada). The 2,2-dimethyl-2-silapentane-5 sulfonate (DSS-d_6_), potassium phosphate monobasic, potassium phosphate dibasic, D_2_O (99.9%), HPLC grade water, pyridine, ethanol, and chloroform, phenylisothiocyanate (PITC), 3-nitrophenylhydrazines (3-NPH), 1-ethyl-3-(3-dimethylaminopropyl) carbodiimide (EDC), butylated hydroxytoluene (BHT), NIST^®^ SRM^®^ 1950, Nunc^®^ 96 DeepWell™ plates and Multiscreen “solvinert” filter plates (hydrophobic, PTFE, 0.45 μm, clear, non-sterile) were purchased from Sigma-Aldrich (Oakville, ON, Canada). The 2-chloropyrimidine-5-carboxylic acid (98%) was purchased from ArkPharm (Libertyville, IL, USA). The Amicon (1.5 mL) 3 kDa molecular weight cut-off (MWCO) filtration units were purchased from Millipore Sigma (St. Louis, MO, USA). The NMR tubes (3 mm) were purchased from Bruker Ltd. (Milton, ON, Canada). Chemical standards and isotope-labeled internal standards (ISTDs) were purchased from Sigma-Aldrich (Oakville, ON, Canada), Cayman Chemical (Ann Arbor, MI, USA), Toronto Research Chemicals (North York, ON, Canada), Cambridge Isotope Laboratories Inc. (Tewksbury, MA, USA), IsoSciences (Ambler, PA, USA), and C/D/N Isotopes Inc. (Pointe-Claire, QC, Canada), except 3-(3-hydroxyphenyl)-3-hydroxypropionic acid (HPHPA), which was synthesized in-house [21]. Detailed information on where each of these chemical standards and isotope-labeled ISTDs were purchased is provided in Supplementary Document S1.

### 2.2. Stock Solutions, Internal Standard (ISTD) Mixtures, and Calibration Curve Standards

Isotope-labeled ISTDs, along with isotope-labeled chemical derivatization reagents, were used for accurate metabolite quantification. All chemicals were weighed individually on a Sartorius CPA225D semi-micro electronic balance (Mississauga, ON, Canada) with a precision of 0.0001 g. Stock solutions, with defined concentrations for each analyte, were prepared by dissolving the accurately weighed chemicals in appropriate solvents. Seven different calibration curve standards (Cal1 to Cal7; detailed concentration levels are provided in Supplementary Table S1) were prepared by mixing and diluting corresponding stock solutions with appropriate solvents, covering different concentration ranges for different analytes according to their known or expected normal/pathological concentrations in human serum and plasma [22].

For amino acids, amino acid derivatives, biogenic amines, and nucleotides/nucleosides, double distilled water was used to prepare stock solutions, calibration mixtures, QC (quality control) mixtures, and a working ISTD solution mixture. The working ISTD solution mixture for these metabolites was made by combining 48 isotope-labeled ISTDs of defined concentrations from prepared stock solutions. Lipids stock solutions were prepared in chloroform. Acylcarnitine and hexose stock solutions were prepared in methanol. For lipids, acylcarnitines, and hexose, a working calibration mixture containing 21 standards of defined concentrations and a working ISTD solution mixture containing 18 ISTDs of specific concentrations in methanol were prepared by mixing all the prepared stock solutions. For organic acids, 75% (*v*/*v*) methanol in water was used to prepare stock solutions and calibration mixtures. An ISTD solution mixture was prepared in the same way as the calibration standards. A working ISTD solution mixture was prepared by derivatizing the ISTD solution using isotope-labeled chemical derivatization reagent during sample preparation (see Section 2.3.1 below). All the calibration solutions (concentrations described in Supplementary Table S1) used for the PITC panel were prepared in water, whereas those used for the 3-NPH panel were dissolved and further diluted using 75% aqueous methanol. All calibration solutions were stored in a −86 °C freezer prior to use.

### 2.3. Liquid Chromatography/Direct Flow Injection–Tandem Mass Spectrometry (LC/DFI–MS/MS) Analysis Using the MEGA Assay

#### 2.3.1. Sample Preparations

Serum and plasma samples were stored at −80 °C until used. Prior to their analysis, the samples were thawed on ice in the dark, vortexed thoroughly for 15 s, and then centrifuged at 13,000× *g* for 10 min. This assay uses 40 μL of serum or plasma for each sample (30 μL for organic acids analysis and 10 μL for lipids, acylcarnitines, and amine-containing compounds analysis) and a 96-well plate to facilitate automated high-throughput analysis. The first 14 wells of the 96-well plate are used for a double blank, three phosphate-buffered saline blank samples, seven calibration solutions, and three QC samples. Two separate sample preparation panels involving two different pre-column derivatization reactions were applied: (1) phenylisothiocyanate (PITC) derivatization panel and (2) 3-nitrophenylhydrazines (3-NPH) derivatization panel. No derivatization was required for analyzing lipids and acylcarnitines.

PITC derivatization was used to obtain the concentrations of amino acids, amino acid derivatives, biogenic amines, nucleotides/nucleosides, glucose/hexose, lipids, and acylcarnitines. This panel uses a 96-deep-well plate with a 96-well filter plate attached via sealing tape and a set of reagents and solvents used to prepare the plate assay. To 10 μL of each serum sample (or QC standards), 30 μL of the ISTD mixture solution (3 ISTD mixture solutions, each 10 μL) and 10 μL of various standards were pipetted directly onto the center of each corresponding spot/well in the upper filter plate. After drying the plate under a stream of nitrogen for 30 min, 50 μL of 5% PITC derivatization solution (where 300 μL of PITC reagent was added to a mixture of ethanol, water, and pyridine, each 1900 μL) was added to each well. The reaction was kept at room temperature for 20 min, followed by another 1.5 h of drying under a gentle nitrogen stream to remove the excess PITC solution. To extract the targeted analytes, 300 μL of methanol containing 5 mM ammonium acetate was then added to each spot. The whole plate was covered and shaken at 330 rpm using a Thermo Scientific 13687721 Thermal Mixer (Thermo Fisher Scientific, Waltham, MA, USA) for 30 min at room temperature and then centrifuged at 50× *g* for 5 min to collect the extracts from the upper filter plate to the bottom collection plate. Finally, 50 μL of extracts were transferred to a new 96-deep-well plate and then diluted with 450 μL of water for LC–MS/MS analysis to quantify amino acids, amino acid derivatives, biogenic amines, and nucleotides/nucleosides. A total of 10 μL of the remaining extracts were transferred to another 96-deep-well plate and then diluted with 490 μL of direct flow injection (DFI) buffer (which consisted of a mixture of 60 μL of formic acid, 10 mL of water, and 290 mL of methanol) for DFI–tandem mass spectrometry (DFI–MS/MS) analysis to quantify the lipids, acylcarnitines and glucose/hexose.

The 3-NPH derivatization method was used for the absolute quantification of organic acids. To precipitate the proteins, 90 µL of ice-cold methanol was mixed with 30 µL of each blank sample, calibration standard, QC standard, and serum sample in a 600 µL microfuge tube and then vortexed thoroughly for 30 s. Then, the samples were stored at −20 °C overnight to precipitate the proteins. The next morning, each sample was centrifuged at 13,000× *g* for 20 min at 4 °C. 50 µL of each supernatant was loaded to a selected well of a 96-deep-well plate. Then 75 µL of derivatization reagent (consisting of 25 µL of 250 mM 3-NPH in 50% aqueous methanol, 25 µL of 150 mM EDC in methanol, and 25 µL of 7.5% pyridine in 75% aqueous methanol) was added to each well. In a 1.5 mL microfuge tube, 125 µL of the working ISTD solution was prepared by mixing 50 µL of the ISTD mixture solution with 75 µL of isotope-labeled derivatization reagent (which consisted of 25 µL of 250 mM ^13^C_6_-3-NPH prepared in 50% aqueous methanol, 25 µL of 150 mM EDC prepared in methanol, and 25 µL of 7.5% pyridine prepared in 75% aqueous methanol). The 96-well plate was shaken at 500 rpm using a Thermo Scientific 13687721 Thermal Mixer for 2 h at room temperature. Then, 325 µL of water and 50 µL of BHT (2 mg/mL in methanol) were added to each well of the plate. The working ISTD solution, shaken under the same conditions, was diluted with 1125 µL of water, and 10 µL was then added to each well of a new 96-deep-well plate except for the double blank sample position. Next, 25 µL of the derivatized samples were transferred to the corresponding wells of the new plate. Finally, 215 µL of water was added to each well of the new plate before LC–MS/MS analysis.

#### 2.3.2. LC/DFI–MS/MS Analysis

The derivatized samples were delivered to the mass spectrometric via an Agilent 1260 series ultra-high performance liquid chromatography (UHPLC) system (Agilent Technologies, Palo Alto, CA, USA) equipped with an Agilent reversed-phase Zorbax Eclipse XDB C18 column (3.0 mm × 100 mm, 3.5 μm particle size, 80 Å pore size) with a Phenomenex (Torrance, CA, USA) SecurityGuard C18 guard column (4.0 mm × 3.0 mm). Mass spectrometric analysis was performed on an ABSciex 5500 QTrap^®^ tandem mass spectrometry instrument (Applied Biosystems/MDS Analytical Technologies, Foster City, CA, USA). Details of the UHPLC separation parameters for LC–MS/MS and DFI–MS/MS analyses of PITC-derivatized samples and LC–MS/MS analysis of 3-NPH-derivatized samples are given below. The controlling software for the sample analysis was Analyst 1.7.2 (Applied Biosystems/MDS Analytical Technologies, Foster City, CA, USA). Data analysis was performed using MultiQuantTM 3.0.3 (Applied Biosystems/MDS Analytical Technologies, Foster City, CA, USA).

For the UHPLC separation of the PITC panel for LC–MS/MS analysis and for DFI–MS/MS analysis, a gradient consisting of solvent A (0.2% (*v*/*v*) formic acid in water) and solvent B (0.2% (*v*/*v*) formic acid in acetonitrile), was run as follows: t = 0 min, 0% B; t = 0.5 min, 0% B; t = 5.5 min, 95% B; t = 6.5 min, 95% B; t = 7.0 min, 0% B; and t = 9.5 min, 0% B. The column oven temperature was set at 50 °C. The flow rate was 500 μL/min, and the sample injection volume was 10 μL. The mass spectrometer was set to a positive electrospray ionization mode with a scheduled multiple reaction monitoring (MRM) scan. The IonSpray voltage was set at 5500 V and the temperature at 500 °C. The curtain gas (CUR), ion source gas 1 (GAS1), ion source gas 2 (GAS2), and collision gas (CAD) were set at 20, 40, 50, and medium, respectively. The entrance potential (EP) was set to 10 V. The declustering potential (DP), collision energy (CE), collision cell exit potential (CXP), MRM precursor ion (Q1), and fragment ion (Q3) were optimized and set individually for each analyte and isotope-labeled ISTD.

For the UHPLC separation of the 3-NPH panel for LC–MS/MS analysis, targeting organic acids, ketone, and keto-acids, a different gradient, consisting of solvent A (0.01% (*v*/*v*) formic acid in water) and solvent B (0.01% (*v*/*v*) formic acid in acetonitrile) was run as follows: t = 0 min, 25% B; t = 6.0 min, 65% B; t = 6.3 min, 90% B; t = 6.5 min, 100% B; t = 7.0 min, 100% B; t = 7.5 min, 25% B; t = 12.0 min, 25% B. The column oven temperature was set to 40 °C. The flow rate was 400 μL/min, and the sample injection volume was 10 μL. The mass spectrometer was set to a negative electrospray ionization mode with scheduled MRM scanning. The IonSpray voltage was set at −4500 V and the temperature at 400 °C. The CUR, GAS1, GAS2, and CAD were set at 20, 30, 30 and medium, separately. The EP was set at −10 V, and the DP, CE, CXP, Q1, and Q3 were optimized and set individually for all the analytes and isotope-labeled ISTDs. Optimized MRM parameters, including Q1, Q3, DP, CE, and CXP, for all analytes are shown in Supplementary Table S2.

### 2.4. Method Validation

#### 2.4.1. Calibration Regression

For each of the analytes absolutely quantified via LC–MS/MS analysis, a seven-point calibration curve was generated using seven separate standard calibration solutions from which the calibration regression was assessed. The ratios of each analyte’s peak intensity to its corresponding ISTD were plotted against the known concentrations of each analyte to build an analyte-specific calibration curve, followed by calculation of the coefficient of determination (R^2^) and operator evaluation. Because of the widely ranging metabolite concentrations observed in healthy and diseased plasma/serum samples, an 80-fold variation (from the lowest to highest calibration concentrations) in calibrants was typically used. Therefore, a quadratic regression with 1/x weighting was used instead of a linear regression.

Due to the lack of individual chemical standards for each lipid and each acylcarnitine, these metabolites were analyzed semi-quantitatively via DFI–MS/MS. In these cases, a single point calibration of a representative analyte (one lipid representative for each lipid class and eight different acylcarnitines spanning different acyl chain lengths) was built to calculate the concentrations of compounds from the same group that share the same core structure, assuming linear regression through zero.

#### 2.4.2. Accuracy and Precision

To assess the accuracy and precision of the MEGA assay, three different concentration levels for each analyte (low, medium, and high) were prepared in triplicate using authentic standards. All these solutions were measured following the sample preparation method mentioned above, and the results were used to calculate (1) the accuracy by comparing the calculated concentrations with their fortified values and (2) the precision or coefficient of variation (CV) expressed as a percentage.

#### 2.4.3. Recovery

The MEGA assay was designed to work equally well for both serum and plasma samples. For the recovery tests, we chose to use serum. Specifically, assay recovery was evaluated by analyzing pooled human serum along with the serum samples that had been spiked at the three different concentrations (low, medium, and high). All spiked samples and non-spiked pooled human serum samples were prepared in triplicate. The recovery was expressed as a percentage and was calculated according to Equation (1).

Recovery (%) = (C_SP_ − C_NS_)/C_A_ × 100 (1)

C_SP_ is the calculated concentration in the spiked sample, C_NS_ is the calculated concentration in the non-spiked sample, and C_A_ is the theoretical concentration of the analyte that was spiked into the pooled serum sample.

#### 2.4.4. Limits of Detection and Quantification

The limit of detection (LOD) and limit of quantification (LOQ) were calculated using blank sample determination. This was performed by analyzing three replicates of the blank samples where a non-zero standard deviation was obtained. LODs and LOQs were calculated using Equations (2) and (3), where C_B_ is the average concentration of the blank, and SD_B_ is the standard deviation of the blank.

LOD = C_B_ + 3 × SD_B_ (2)

LOQ = C_B_ + 10 × SD_B_ (3)

However, for any analyte with zero standard deviation, its LOD and LOQ were estimated using a signal-to-noise (S/N) method. The peak-to-noise ratio was measured at a known concentration level using the Sciex Analyst^®^ 1.7.2 software, and the analyte concentrations yielding an S/N ratio equal to 3 or 10 were estimated as the LOD or LOQ, respectively.

### 2.5. High-Resolution NMR Analysis of NIST^®^ SRM^®^ 1950

Plasma and serum samples contain a significant amount of high molecular weight proteins and lipoproteins, which affect the identification of low molecular weight metabolites by NMR spectroscopy. Ultra-filtration was used to remove plasma proteins in NIST^®^ SRM^®^ 1950 (as previously described [23]). To perform the ultrafiltration step, 3 kDa MWCO ultrafiltration units were first rinsed five times each with 0.5 mL of H_2_O and centrifuged 10,000× *g* for 10 min to remove any residual glycerol bound to the filter membranes. After the washing step was completed, 400 µL of the SRM 1950 plasma sample was transferred into the centrifuge filter devices and spun (10,000× *g* for 20 min at 4 °C). To 200 µL of the plasma sample, 50 µL of a standard buffer solution (54% D_2_O:46% H_2_O containing 1.75 mM KH_2_PO_4_ pH 7.0 *v*/*v* containing 5.84 mM DSS [2,2-dimethyl-2-silopentane-5-sulphonate], 5.84 mM 2-chloropyrimidine-5 carboxylate) was added. The plasma sample (250 µL) was then transferred to a 3 mm SampleJet NMR tube for the subsequent spectral analysis.

All ^1^H-NMR spectra were collected on a 700 MHz Avance III (Bruker Biospin, Rheinstetten, Germany) NMR spectrometer equipped with a 5 mm HCN Z-gradient pulsed-field gradient (PFG) cryoprobe. ^1^H-NMR spectra were acquired at 25 °C. The number of data points was fixed to 128 K, and the spectral width was set to 12 ppm. A total of 128 transients were collected for each spectrum with a 3-second recycle delay between transients. ^1^H-NMR spectra were acquired at 25 °C using the first transient of the NOESY pre-saturation pulse sequence (noesy1dpr), chosen for its high degree of quantitative accuracy [24]. All FIDs (free induction decays) were zero-filled to 250 K data points. The singlet produced by the DSS methyl groups was used as an internal standard for chemical shift referencing (set to 0 ppm) and for quantification. All ^1^H-NMR spectra were processed and analyzed using an in-house version of the MagMet automated NMR analysis software package using a custom serum metabolite library [10]. Notably, since the cryoprobe is particularly sensitive to the amount of salt in a sample, using a smaller 3 mm SampleJet NMR tube with a 5 mm cryoprobe reduces the total salt seen by the probe and improves the signal-to-noise ratios in the NMR spectra [25].

### 2.6. Analysis of Plasma Samples Acquired During a Dietary Intervention Study of Mild Cognitive Impairment (MCI)

#### 2.6.1. Dietary Intervention Plasma Samples

To further validate the suitability of the MEGA assay for clinical studies, a set of 30 plasma samples was tested. These samples were obtained from a randomized, crossover, double-blind, trial-controlled study (clinicalTrials.gov ID NCT05029765) that was conducted at the Maimónides Biomedical Research Institute of Cordoba (IMIBIC, for its initials in Spanish) and the Reina Sofía University Hospital in Spain [26]. From 189 participants, 47 mild cognitive impairment (MCI) patients (≥60 y, 57.4% males) were selected and randomized to receive the following three dietary interventions: (1) a Mediterranean diet supplemented with probiotics (109 colony-forming units of *Lactobacillus rhamnosus* and *Bifidobacterium longum*); (2) a Mediterranean diet; and (3) a healthy diet according to the World Health Organization (WHO) recommendations [26]. All plasma samples collected from this study were stored at −80 °C. To assess the MEGA assay, frozen plasma samples processed from 15 participants receiving the Mediterranean diet just before (at baseline, V1) and immediately after (at 24 weeks, V2) the dietary intervention were collected, processed, and measured via the MEGA assay. Details of the participant selection, inclusion and exclusion criteria, and dietary intervention protocols are found in Cardelo et al., 2022 [26].

#### 2.6.2. Data Analysis

The results from the Mediterranean diet dietary intervention at time 0 (baseline, V1) were compared to day 24 (V2) (*n* = 30 paired samples, 15 V1 (baseline) + 15 V2 (timepoint 2)). The quantified data, expressed in micromolar units, were then processed using the R statistical package (version 4.4.1) [27]. Metabolites with >20% missing values (<LOD) in any given sample were filtered and removed. The remaining missing values were imputed to half of the minimum LOD value. A Student’s *t*-test analysis was applied after data normalization using log transformation and Pareto scaling to look for differentially expressed metabolites before and after the diet intervention. Percent changes were calculated using the Equation (4) below:

Percent Change (%) = (post intervention concentration − baseline concentration)/(baseline concentration) × 100(4)

## 3. Results and Discussion

### 3.1. Liquid Chromatography

**Figure 1:**
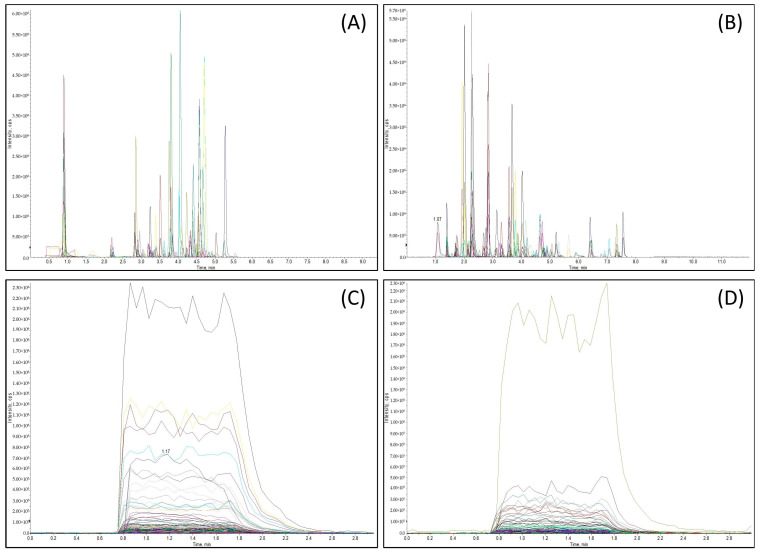
Overlaid extracted ion chromatograms (EICs) of extracted metabolites and ISTDs in human serum. (**A**) EICs of amino acids, biogenic amines, and their derivatives, as well as other positively charged metabolites. (**B**) EICs of organic acids, ketone, and keto-acids, as well as other negatively charged metabolites. (**C**) EICs of acylcarnitines, hexose, phospholipids, and sphingomyelins. (**D**) EICs of cholesteryl esters, ceramides, diglycerides, and triglycerides.

Figure 1, showing the overlaid representative extracted ion chromatograms (EICs) of the pooled human serum after sample preparation, shows a high level of analyte multiplexing (up to 741 metabolites). Three separate injections were involved in the PITC panel: one HPLC injection had a 9.5 min running time (per sample) and was used to quantify amino acids and biogenic amines, while the other two injections had 3 min running times, each via DFI–MS/MS, targeting different classes of metabolites, including lipids, acylcarnitines, and hexoses. The single injection corresponding to the 3-NPH panel had a running time of 12 min per sample. Some isomer-co-elutions were observed in both the PITC and 3-NPH panels. Co-eluted isomers with distinct Q3 fragment ions, such as leucine and isoleucine, were separated using different MRM transitions (Table S2). Almost all isomers with the same MRM transitions, such as 2-hydroxyphenylacetic acid, 3-hydroxyphenylacetic acid, and 4-hydroxyphenylacetic acid, eluted from the column at different retention times and could be well separated. The exceptions were butyric acid and isobutyric acid (no LC or MRM separation was possible). As a result, the total concentration of the two acids was calculated and reported. All the lipids, acylcarnitines, and hexose analyzed via DFI–MS/MS co-eluted together but were easily separated by their distinct MRM transitions.

### 3.2. Assay Validation

Absolute quantification of both LC–MS/MS analyses was performed using the peak area ratios of the targeted analyte compared to its corresponding isotopically labeled ISTD. A specific calibration curve was built for each of the analytes within its concentration range (see Table S1 for calibrants C1–C7). The expected concentrations in normal human serum samples were obtained from the Human Metabolome Database (HMDB, [22]). No significant carry-over was observed by comparing the highest concentration calibrant (Cal 7) to the following double-blank injection. The LOD, the LOQ, and the calibration curve R^2^ of each analyte are listed in Supplementary Table S3, with a number of representative metabolites summarized in Table 1. Note that LODs range from the nanomolar (0.0131 µM or 13.1 nM) for N-acetyl-proline to the micromolar (2.0 µM) for lactic acid. Intra-day and inter-day accuracy and precision were assessed by analyzing the low, medium, and high concentration levels of QC samples on three different days. All the calculated results are summarized in Supplementary Table S4, from which a small set of typical results were selected and shown in Table 2. Some of the highest differences in accuracies were seen when samples were prepared with the lowest analyte concentrations (see 0.8 µM epinephrine, which had 114% intra-day and 111% inter-day variation, and 0.5 µM N-acetyl-proline, which had 90.4% intra-day and 91.4% inter-day variation). However, with a target accuracy criterion between 80–120% and precision within 20%, our results show that the LC–MS/MS analyses of the PITC and 3-NPH panels met the criteria for accuracy and reproducibility.

To assess the losses during sample preparation, spiking experiments at three different concentration levels (low, medium, and high) into commercial pooled human serum were performed. Recoveries of absolutely quantified metabolites were assessed by comparing the calculated spiked concentrations with the theoretically spiked concentrations. A selection of representative analyte recoveries is listed in Table 3, and the complete summary of the recovery performance of all analytes is provided in Supplementary Table S5. Recoveries for some metabolites (i.e., creatinine and valine) were greater than the spiked concentrations, while others (i.e., epinephrine and 2-hydroxybutyric acid) were lower than the spiked concentrations. Decreased or increased recoveries did not correspond with the decreased or increased spiked concentrations but appeared to be metabolite specific. No correction factor was applied, as these calibration curves can accurately quantify metabolites in serum.

Because acquiring authentic chemical standards for each of the analytes analyzed via the DFI–MS/MS steps is impossible, semi-quantification using single-point calibration was performed. The LODs and LOQs of each analyte were obtained using the blank determination method. Reproducibility was evaluated by analyzing pooled human serum in triplicate. Accuracy, precision, and recoveries were assessed only for those analytes with authentic standards used in this assay. All the results are summarized in Supplementary Table S6, with a subset shown in Table 4. Those metabolites with the highest differences in accuracy and precision and the lowest recoveries were the diglyceride or DG(18:1/18:1) and the triglyceride or TG(18:1/36:2). However, all values were within formally accepted criteria of accuracy being between 80–120% and precision within 20%.

### 3.3. Orthogonal Validation on NIST^®^ SRM^®^ 1950 by NMR

To further validate the MEGA assay performance, the NIST^®^ SRM^®^ 1950 plasma sample was analyzed by both the LC–MS/MS MEGA assay and by ^1^H NMR spectroscopy. The NIST^®^ SRM^®^ 1950 plasma was prepared from blood collected into lithium heparin tubes from 100 adult donors [28]. Metabolites were identified and quantified by analyzing the 1D-NOESY ^1^H NMR spectrum of the NIST^®^ SRM^®^ 1950 using an in-house developed software package called MagMet [10]. The concentrations of the metabolites that could be analyzed accurately by both platforms were compared. As shown in Table 5, all the 34 metabolites that were quantified in NIST^®^ SRM^®^ 1950 by both LC–MS/MS and NMR have deviations within 15% of each other. These results demonstrate the good agreement when the MEGA assay is compared to NMR quantification. The results obtained from the MEGA assay also match the expected concentrations of 18 metabolites listed in the NIST^®^ SRM^®^ 1950 certificate of analysis (COA). This demonstrates that the MEGA assay can accurately quantify metabolites in this well-established reference standard. The fact that the MEGA assay was originally developed, tested, and validated on serum but the NIST assessment was performed on plasma clearly demonstrates that the MEGA assay works well with both biofluids. While metabolite concentrations in plasma and serum can differ [29], they are all well within the ranges created for this MEGA assay.

### 3.4. Application to a Dietary Intervention Study

To demonstrate the utility of the MEGA assay in metabolomics and/or exposomics studies, we used it to measure plasma samples acquired from a recently published dietary intervention study. The study consisted of a controlled, crossover, double-blind trial (clinicalTrials.gov ID NCT05029765) where 15 mild cognitive impairment (MCI) patients (≥60 y, 57.4% males) were randomized to consume a Mediterranean diet for 24 weeks [26]. Stored plasma samples from the participants receiving the Mediterranean diet at baseline (V1) and after 24 days of the dietary intervention (V2) were analyzed by the MEGA assay (*n* = 30 paired samples, 15 V1 (baseline) + 15 V2 (timepoint 2)). The mean concentration and standard deviations for all quantified metabolites for the two time points are listed in Supplementary Table S7. Globally, 457 (70.9%) of the 645 total metabolites from all four methods were successfully quantified in the 30 paired samples analyzed. Of the 457 metabolites analyzed, 89 metabolites increased, and 368 metabolites decreased after the intervention. Several metabolites (at baseline) were detected in high concentrations, including cholesteryl esters, ceramides, triglycerides, and phosphatidylcholines, which may be indicative of metabolic changes associated with MCI [30]. These metabolites could also indicate other physiological effects associated with higher dementia risk, such as cardiovascular and metabolic disorders [31].

To determine whether the changes in metabolite levels after the intervention were statistically significant, *t*-tests were performed. The results (Table 6) revealed 21 statistically significant (*p*-value < 0.05) metabolic changes after the Mediterranean dietary intervention, with 20/21 metabolites decreasing and 1/21 metabolites increasing. Among the compounds that showed significant decreases in plasma concentrations after the diet were beta-alanine, ceramides, glucosylceramides, triglycerides, and phosphatidylcholines. Given that the Mediterranean diet is known to improve cognitive performance in MCI and AD patients [32,33], decreased plasma concentrations of these metabolites could serve as biomarkers of improved cognitive performance.

Overall, we found that the MEGA assay could be successfully used in a clinically-based dietary intervention study and that 645 metabolites could be routinely detected and quantified in human plasma via this assay. The metabolite coverage obtained and the accuracy of the metabolite quantifications by the MEGA assay were such that significant changes in many endogenous (and diet-related) metabolites could be detected after the dietary intervention. It is important to emphasize that the results and interpretation of this dietary intervention are not the central objective of this manuscript and that a much more comprehensive assessment of the dietary effects will be the subject of a different manuscript. Nevertheless, these results highlight the broad applicability of the MEGA assay for studying the metabolic effects of dietary interventions and potentially other clinical or nutritional studies.

### 3.5. Method Comparison

We have compared our comprehensive assay with other published targeted, quantitative assays for analyzing serum/plasma samples. Most of the published methods only target a small set of metabolites, such as bile acids [34] or steroids [35]. Only a small number of published or commercial targeted MS assays offer comprehensive, quantitative metabolite measurements [17,18,19]. However, they either provide much lower levels of metabolite coverage [17], use fewer calibration standards and/or isotope-labeled ISTDs [18,19], are not calibrated to the physiological concentration ranges expected in serum/plasma [19], or do not present full validation details [19]. As far as we are aware, our MEGA assay is the most comprehensive LC–MS/MS quantitative method developed by an academic laboratory to date.

### 3.6. Platform Compatibility

The MEGA assay, as described here, is specific to a Sciex QTrap 5500 instrument equipped with an Agilent UHPLC system and the Sciex software (Analyst 1.7.2). Over the past few months, we (and others) have successfully adapted the MEGA assay to work on several other LC–MS platforms, including Sciex QTraps 6500 and 7500, Thermo Altis (QQQ), Thermo Q-Exactive Orbitrap, Agilent 6495D and Waters Xevo, equipped with Agilent, Waters, Exion, or Shimadzu HPLC/UHPLC systems. As might be expected, platform-specific adaptations were necessary to fully implement the assay, but in most cases, the porting to each platform could be performed in less than a week. In most cases, either the instrument-specific software (Analyst for Sciex, MassHunter for Agilent, TraceFinder for Thermo, MassLynx for Waters) or publicly accessible software [36] was used to perform the data reduction and analysis. These results suggest that the MEGA assay design is sufficiently robust and general to be ported to a wide range of instruments and manufacturers.

## 4. Conclusions

We have reported a comprehensive and quantitative LC–MS/MS assay for the detection and quantification of up to 721 serum/plasma metabolites, covering more than 20 different chemical classes. The assay has been specifically designed to work using a 96-well format so as to support high-throughput automation. The assay is highly sensitive and is able to work with as little as 40 μL of serum/plasma. To simplify the HPLC separation process (it only needs one reverse phase column), increase the ionization efficiency, and reduce the costs of purchasing ISTDs, the assay uses chemical derivatization to “tag” a significant number of metabolites. Specifically, PITC derivatization is used to label primary and secondary amines, while 3-NPH is used to label carboxylic acids, ketone, and keto-acids. Details regarding how the assay was designed, the reagents and plasticware used and the protocols for constructing, running, and calibrating the assay have been provided. This information is intended to help inform and educate the metabolomics community so that the MEGA assay or similar assays can be developed and/or run in-house. Additionally, details regarding the MEGA assay validation process, as well as the results from the calibration, LOD, LOQ, accuracy, precision, and recovery measurements at three different concentration levels, have been provided. Quantitative NMR spectroscopy was also used to validate the MEGA-measured concentrations of a number of metabolites in the NIST^®^ SRM^®^ 1950 sample. The results showed excellent agreement between the two methods. To demonstrate the MEGA assay’s utility in clinical metabolomics and/or exposomics studies with real samples, we showed how it could be successfully applied to multiple samples collected from a dietary intervention study and that it could yield interesting and meaningful results. Comparisons of the MEGA assay to other targeted MS assays, in terms of performance, coverage, and features, indicate that the MEGA appears to be the most comprehensive and robust targeted assay so far described by any academic laboratory. The MEGA assay has been successfully ported to a number of other Qtrap, QQQ, and Orbitrap platforms, indicating its robustness and facile compatibility with different instrumentation. It is also notable that since 2023, more than 3000 serum/plasma samples have been analyzed in our laboratory using the MEGA assay. Given its accuracy, precision, robustness, sensitivity, and broad metabolite coverage, we believe the MEGA assay should make targeted, quantitative metabolomics much more appealing to a much wider community.

## Figures and Tables

**Table 1:** Calibration regression, LOD, and LOQ of a subset of analytes measured in the MEGA assay.

Analyte	Correlation Coefficient (R^2^)	LOD (μM)	LOQ (μM)
Creatinine Tryptophan	0.9993	0.327	1.09
	0.9998	0.0340	0.113
Epinephrine Valine	0.9997	0.0100	0.0334
	0.9989	0.295	0.982
2-Hydroxybutyric acid Succinic acid Lactic acid N-Acetyl-proline	0.9993	0.115	0.384
	0.9997	0.167	0.556
	0.9992	2.00	6.66
	0.9982	0.0131	0.0437

**Table 2:** Intra- and inter-day accuracy and precision from a representative set of analytes from the MEGA assay.

Analyte		Intra-day		Inter-day	
	Fortified concentration (μM)	Accuracy (%)	CV (%)	Accuracy (%)	CV (%)
Creatinine	80.0	104	2.26	106	3.72
	320	106	4.02	111	5.72
	640	105	0.223	105	4.60
Tryptophan	40.0	102	2.43	99.8	3.07
	160	102	1.49	101	4.51
	320	111	4.79	104	6.83
Epinephrine	0.800	114	1.00	111	2.39
	3.20	105	0.983	104	7.21
	6.40	94.0	2.48	96.4	9.82
Valine	80.0	97.5	2.53	100	2.54
	320	99.7	1.60	101	2.76
	640	113	3.92	108	4.53
2-Hydroxybutyric acid	16.0	99.1	1.98	96.9	2.34
	64.0	97.4	2.74	94.8	6.02
	128	103	1.24	101	4.75
Succinic acid	8.00	99.0	1.13	102	2.74
	32.0	97.8	0.332	97.5	4.77
	64.0	104	1.43	106	1.98
Lactic acid	800	104	3.23	100	4.78
	3200	98.9	3.87	95.2	4.11
	6400	106	1.68	103	2.18
N-Acetyl-proline	0.500	90.4	1.06	91.4	3.10
	2.00	91.7	1.28	90.6	4.79
	4.00	97.9	1.64	99.5	1.88

**Table 3:** Recovery performance of a representative set of analytes in spiked pooled human serum.

Analyte	Spiked Concentration (μM)	Calculated Concentration (μM)	Recovery (%)
Creatinine	10.0	10.9	109
	100	110	110
	300	336	112
Tryptophan	5.00	5.30	106
	50.0	52.5	105
	150	153	102
Epinephrine	0.100	0.0836	83.6
	1.00	0.864	86.4
	3.00	2.50	83.4
Valine	10.0	11.8	118
	100	107	107
	300	321	107
2-Hydroxybutyric acid	2.00	1.79	89.6
	20.0	18.6	93.1
	60.0	53.5	89.1
Succinic acid	1.00	0.934	93.4
	10.0	9.63	96.3
	30.0	27.3	90.9
Lactic acid	100	92.9	92.9
	1000	954	95.4
	3000	2757	91.9
N-Acetyl-proline	0.0625	0.0603	96.5
	0.625	0.579	92.6
	1.88	1.84	98.0

**Table 4:** Validation performance of a subset of selected analytes analyzed via DFI-MS/MS.

Analyte	Accuracy (%)			CV (%)			Recovery (%)			LOD (μM)
	Low	Mid	High	Low	Mid	High	Low	Mid	High	
Carnitine (C0)	101	104	90.6	13.0	7.82	1.25	97.3	109	106	0.222
Hexose	99.1	100	101	1.88	0.961	3.92	103	97.2	105	22.5
CE(17:0)	98.9	97.0	98.3	6.91	9.79	6.76	110	108	94.5	0.126
Cer(d18:1/18:0)	112	114	120	3.23	2.20	4.51	96.3	91.2	97.2	0.0104
DG(18:1/18:1)	111	111	112	4.99	4.47	5.63	88.2	89.1	87.3	0.0356
TG(18:1/36:2)	98.7	94.7	110	4.08	7.45	10.8	89.3	86.3	92.4	0.0334
LacCer(d18:1/18:0)	105	106	110	4.14	3.60	1.61	109	116	102	0.0104
LysoPC a C18:0	99.3	93.9	93.9	3.34	2.55	2.99	92.5	98.3	91.2	0.0532
SM C18:0	105	97.4	101	3.39	5.00	3.08	109	110	94.2	0.127
PC aa C36:0	101	104	105	4.50	1.63	2.34	92.3	107	96.2	0.159

Abbreviations: C—carnitine; CE—cholesterol ester; Cer—ceramide; DG—diglyceride, LacCer—lactosylceramides; lysoPC—lysophosphatidylcholines; PC aa—phosphatidylcholine diacyl (acyl-acyl); SM—sphingomyelin; TG—triglyceride.

**Table 5:** Comparison of analyte concentrations measured by both LC–MS/MS and NMR for NIST^®^ SRM^®^ 1950.

Analyte	MEGA Assay (Average ± SD, µM)	NMR (µM)	NIST^®^ SRM^®^ 1950 COA (µM)
3-Hydroxybutyric acid	128 ± 3.90	139	/
Acetylcarnitine	7.20 ± 0.261	8.3	/
Alanine	303 ± 14.5	299	300 ± 26.0
alpha-Aminobutyric acid	13.3 ± 1.42	12.4	/
Arginine	83.8 ± 3.45	77.1	81.4 ± 2.30
Asparagine	31.2 ± 1.82	27.3	/
Aspartic acid	8.55 ± 0.537	8.16	/
Betaine	45.9 ± 3.58	46.0	/
Carnitine	31.6 ± 2.22	32.5	/
Choline	14.6 ± 0.938	12.9	/
Citrulline	32.8 ± 3.51	/	/
Creatinine	67.4 ± 1.43	58.8	60.0 ± 0.900
Hexose/Glucose	4469 ± 101	4464.8	4560 ± 56.0
Glutamic acid	62.5 ± 4.88	53.8	/
Glutamine	464 ± 12.3	428	/
Glycine	242 ± 7.11	248	245 ± 16.0
Histidine	68.8 ± 3.42	65.8	72.6 ± 3.60
Hypoxanthine	3.10 ± 0.143	2.83	/
Isoleucine	55.1 ± 5.03	52.4	55.5 ± 3.40
Lactic acid	2370 ± 108	2538	/
Leucine	94.5 ± 7.41	103	100 ± 6.30
Lysine	146 ± 3.19	148	140 ± 14.0
Methionine	22.1 ± 1.18	20.4	22.3 ± 1.80
Ornithine	59.3 ± 3.09	56.0	/
Phenylalanine	53.7 ± 3.02	51.6	51.0 ± 7.00
Proline	188 ± 5.82	178	177 ± 9.00
Pyruvic acid	65.9 ± 4.88	72.8	/
Serine	98.2 ± 3.77	86.1	95.9 ± 4.30
Succinic acid	2.23 ± 0.154	2.1	/
Threonine	118 ± 4.73	117	120 ± 6.10
Tyrosine	55.1 ± 3.53	57.1	57.3 ± 3.00
Urea	3780 ± 222	/	3900 ± 80.0
Uric acid	244 ± 5.66	/	254 ± 5.00
Valine	183 ± 2.33	178	182 ± 10.4

**Table 6:** Differential metabolites identified in plasma samples (*t*-test, *p* < 0.05) from mild cognitive impairment patients before (V1) and after (V2) a 24-week Mediterranean dietary intervention.

Metabolite	*p*-Value	Percent Change (%)	V1 Mean ± SD (µM)	V2 Mean ± SD (µM)
Cer(d18:2/22:0)	0.0120	−15.9	0.150 ± 0.0727	0.126 ± 0.0631
TG(18:3_32:0)	0.0122	−28.4	0.830 ± 0.660	0.594 ± 0.544
Cer(d18:2/24:0)	0.0130	−16.0	0.416 ± 0.198	0.350 ± 0.163
HexCer(d18:2/20:0)	0.0137	−28.3	0.0383 ± 0.0223	0.0275 ± 0.0194
TG(16:0_34:4)	0.0243	−18.3	0.656 ± 0.526	0.536 ± 0.483
beta-Alanine	0.0259	−12.5	1.57 ± 0.431	1.37 ± 0.449
HexCer(d18:2/22:0)	0.0285	−13.9	0.171 ± 0.104	0.147 ± 0.0809
TG(20:4_34:3)	0.0303	−24.6	0.633 ± 0.496	0.477 ± 0.395
TG(20:4_32:0)	0.0307	−34.9	0.881 ± 0.921	0.573 ± 0.692
PC aa C36:4	0.0317	−12.9	202 ± 102	176 ± 60.1
TG(20:3_34:3)	0.0351	−18.4	0.331 ± 0.280	0.270 ± 0.239
PC ae C36:4	0.0396	−11.5	15.0 ± 5.04	13.3 ± 4.11
LysoPC a C20:4	0.0401	−20.3	4.50 ± 3.48	3.59 ± 1.71
PC aa C34:4	0.0409	−23.7	1.36 ± 0.710	1.03 ± 0.475
PC aa C32:1	0.0446	−15.2	19.8 ± 8.15	16.8 ± 5.44
TG(20:4_32:1)	0.0452	−20.9	1.02 ± 0.834	0.810 ± 1.03
TG(20:4_34:2)	0.0459	−20.9	4.11 ± 3.79	3.25 ± 3.05
PC aa C38:4	0.0471	−9.25	11.4 ± 4.19	9.72 ± 3.80
PC aa C34:3	0.0482	−14.4	11.4 ± 4.19	9.72 ± 3.80
TG(16:1_38:5)	0.0493	−17.2	0.504 ± 0.347	0.418 ± 0.292
TG(18:1_36:2)	0.0494	22.4	96.7 ± 64.0	118 ± 52.8

Abbreviations: Cer—ceramide; HexCer—hexocylceramide; lysoPC—lysophosphatidylcholine; PC aa—phosphatidylcholine diacyl (acyl-acyl); PC ae—phosphatidylcholine acyl-alkyl; TG—triglyceride.

## Data Availability

Data from the MEGA assay analysis is available upon request. However, data obtained from the dietary intervention study will not be made available to protect patient confidentiality.

## Supplementary Materials

The following materials are available online at https://www.mdpi.com/article/10.3390/metabo14110622/s1. Supplementary Document S1: Detailed Chemical Vendor Information, Supplementary Table S1: Concentration of Standards at Different Calibration Levels; Table S2: Optimized MRM Transitions and Mass Spectrometer Parameters; Table S3: Calibration Regression, LOD, and LOQ; Table S4: Intra- and Inter-day Accuracy and Precision; Table S5: Recovery Performance in Spiked Pooled Human Serum; Table S6: Validation Performance of Analytes Analyzed via DFI–MS/MS; and Table S7: Mean Concentrations and Standard Deviations of Detected Metabolites for the Two Time Points in the Intervention Study.
