# Supplementary material for: A Comprehensive LC–MS Metabolomics Assay for Quantitative Analysis of Serum and Plasma
Source: Metabolites. 2024 Nov 14;14(11):622. doi: 10.3390/metabo14110622 (PMC11596266; doi:10.3390/metabo14110622)
Supplement: Supplementary file 1 [file metabolites-14-00622-s001.zip › Supplementary Information.pdf]

Supplementary Materials

Contents

Supplementary Document S1 1

Supplementary Table S1. Concentration of Standards at Different Calibration Levels 4

Supplementary Table S2. Optimized MRM Transitions and Mass Spectrometer Parameters 11

Supplementary Table S3. Calibration Regression, LOD and LOQ 35

Supplementary Table S4. Intra- and Inter-day Accuracy and Precision 42

Supplementary Table S5. Recovery Performance in Spiked Pooled Human Serum 49

Supplementary Table S6. Validation Performance of Analytes Analyzed *via* DFI-MS/MS 56

Supplementary Table S7. Mean Concentrations and Standard Deviations of Detected Metabolites for the Two Time Points in the Intervention Study 74

Supplementary Document S1.

Detailed chemical vendor information

Chemical standards including 1,3-diaminopropane, 1-methylnicotinamide, alpha-aminoadipic acid, 3-methoxytyramine, 3-nitrotyrosine, 5-methoxytryptamine, 5-methyluridine, 7-methylguanine, adenine, adenosine, agmatine, alanine, allantoin, asparagine, aspartic acid, asymmetric dimethylarginine, beta-alanine, betaine, cadaverine, carnosine, choline, cis-hydroxyproline, citrulline, creatine, creatinine, cytidine, cytosine, deoxyadenosine, deoxycytidine, deoxyguanosine, deoxyinosine, deoxyuridine, dimethylamine, dopamine, epinephrine, ethanolamine, gamma-aminobutyric acid, glutamic acid, glycine, guanine, guanosine, histamine, histidine, homocysteine, homoarginine, hypoxanthine, indole, indole-3-acetamide, inosine, isoleucine, alpha-aminobutyric acid, arginine, cystathionine, DOPA, leucine, glutamine, kynurenine, lysine, methionine, methionine-sulfoxide, methylhistidine, N1-acetylspermidine, N2-acetyl-ornithine, N-acetylputrescine, nicotinamide ribotide, norepinephrine, nudifloramide, ornithine, phenylalanine, phenylethylamine, proline, putrescine, sarcosine, serine, serotonin, spermidine, spermine, taurine, threonine, thymidine, thymine, trans-hydroxyproline, trimethylamine, trimethylamine N-oxide, tryptamine, tryptophan, tyramine, tyrosine, uracil, urea, uridine, valine, homocitrulline, methylamine, 2-hydroxy-2-methylbutyric acid, 2-hydroxy-3-methylvaleric acid, 2-hydroxybutyric acid, 2-hydroxyglutaric acid, 2-hydroxyisobutyric acid, 2-hydroxyisovaleric acid, 2-hydroxyphenylacetic acid, 2-oxoadipic acid, 2-oxoisocaproic acid, 3,4-dihydroxybutyric acid, 3-aminoisobutyric acid, 3-carboxy-4-methyl-5-propyl-2-furanpropionic acid, 3-deoxyglucosone, 3-hydroxybutyric acid, 3-hydroxyisobutyric acid, 3-hydroxyisovaleric acid, 3-hydroxyphenylacetic acid, 3-indoleacetic acid, 3-methyladipic acid, 4-ethylphenyl sulfate, 4-hydroxybenzoic acid, 4-hydroxyphenylacetic acid, 4-hydroxyphenylpyruvic acid, 5-

aminolevulinic Acid, 5-hydroxyindoleacetic Acid, 5-oxoproline, acetoacetic acid, alpha-ketoglutaric acid, alpha-ketoisovaleric acid, argininic acid, benzoic acid, butyric acid, isobutyric acid, caffeic acid, caproic acid, caprylic acid, cis-aconitic acid, citric acid, cyclic AMP, dimethylglycine, ethylmalonic acid, fumaric acid, furan-2,5-dicarboxylic acid, glutaric Acid, glyceric acid, guanidinopropionic acid, guanidoacetic acid, hippuric acid, homovanillic acid, indole-3-carboxylic acid, indole-3-propionic acid, indolelactic acid, indoxyl glucuronide, indoxyl sulfate, indoxyl-D-glucoside, isocitric acid, isovaleric acid, kynurenic acid, lactic acid, maleic acid, malic acid, malonic acid, methylmalonic acid, N1-acetyl-lysine, N6-acetyl-lysine, N-acetyl-glycine, N-acetyl-alanine, N-acetyl-arginine, N-acetyl-asparagine, N-acetyl-aspartic acid, N-acetyl-glutamic acid, N-acetyl-glutamine, N-acetyl-histidine, N-acetyl-isoleucine, N-acetyl-leucine, N-acetyl-methionine, N-acetyl-serine, N-acetyl-tryptophan, N-acetyl-tyrosine, N-acetyl-valine, N-acetyl-proline, N-methyl-aspartic acid, orotic acid, p-cresol sulfate, phenylacetic acid, phenylacetylglutamine, picolinic acid, pipecolic acid, propionic acid, pyruvic acid, quinaldic acid, quinoline-4-carboxylic acid, quinolinic acid, salicylic acid, shikimic acid, succinic acid, tartaric acid, threonic acid, tiglylglycine, uric acid, valeric acid, xanthine, xanthosine, L-carnitine inner salt, acetyl-L-carnitine hydrochloride, propionyl-L-carnitine, butyryl-L-carnitine, hexanoyl-L-carnitine, octanoyl-L-carnitine, decanoyl-L-carnitine, dodecanoyl-L-carnitine, tetradecanoyl-L-carnitine, hexadecanoyl-L-carnitine, octadecanoyl-L-carnitine, N-stearoyl-D-erythro-sphingosylphosphorylcholine, 1,2-dilinolenoyl-sn-glycero-3-phosphocholine, 1,2-Dioctadecanoyl-sn-glycero-3-phosphocholine, 1-oleoyl-2-hydroxy-sn-glycero-3-phosphocholine, C18 ceramide (d18:1/18:0), C18 glucosyl(β) ceramide (d18:1/18:0), C18 Lactosyl(β) Ceramide (d18:1/18:0), 17:0 cholesteryl ester, 18:1 DG, glyceryl trioleate, and glucose were all bought from Sigma-Aldrich (Oakville, ON, CA). N^1, N^{12}-diacetylspermine hydrochloride was purchased from Cayman Chemical (Ann Arbor, MI, U.S.A.). 4-hydroxyhippuric acid was obtained

from Toronto Research Chemicals (North York, ON, CA). Other stable isotope (D, ^{13}C, ^{15}N)-labelled internal standards (ISTD) including D_2-ornithine, ^{15}N-histidine, D_3-creatinine, D_3-DOPA, D_4-dopamine, ^{13}C-tyrosine, ^{13}C-D_3-methionine, D_3-proline, D_4-serotonin, D_4-putrescine, D_3-sarcosine, $^{13}C_2$-taurine, D_4-tyramine, ^{15}N-alanine, $^{13}C_6$-arginine, ^{15}N-asparagine, D_3-aspartic acid, D_7-citrulline, D_3-glutamic acid, D_5-glutamine, $^{13}C_2$-glycine, ^{13}C-leucine, ^{15}N-phenylalanine, ^{13}C-serine, D_2-threonine, $^{15}N_2$-tryptophan, D_8-valine, D_9-TMAO, D_6-N1-acetylspermidine, D_3-nudifloramide, D_5-idole-3-acetamide, D_4-3-methoxytyramine, D_4-ethanolamine, D_4-tryptamine, D_4-lysine, $^{13}C_2$, $^{15}N_4$-Allantoin, D_3-methylamine, D_4-1,3-diaminopropane, D_2-uridine, $^{15}N_4$-hypoxanthine, $^{13}C_6$-indoxyl sulfate, D_7-p-cresol sulfate, and $^{15}N_2$-uric acid were bought from Cambridge Isotope Laboratories Inc. (Tewksbury, MA, U.S.A.). D_8-spermine tetrahydrochloride, D_8-spermidine trihydrochloride were purchased from IsoSciences (Ambler, PA, U.S.A.). D_6-N^1, N^{12}-diacetylspermine dihydrochloride and D_6-ADMA were bought from Toronto Research Chemicals (North York, ON, CA). D_3-creatinine, D_9-choline chloride, and D_9-betaine hydrochloride were purchased from C/D/N Isotopes Inc. (Pointe-Claire, QC, CA). Labelled carnitine standard set was purchased from Cambridge Isotope Laboratories Inc. (Tewksbury, MA, U.S.A.). 1,2-dimyristoyl-sn-glycero-3-phosphocholine, 1,2-diarachidoyl-sn-glycero-3-phosphocholine, 1-nonanoyl-2-hydroxy-sn-glycero-3-phosphocholine, N-hexanoyl-D-erythro-sphingosylphosphorylcholine, 16:0 cholesteryl-D7 ester, 1,3-17:0 D5 DG, 16:0-18:0-16:0 D5 TG, Cer(d18:0/12:0(OH)), GlcCer(d18:1/12:0), and $^{13}C_6$-glucose were bought from Sigma-Aldrich (Oakville, ON, CA).

Supplementary Table S1. Concentration of Standards at Different Calibration Levels

Analyte	Concentration in µM						
	**Cal1**	**Cal2**	**Cal3**	**Cal4**	**Cal5**	**Cal6**	**Cal7**
**PITC Panel**							
1,3-Diaminopropane	0.075	0.15	0.75	1.5	3	4.5	6
1-Methylnicotinamide	0.075	0.15	0.75	1.5	3	4.5	6
3-Methoxytyramine	0.1	0.2	1	2	4	6	8
3-Nitrotyrosine	1	2	10	20	40	60	80
5-Methoxytryptamine	0.1	0.2	1	2	4	6	8
5-Methyluridine	0.05	0.1	0.5	1	2	3	4
7-Methylguanine	0.0125	0.025	0.125	0.25	0.5	0.75	1
Adenine	0.05	0.1	0.5	1	2	3	4
Adenosine	0.075	0.15	0.75	1.5	3	4.5	6
Agmatine	0.1	0.2	1	2	4	6	8
Alanine	20	40	200	400	800	1200	1600
Allantoin	0.3	0.6	3	6	12	18	24
alpha-Aminoadipic acid	1	2	10	20	40	60	80
alpha-Aminobutyric acid	2	4	20	40	80	120	160
Arginine	5	10	50	100	200	300	400
Asparagine	5	10	50	100	200	300	400
Aspartic acid	5	10	50	100	200	300	400
Asymmetric dimethylarginine	0.25	0.5	2.5	5	10	15	20
beta-Alanine	1	2	10	20	40	60	80
Betaine	5	10	50	100	200	300	400
Cadaverine	0.09375	0.1875	0.9375	1.875	3.75	5.625	7.5
Carnosine	0.5	1	5	10	20	30	40
Choline	2.4	4.8	24	48	96	144	192

cis-4-Hydroxyproline	1	2	10	20	40	60	80
Citrulline	5	10	50	100	200	300	400
Creatine	2.5	5	25	50	100	150	200
Creatinine	10	20	100	200	400	600	800
Cystathionine	1	2	10	20	40	60	80
Cytidine	0.25	0.5	2.5	5	10	15	20
Cytosine	0.25	0.5	2.5	5	10	15	20
Deoxyadenosine	0.125	0.25	1.25	2.5	5	7.5	10
Deoxycytidine	0.5	1	5	10	20	30	40
Deoxyguanosine	0.16	0.32	1.6	3.2	6.4	9.6	12.8
Deoxyinosine	0.1	0.2	1	2	4	6	8
Deoxyuridine	1	2	10	20	40	60	80
Dimethylamine	2.5	5	25	50	100	150	200
DOPA	0.5	1	5	10	20	30	40
Dopamine	1	2	10	20	40	60	80
Epinephrine	0.1	0.2	1	2	4	6	8
Ethanolamine	1	2	10	20	40	60	80
gamma-Aminobutyric acid	0.05	0.1	0.5	1	2	3	4
Glutamic acid	10	20	100	200	400	600	800
Glutamine	20	40	200	400	800	1200	1600
Glycine	25	50	250	500	1000	1500	2000
Guanine	0.05	0.1	0.5	1	2	3	4
Guanosine	0.05	0.1	0.5	1	2	3	4
Histamine	1	2	10	20	40	60	80
Histidine	5	10	50	100	200	300	400
Homoarginine	0.2	0.4	2	4	8	12	16
Homocitrulline	0.5	1	5	10	20	30	40
Hypoxanthine	2	4	20	40	80	120	160
Indole	0.625	1.25	6.25	12.5	25	37.5	50
Indole-3-acetamide	0.125	0.25	1.25	2.5	5	7.5	10
Inosine	0.8	1.6	8	16	32	48	64

Isoleucine	5	10	50	100	200	300	400
Kynurenine	1	2	10	20	40	60	80
Leucine	5	10	50	100	200	300	400
Lysine	10	20	100	200	400	600	800
Methionine	5	10	50	100	200	300	400
Methionine sulfoxide	1	2	10	20	40	60	80
Methylamine	0.075	0.15	0.75	1.5	3	4.5	6
Methylhistidine	1	2	10	20	40	60	80
N1,N12-Diacetylspermine	0.25	0.5	2.5	5	10	15	20
N1-Acetylspermidine	0.1	0.2	1	2	4	6	8
N2-Acetyl-Ornithine	0.5	1	5	10	20	30	40
N-Acetylputrescine	0.025	0.05	0.25	0.5	1	1.5	2
Nicotinamide ribotide	0.6	1.2	6	12	24	36	48
Norepinephrine	0.125	0.25	1.25	2.5	5	7.5	10
Nudifloramide	0.75	1.5	7.5	15	30	45	60
Ornithine	5	10	50	100	200	300	400
Phenylalanine	5	10	50	100	200	300	400
Phenylethylamine	1	2	10	20	40	60	80
Proline	10	20	100	200	400	600	800
Putrescine	0.1	0.2	1	2	4	6	8
Sarcosine	1	2	10	20	40	60	80
Serine	5	10	50	100	200	300	400
Serotonin	0.1	0.2	1	2	4	6	8
Spermidine	0.25	0.5	2.5	5	10	15	20
Spermine	0.25	0.5	2.5	5	10	15	20
Taurine	2.5	5	25	50	100	150	200
Threonine	5	10	50	100	200	300	400
Thymidine	0.05	0.1	0.5	1	2	3	4
Thymine	0.8	1.6	8	16	32	48	64
Total dimethylarginine	0.25	0.5	2.5	5	10	15	20
trans-4-Hydroxyproline	1	2	10	20	40	60	80

Trimethylamine	5	10	50	100	200	300	400
Trimethylamine N-Oxide	10	20	100	200	400	600	800
Tryptamine	0.025	0.05	0.25	0.5	1	1.5	2
Tryptophan	5	10	50	100	200	300	400
Tyramine	0.025	0.05	0.25	0.5	1	1.5	2
Tyrosine	5	10	50	100	200	300	400
Uracil	0.25	0.5	2.5	5	10	15	20
Urea	125	250	1250	2500	5000	7500	10000
Uridine	0.4	0.8	4	8	16	24	32
Valine	10	20	100	200	400	600	800
**3-NPH Panel**							
2,5-Furandicarboxylic acid	0.1	0.2	1	2	4	6	8
2-Hydroxy-2-methylbutyric acid	0.125	0.25	1.25	2.5	5	7.5	10
2-Hydroxy-3-methylvaleric acid	0.125	0.25	1.25	2.5	5	7.5	10
2-Hydroxybutyric acid	2	4	20	40	80	120	160
2-Hydroxyglutaric acid	0.0625	0.125	0.625	1.25	2.5	3.75	5
2-Hydroxyisobutyric acid	0.125	0.25	1.25	2.5	5	7.5	10
2-Hydroxyisovaleric acid	0.5	1	5	10	20	30	40
2-Hydroxyphenylacetic acid	0.078125	0.15625	0.78125	1.5625	3.125	4.6875	6.25
2-Oxoadipic acid	1.25	2.5	12.5	25	50	75	100
2-Oxoisocaproic acid	3.2	6.4	32	64	128	192	256
3-(3-Hydroxyphenyl)-3-hydroxypropanoic acid	0.03125	0.0625	0.3125	0.625	1.25	1.875	2.5
3,4-Dihydroxybutyric acid	1	2	10	20	40	60	80
3-Aminoisobutyric acid	1	2	10	20	40	60	80
3-Carboxy-4-methyl-5-propyl-2-furanpropionic acid	1.25	2.5	12.5	25	50	75	100
3-Deoxyglucosone	0.03125	0.0625	0.3125	0.625	1.25	1.875	2.5
3-Hydroxybutyric acid	5	10	50	100	200	300	400
3-Hydroxyisobutyric acid	0.625	1.25	6.25	12.5	25	37.5	50
3-Hydroxyisovaleric acid	0.5	1	5	10	20	30	40
3-Hydroxyphenylacetic acid	0.125	0.25	1.25	2.5	5	7.5	10

3-Indoleacetic acid	0.3125	0.625	3.125	6.25	12.5	18.75	25
3-Methyladipic acid	0.03125	0.0625	0.3125	0.625	1.25	1.875	2.5
4-Ethylphenyl sulfate	0.125	0.25	1.25	2.5	5	7.5	10
4-Hydroxybenzoic acid	0.15625	0.3125	1.5625	3.125	6.25	9.375	12.5
4-Hydroxyhippuric acid	0.1	0.2	1	2	4	6	8
4-Hydroxyphenylacetic acid	0.05	0.1	0.5	1	2	3	4
4-Hydroxyphenylpyruvic acid	1	2	10	20	40	60	80
5-Aminolevulinic Acid	1	2	10	20	40	60	80
5-Hydroxyindoleacetic acid	0.02	0.04	0.2	0.4	0.8	1.2	1.6
5-Oxoproline	3	6	30	60	120	180	240
Acetoacetic acid	1.875	3.75	18.75	37.5	75	112.5	150
alpha-Ketoglutaric acid	0.5	1	5	10	20	30	40
alpha-Ketoisovaleric acid	0.45	0.9	4.5	9	18	27	36
Argininic acid	1	2	10	20	40	60	80
Benzoic acid	0.125	0.25	1.25	2.5	5	7.5	10
Butyric acid	0.1	0.2	1	2	4	6	8
Isobutyric acid	0.125	0.25	1.25	2.5	5	7.5	10
Caffeic acid	0.15625	0.3125	1.5625	3.125	6.25	9.375	12.5
Caproic acid	0.25	0.5	2.5	5	10	15	20
Caprylic acid	0.125	0.25	1.25	2.5	5	7.5	10
cis-Aconitic acid	0.15625	0.3125	1.5625	3.125	6.25	9.375	12.5
Citric acid	2.5	5	25	50	100	150	200
Cyclic AMP	0.2	0.4	2	4	8	12	16
Dimethylglycine	0.625	1.25	6.25	12.5	25	37.5	50
Ethylmalonic acid	0.125	0.25	1.25	2.5	5	7.5	10
Fumaric acid	0.125	0.25	1.25	2.5	5	7.5	10
Glutaric acid	0.0625	0.125	0.625	1.25	2.5	3.75	5
Glyceric acid	0.15	0.3	1.5	3	6	9	12
Guanidinopropionic acid	1	2	10	20	40	60	80
Guanidoacetic acid	0.5	1	5	10	20	30	40
Hippuric acid	0.5	1	5	10	20	30	40

Homovanillic acid	0.0125	0.025	0.125	0.25	0.5	0.75	1
Indole-3-carboxylic acid	0.5	1	5	10	20	30	40
Indole-3-propionic acid	0.0625	0.125	0.625	1.25	2.5	3.75	5
Indolelactic acid	0.0375	0.075	0.375	0.75	1.5	2.25	3
Indoxyl glucoside	1	2	10	20	40	60	80
Indoxyl glucuronide	0.125	0.25	1.25	2.5	5	7.5	10
Indoxyl sulfate	0.375	0.75	3.75	7.5	15	22.5	30
Isocitric acid	0.15625	0.3125	1.5625	3.125	6.25	9.375	12.5
Isovaleric acid	0.6	1.2	6	12	24	36	48
Kynurenic acid	0.0625	0.125	0.625	1.25	2.5	3.75	5
Lactic acid	100	200	1000	2000	4000	6000	8000
Maleic acid	0.125	0.25	1.25	2.5	5	7.5	10
Malic acid	0.5	1	5	10	20	30	40
Malonic acid	0.09375	0.1875	0.9375	1.875	3.75	5.625	7.5
Methylmalonic acid	0.05	0.1	0.5	1	2	3	4
N1-Acetyl-Lysine	0.5	1	5	10	20	30	40
N6-Acetyl-Lysine	0.5	1	5	10	20	30	40
N-Acetyl-Alanine	0.125	0.25	1.25	2.5	5	7.5	10
N-Acetyl-Arginine	0.5	1	5	10	20	30	40
N-Acetyl-Asparagine	0.0625	0.125	0.625	1.25	2.5	3.75	5
N-Acetyl-Aspartic acid	0.0625	0.125	0.625	1.25	2.5	3.75	5
N-Acetyl-Glutamic acid	0.0625	0.125	0.625	1.25	2.5	3.75	5
N-Acetyl-Glutamine	0.5	1	5	10	20	30	40
N-Acetyl-Glycine	0.5	1	5	10	20	30	40
N-Acetyl-Histidine	2.5	5	25	50	100	150	200
N-Acetyl-Isoleucine	0.125	0.25	1.25	2.5	5	7.5	10
N-Acetyl-Leucine	0.125	0.25	1.25	2.5	5	7.5	10
N-Acetyl-Methionine	0.125	0.25	1.25	2.5	5	7.5	10
N-Acetyl-Proline	0.0625	0.125	0.625	1.25	2.5	3.75	5
N-Acetyl-Serine	0.125	0.25	1.25	2.5	5	7.5	10
N-Acetyl-Tryptophan	0.125	0.25	1.25	2.5	5	7.5	10

N-Acetyl-Tyrosine	0.0625	0.125	0.625	1.25	2.5	3.75	5
N-Acetyl-Valine	0.0625	0.125	0.625	1.25	2.5	3.75	5
N-Methyl-Aspartic acid	1.25	2.5	12.5	25	50	75	100
Orotic acid	0.28125	0.5625	2.8125	5.625	11.25	16.875	22.5
p-Cresol sulfate	20	40	200	400	800	1200	1600
Phenylacetic acid	0.15625	0.3125	1.5625	3.125	6.25	9.375	12.5
Phenylacetylglutamine	0.0625	0.125	0.625	1.25	2.5	3.75	5
Picolinic acid	0.6	1.2	6	12	24	36	48
Pipecolic acid	0.16	0.32	1.6	3.2	6.4	9.6	12.8
Propionic acid	0.125	0.25	1.25	2.5	5	7.5	10
Pyruvic acid	2.5	5	25	50	100	150	200
Quinaldic acid	0.5	1	5	10	20	30	40
Quinoline-4-carboxylic acid	0.25	0.5	2.5	5	10	15	20
Quinolinic acid	0.0625	0.125	0.625	1.25	2.5	3.75	5
Salicylic acid	0.5	1	5	10	20	30	40
Shikimic acid	0.2	0.4	2	4	8	12	16
Succinic acid	1	2	10	20	40	60	80
Tartaric acid	0.05	0.1	0.5	1	2	3	4
Threonic acid	1.5	3	15	30	60	90	120
Tiglylglycine	0.1	0.2	1	2	4	6	8
Uric acid	20	40	200	400	800	1200	1600
Valeric acid	0.125	0.25	1.25	2.5	5	7.5	10
Xanthine	0.5	1	5	10	20	30	40
Xanthosine	0.5	1	5	10	20	30	40

Supplementary Table S2. Optimized MRM Transitions and Mass Spectrometer Parameters

Analyte	MRM Parameters				
	Q1	Q3	DP	CE	CXP
1,3-Diaminopropane	345.1	252	60	15	15
1-Methylnicotinamide	137.1	91.9	70	28	15
3-Methoxytyramine	303.1	151.1	76	25	15
3-Nitrotyrosine	362.2	136.1	65	41	15
5-Methoxytryptamine	326	174.3	41	27	10
5-Methyluridine	259.1	127	88	19	13
7-Methylguanine	166.1	149	132	29	13
Adenine	136.2	118.9	125	33	12
Adenosine	268.2	136.1	87	28	12
Agmatine	266	114	46	29	5
Alanine	225.2	44.2	61	35	15
Allantoin	159	116	83	11	15
alpha-Aminoadipic acid	297.1	144.2	46	23	15
alpha-Aminobutyric acid	239.1	104	45	16	15
Arginine	310	217	76	23	15
Asparagine	268.2	87	41	25	15
Aspartic acid	269.2	116.2	76	21	15
Asymmetric dimethylarginine	338.2	46	71	61	15
beta-Alanine	225.1	90.1	41	16	15
Betaine	118.1	59.2	60	30	15
Cadaverine	238.2	145	90	17	15
Carnosine	362.2	110.1	46	47	15
Choline	104.2	60.1	28	50	15
cis-4-Hydroxyproline	267.1	68	56	61	15
Citrulline	311.2	113.1	56	29	15

Creatine	132.1	90	40	18	15
Creatinine	114.1	44	60	17	15
Cystathionine	358.1	134.1	60	29	15
Cytidine	244.2	112.1	50	22	8
Cytosine	112.1	95	101	24	10
Deoxyadenosine	252.1	136.1	97	19	13
Deoxycytidine	228.1	112	61	23	13
Deoxyguanosine	268.1	152	90	17	13
Deoxyinosine	253.1	137.1	77	15	13
Deoxyuridine	229.1	113	140	17	13
Dimethylamine	181	88	46	21	14
DOPA	333.1	198.1	61	19	15
Dopamine	289.2	137.2	56	29	15
Epinephrine	319.1	166.2	40	31	12
Ethanolamine	197	62	31	21	10
gamma-Aminobutyric acid	239	104	65	17	15
Glutamic acid	283.2	130.2	76	23	15
Glutamine	282.2	130	71	25	15
Glycine	211.2	75.9	66	14	15
Guanine	152.1	135.1	110	27	15
Guanosine	284.1	152	90	21	12
Histamine	247	154	56	19	15
Histidine	291.1	110.2	56	33	15
Homoarginine	324.1	84	112	46	12
Homocitrulline	325	127	56	29	10
Hypoxanthine	137.1	110.1	81	29	15
Indole	118.1	91	105	30	10
Indole-3-acetamide	175	130	60	20	15
Inosine	269.1	136.9	81	26	13
Isoleucine	267.3	69	55	45	15
Kynurenine	344.2	146.2	46	33	15

Leucine	267.3	43	55	66	15
Lysine	417.2	324.2	81	19	15
Methionine	285.1	104.2	71	25	15
Methionine sulfoxide	301.2	88.1	61	41	15
Methylamine	167	94	69	19	10
Methylhistidine	305.1	212.1	46	23	15
N1,N12-Diacetylspermine	422.4	171.2	32	34	15
N1-Acetylspermidine	458.1	323	40	17	15
N2-Acetyl-Ornithine	310.2	217.3	46	15	15
N-Acetylputrescine	266	173	41	19	8
Nicotinamide ribotide	335	123	72	21	10
Norepinephrine	305.2	152	36	29	10
Nudifloramide	153	108	86	29	13
Ornithine	403.2	310.2	76	19	15
Phenylalanine	301.2	120.2	71	31	15
Phenylethylamine	257.2	105.2	71	31	15
Proline	251.2	70.3	51	43	15
Putrescine	266.1	113.9	100	25	15
Sarcosine	225.1	44.2	43	31	15
Serine	241.2	60	41	31	15
Serotonin	312.3	160.2	66	27	15
Spermidine	551.2	193.2	71	41	15
Spermine	743.3	193.2	56	61	15
Taurine	261	126.1	71	21	15
Threonine	255.2	74.1	66	29	15
Thymidine	243.1	127.1	50	17	12
Thymine	127.1	110.1	62	21	15
Total dimethylarginine	76	58	40	26	15
trans-4-Hydroxyproline	338.2	70.1	71	61	15
Trimethylamine	59.9	44	125	22	13
Trimethylamine N-Oxide	267.2	68	56	61	15

Tryptamine	296	144	91	27	12
Tryptophan	340.2	188.2	96	25	15
Tyramine	273.2	121.1	41	29	15
Tyrosine	317.2	136.1	76	31	15
Uracil	113.1	70	91	21	10
Urea	61	44	80	26	10
Uridine	245.1	113	80	21	17
Valine	253.2	72.2	81	31	15
2,5-Furandicarboxylic acid	425.1	150	-202	-30	-11
2-Hydroxy-2-methylbutyric acid	252	137	-131	-26	-15
2-Hydroxy-3-methylvaleric acid	266	151.9	-149	-27	-10
2-Hydroxybutyric acid	238	137	-101	-26	-17
2-Hydroxyglutaric acid	417.1	137	-108	-30	-8
2-Hydroxyisobutyric acid	238	152	-120	-20	-10
2-Hydroxyisovaleric acid	252	152	-139	-22	-13
2-Hydroxyphenylacetic acid	286	137	-249	-29	-8
2-Oxoadipic acid	429	137	-160	-38	-8
2-Oxoisocaproic acid	399.1	150	-169	-30	-10
3-(3-Hydroxyphenyl)-3-hydroxypropanoic acid	316.2	194.2	-130	-20	-9
3,4-Dihydroxybutyric acid	254	137	-135	-19	-15
3-Aminoisobutyric acid	237.1	208	-166	-21	-12
3-Carboxy-4-methyl-5-propyl-2-furanpropionic acid	509	150	-90	-40	-15
3-Deoxyglucosone	431.1	136.9	-153	-33	-10
3-Hydroxybutyric acid	238	193.8	-58	-21	-6
3-Hydroxyisobutyric acid	238.1	164.9	-126	-18	-13
3-Hydroxyisovaleric acid	252.1	152	-90	-26	-14
3-Hydroxyphenylacetic acid	286	150	-150	-25	-8
3-Indoleacetic acid	308.9	152.1	-100	-28	-16
3-Methyladipic acid	429	178	-200	-34	-15
4-Ethylphenyl sulfate	201	121	-70	-26	-15

4-Hydroxybenzoic acid	272	150	-83	-28	-15
4-Hydroxyhippuric acid	328.9	208.8	-91	-28	-9
4-Hydroxyphenylacetic acid	286	150.1	-80	-27	-7
4-Hydroxyphenylpyruvic acid	449.1	178	-90	-30	-10
5-Aminolevulinic Acid	400.1	137	-60	-25	-10
5-Hydroxyindoleacetic acid	325	132.1	-84	-30	-5
5-Oxoproline	263	125	-117	-26	-10
Acetoacetic acid	371	137	-73	-30	-5
alpha-Ketoglutaric acid	550.1	232.7	-104	-36	-24
alpha-Ketoisovaleric acid	385	150.1	-125	-30	-12
Argininic acid	309	152	-60	-30	-15
Benzoic acid	256	150	-60	-26	-9
Butyric acid + Isobutyric acid	221.9	136.8	-68	-30	-5
Caffeic acid	314	176.1	-90	-31	-7
Caproic acid	250	137.1	-75	-25	-5
Caprylic acid	278.1	137.1	-80	-28	-5
cis-Aconitic acid	578	424.8	-124	-22	-11
Citric acid	596.3	221.9	-92	-50	-16
Cyclic AMP	328	134	-50	-33	-9
Dimethylglycine	237.1	137.1	-147	-24	-8
Ethylmalonic acid	401	178	-166	-25	-12
Fumaric acid	385	234	-78	-20	-9
Glutaric acid	401.1	247.9	-80	-26	-10
Glyceric acid	240.1	137	-130	-25	-17
Guanidinopropionic acid	265	223	-100	-20	-15
Guanidoacetic acid	251	209	-90	-20	-10
Hippuric acid	313	136.9	-65	-26	-11
Homovanillic acid	316	149.8	-79	-26	-10
Indole-3-carboxylic acid	295	116	-90	-28	-16
Indole-3-propionic acid	323.2	194	-181	-23	-12
Indolelactic acid	339.1	137	-145	-29	-10

Indoxyl glucoside	294	132	-80	-20	-15
Indoxyl glucuronide	443	250	-110	-24	-15
Indoxyl sulfate	212	80	-170	-60	-10
Isocitric acid	596	234	-90	-40	-15
Isovaleric acid	236.1	137.1	-75	-24	-5
Kynurenic acid	323	150	-85	-28	-15
Lactic acid	224	137	-55	-12	-10
Maleic acid	385.1	234	-202	-25	-13
Malic acid	403	207.9	-60	-26	-12
Malonic acid	373	178	-160	-23	-15
Methylmalonic acid	387	177.9	-85	-28	-9
N1-Acetyl-Lysine	322	280	-64	-27	-12
N6-Acetyl-Lysine	322.2	136.9	-64	-33	-8
N-Acetyl-Alanine	265.1	137	-145	-31	-11
N-Acetyl-Arginine	350.1	137	-200	-40	-11
N-Acetyl-Asparagine	308.2	155.2	-150	-20	-9
N-Acetyl-Aspartic acid	444.1	291	-188	-22	-21
N-Acetyl-Glutamic acid	458.2	263	-80	-31	-15
N-Acetyl-Glutamine	322.2	137.1	-64	-35	-10
N-Acetyl-Glycine	251.1	137	-124	-27	-9
N-Acetyl-Histidine	331.2	194	-155	-20	-10
N-Acetyl-Isoleucine	307.2	136.9	-165	-32	-8
N-Acetyl-Leucine	307.1	137	-168	-32	-8
N-Acetyl-Methionine	325.1	137	-165	-31	-10
N-Acetyl-Proline	291.1	249	-101	-24	-12
N-Acetyl-Serine	281.1	251	-82	-18	-13
N-Acetyl-Tryptophan	380.2	137	-56	-42	-11
N-Acetyl-Tyrosine	357.1	251	-171	-25	-13
N-Acetyl-Valine	293.1	137	-169	-32	-15
N-Methyl-Aspartic acid	416.1	263	-61	-23	-13
Orotic acid	290	150	-85	-32	-15

p-Cresol sulfate	187	80	-95	-32	-13
Phenylacetic acid	270	152	-144	-22	-7
Phenylacetylglutamine	398.1	127	-250	-52	-15
Picolinic acid	257	150	-135	-24	-17
Pipecolic acid	263.1	137	-67	-29	-15
Propionic acid	208	136.8	-30	-26	-25
Pyruvic acid	357	136.8	-80	-33	-5
Quinaldic acid	307.1	150	-85	-30	-15
Quinoline-4-carboxylic acid	307	150	-100	-25	-15
Quinolinic acid	436	283	-100	-40	-15
Salicylic acid	272	117.9	-65	-46	-7
Shikimic acid	308	290	-67	-20	-6
Succinic acid	387.1	234	-100	-40	-20
Tartaric acid	419	137	-242	-55	-15
Threonic acid	270.1	152	-155	-33	-15
Tiglylglycine	291.1	152	-150	-22	-11
Uric acid	167.1	124.1	-40	-20	-6
Valeric acid	236	137	-75	-24	-5
Xanthine	151	108	-80	-22	-15
Xanthosine	283.2	151	-72	-25	-15
C0	162.1	85.1	61	27	15
C10	316.2	85.1	56	37	15
C10:1	314.2	85.1	68	40	15
C10:2	312.2	85.1	67	40	15
C12	344.3	85.1	73	44	15
C12:1	342.2	85.1	73	44	15
C12DC	374.3	85.1	86	45	15
C14	372.2	85.1	86	45	15
C14:1	370.3	85.1	78	47	15
C14:1OH	386.3	85.1	81	50	15
C14:2	368.3	85.1	78	47	15

C14:2OH	384.3	85.1	81	49	15
C16	400.3	85.1	84	51	15
C16:1	398.3	85.1	84	51	15
C16:1OH	414.3	85.1	87	53	15
C16:2	396.3	85.1	83	51	15
C16:2OH	412.3	85.1	86	53	15
C16OH	416.3	85.1	87	53	15
C18	428.4	85.1	96	63	15
C18:1	426.4	85.1	89	55	15
C18:1OH	442.4	85.1	92	57	15
C18:2	424.3	85.1	89	54	15
C2	204.1	85.1	41	27	15
C3	218.1	85.1	46	29	15
C3:1	216.1	85.1	49	27	15
C3OH	234.1	85.1	53	30	15
C4	232.2	85.1	46	29	15
C4:1	230.1	85.1	52	29	15
C4OH	248.1	85.1	55	32	15
C5	246.2	85.1	46	29	15
C5:1	244.2	85.1	55	31	15
C5:1DC	274.1	85.1	60	35	15
C5DC	276.2	85.1	61	35	15
C5MDC	290.2	85.1	63	37	15
C5OH	262.2	85.1	58	33	15
C6	260.2	85.1	56	27	15
C6:1	258.1	85.1	57	33	15
C7DC	304.2	85.1	66	39	15
C8	288.2	85.1	66	33	15
C9	302.2	85.1	66	39	15
CE(14:0)	614.587	369.352	41	19	10
CE(14:1)	612.571	369.352	41	19	10

CE(15:0)	628.603	369.352	41	19	10
CE(15:1)	626.587	369.352	41	19	10
CE(16:0)	642.618	369.352	41	19	10
CE(16:1)	640.603	369.352	41	19	10
CE(17:0)	656.634	369.352	71	21	10
CE(17:1)	654.618	369.352	72	22	10
CE(18:0)	670.65	369.352	73	23	10
CE(18:1)	668.634	369.352	56	19	10
CE(18:2)	666.618	369.352	56	21	10
CE(18:3)	664.603	369.352	56	21	10
CE(20:0)	698.681	369.352	56	21	10
CE(20:1)	696.665	369.352	56	21	10
CE(20:3)	692.634	369.352	56	21	10
CE(20:4)	690.618	369.352	61	19	10
CE(20:5)	688.603	369.352	61	19	10
CE(22:0)	726.712	369.352	61	19	10
CE(22:1)	724.697	369.352	61	21	10
CE(22:2)	722.681	369.352	61	21	10
CE(22:5)	716.634	369.352	61	21	10
CE(22:6)	714.618	369.352	61	21	10
Cer(d16:1/18:0)	520.509	236.213	206	33	10
Cer(d16:1/20:0)	548.54	236.213	211	35	10
Cer(d16:1/22:0)	576.571	236.213	226	39	10
Cer(d16:1/23:0)	590.587	236.213	226	39	10
Cer(d16:1/24:0)	604.603	236.213	246	39	10
Cer(d18:0/18:0(OH))	584.561	284.271	115	35	10
Cer(d18:0/18:0)	568.566	284.271	121	45	10
Cer(d18:0/20:0)	596.598	284.271	121	45	10
Cer(d18:0/22:0)	624.629	284.271	121	45	10
Cer(d18:0/24:0)	652.66	284.271	131	49	10
Cer(d18:0/24:1)	650.645	284.271	131	49	10

Cer(d18:0/26:1(OH))	694.671	284.271	132	50	10
Cer(d18:0/26:1)	678.676	284.271	132	50	10
Cer(d18:1/14:0)	492.478	264.245	206	31	10
Cer(d18:1/16:0)	520.509	264.245	206	33	10
Cer(d18:1/18:0(OH))	582.546	264.245	211	35	10
Cer(d18:1/18:0)	548.54	264.245	211	35	10
Cer(d18:1/18:1)	546.524	264.245	211	35	10
Cer(d18:1/20:0(OH))	610.577	264.245	226	39	10
Cer(d18:1/20:0)	576.571	264.245	226	39	10
Cer(d18:1/22:0)	604.603	264.245	246	39	10
Cer(d18:1/23:0)	618.618	264.245	246	39	10
Cer(d18:1/24:0)	632.634	264.245	256	37	10
Cer(d18:1/24:1)	630.618	264.245	256	37	10
Cer(d18:1/25:0)	646.65	264.245	256	37	10
Cer(d18:1/26:0)	660.665	264.245	260	38	10
Cer(d18:1/26:1)	658.65	264.245	260	38	10
Cer(d18:2/14:0)	490.462	262.229	206	31	10
Cer(d18:2/16:0)	518.493	262.229	206	33	10
Cer(d18:2/18:0)	546.524	262.229	211	35	10
Cer(d18:2/18:1)	544.509	262.229	211	35	10
Cer(d18:2/20:0)	574.556	262.229	226	39	10
Cer(d18:2/22:0)	602.587	262.229	226	39	10
Cer(d18:2/23:0)	616.603	262.229	246	39	10
Cer(d18:2/24:0)	630.618	262.229	256	37	10
Cer(d18:2/24:1)	628.603	262.229	256	37	10
DG(14:0/14:0)	530.478	285.242	40	31	10
DG(14:0/18:1)	584.525	339.289	41	29	10
DG(14:0/18:2)	582.509	337.274	41	29	10
DG(14:0/20:0)	614.572	285.242	56	29	10
DG(14:1/18:1)	582.509	339.289	41	29	10
DG(14:1/20:2)	608.525	283.227	56	29	10

DG(16:0/16:0)	586.541	313.274	41	29	10
DG(16:0/16:1)	584.525	311.258	41	29	10
DG(16:0/18:1)	612.556	339.289	56	29	10
DG(16:0/18:2)	610.541	337.274	56	29	10
DG(16:0/20:0)	642.603	313.274	56	37	10
DG(16:0/20:3)	636.556	363.289	56	37	10
DG(16:0/20:4)	634.541	361.274	56	37	10
DG(16:1/18:0)	612.556	311.258	56	29	10
DG(16:1/18:1)	610.541	339.289	56	29	10
DG(16:1/18:2)	608.525	337.274	56	29	10
DG(16:1/20:0)	640.587	311.258	56	37	10
DG(17:0/17:1)	612.556	325.274	56	29	10
DG(17:0/18:1)	626.572	339.289	56	29	10
DG(18:0/20:0)	670.634	341.305	66	39	10
DG(18:0/20:4)	662.572	361.274	66	33	10
DG(18:1/18:1)	638.572	339.289	56	37	10
DG(18:1/18:2)	636.556	337.274	56	37	10
DG(18:1/18:3)	634.541	335.258	56	37	10
DG(18:1/18:4)	632.525	333.242	56	37	10
DG(18:1/20:0)	668.619	339.289	66	33	10
DG(18:1/20:1)	666.603	367.321	66	33	10
DG(18:1/20:2)	664.587	365.305	66	33	10
DG(18:1/20:3)	662.572	363.289	66	33	10
DG(18:1/20:4)	660.556	361.274	66	33	10
DG(18:1/22:5)	686.572	387.289	66	33	10
DG(18:1/22:6)	684.556	385.273	66	33	10
DG(18:2/18:2)	634.541	337.274	56	37	10
DG(18:2/18:3)	632.525	335.258	56	37	10
DG(18:2/18:4)	630.509	333.242	56	37	10
DG(18:2/20:0)	666.603	337.274	66	33	10
DG(18:2/20:4)	658.541	361.274	66	33	10

DG(18:3/18:3)	630.509	335.258	56	37	10
DG(18:3/20:2)	660.556	365.305	66	33	10
DG(21:0/22:6)	728.619	385.274	76	37	10
DG(22:1/22:2)	748.681	393.336	76	37	10
DG-O(14:0/18:2)	568.53	323.294	41	29	10
DG-O(16:0/18:1)	598.577	325.31	56	29	10
DG-O(16:0/20:4)	620.561	347.294	56	37	10
LacCer(d18:1/14:0)	834.594	264.245	131	50	10
LacCer(d18:1/16:0)	862.625	264.245	131	51	10
LacCer(d18:1/18:0)	890.656	264.245	131	52	10
LacCer(d18:1/20:0)	918.688	264.245	131	52	10
LacCer(d18:1/22:0)	946.719	264.245	131	52	10
LacCer(d18:1/24:0)	974.75	264.245	131	53	10
LacCer(d18:1/24:1)	972.735	264.245	131	54	10
LacCer(d18:1/26:0)	1002.781	264.245	131	54	10
LacCer(d18:1/26:1)	1000.766	264.245	131	54	10
Trihexosylceramide(d18:1/16:0)	1024.678	264.245	131	51	10
Trihexosylceramide(d18:1/18:0)	1052.709	264.245	131	52	10
Trihexosylceramide(d18:1/20:0)	1080.74	264.245	131	52	10
Trihexosylceramide(d18:1/24:1)	1134.787	264.245	131	53	10
Trihexosylceramide(d18:1/26:1)	1162.819	264.245	131	55	10
Trihexosylceramide(d18:1/22:0)	1108.772	264.245	131	52	10
GlcCer(d16:1/22:0)	756.635	236.213	136	44	10
GlcCer(d16:1/24:0)	784.666	236.213	136	45	10
GlcCer(d18:1/14:0)	672.541	264.245	136	42	10
GlcCer(d18:1/16:0)	700.572	264.245	136	43	10
GlcCer(d18:1/18:0)	728.604	264.245	136	44	10
GlcCer(d18:1/18:1)	726.588	264.245	136	44	10
GlcCer(d18:1/20:0)	756.635	264.245	136	44	10
GlcCer(d18:1/22:0)	784.666	264.245	136	45	10
GlcCer(d18:1/23:0)	798.682	264.245	136	45	10

GlcCer(d18:1/24:0)	812.697	264.245	136	46	10
GlcCer(d18:1/24:1)	810.682	264.245	136	46	10
GlcCer(d18:1/26:0)	840.729	264.245	136	47	10
GlcCer(d18:1/26:1)	838.713	264.245	136	47	10
GlcCer(d18:2/16:0)	698.557	262.229	136	43	10
GlcCer(d18:2/18:0)	726.588	262.229	136	44	10
GlcCer(d18:2/20:0)	754.619	262.229	136	44	10
GlcCer(d18:2/22:0)	782.65	262.229	136	45	10
GlcCer(d18:2/23:0)	796.666	262.229	136	45	10
GlcCer(d18:2/24:0)	810.682	262.229	136	46	10
Hexose	198.13	180.13	35	9	10
LysoPC a C14:0	468.3	184	79	30	15
LysoPC a C16:0	496.3	184	81	31	15
LysoPC a C16:1	494.3	184	79	30	15
LysoPC a C17:0	510.3	184	82	31	15
LysoPC a C18:0	524.3	184	85	31	15
LysoPC a C18:1	522.3	184	82	31	15
LysoPC a C18:2	520.3	184	82	31	15
LysoPC a C20:3	546.3	184	87	31	15
LysoPC a C20:4	544.3	184	85	31	15
LysoPC a C24:0	608.4	184	96	33	15
LysoPC a C26:0	636.5	184	101	34	15
LysoPC a C26:1	634.4	184	98	33	15
LysoPC a C28:0	664.5	184	105	35	15
LysoPC a C28:1	662.5	184	125	53	15
PC aa C24:0	622.4	184	98	33	15
PC aa C26:0	650.5	184	103	35	15
PC aa C28:1	676.5	184	106	35	15
PC aa C30:0	706.5	184	114	37	15
PC aa C32:0	734.6	184	119	39	15
PC aa C32:1	732.6	184	118	39	15

PC aa C32:2	730.5	184	117	38	15
PC aa C32:3	728.5	184	117	38	15
PC aa C34:1	760.6	184	125	41	15
PC aa C34:2	758.6	184	123	40	15
PC aa C34:3	756.6	184	123	40	15
PC aa C34:4	754.5	184	122	40	15
PC aa C36:0	790.6	184	131	43	15
PC aa C36:1	788.6	184	131	43	15
PC aa C36:2	786.6	184	130	42	15
PC aa C36:3	784.6	184	130	42	15
PC aa C36:4	782.6	184	129	42	15
PC aa C36:5	780.6	184	128	42	15
PC aa C36:6	778.5	184	128	42	15
PC aa C38:0	818.7	184	138	45	15
PC aa C38:1	816.7	184	138	45	15
PC aa C38:3	812.6	184	136	44	15
PC aa C38:4	810.6	184	136	44	15
PC aa C38:5	808.6	184	136	44	15
PC aa C38:6	806.6	184	135	44	15
PC aa C40:1	844.7	184	145	47	15
PC aa C40:2	842.7	184	144	47	15
PC aa C40:3	840.7	184	144	47	15
PC aa C40:4	838.6	184	144	47	15
PC aa C40:5	836.6	184	143	47	15
PC aa C40:6	834.6	184	143	46	15
PC aa C42:0	874.7	184	154	50	15
PC aa C42:1	872.7	184	153	50	15
PC aa C42:2	870.7	184	153	50	15
PC aa C42:4	866.7	184	152	50	15
PC aa C42:5	864.7	184	151	49	15
PC aa C42:6	862.6	184	150	49	15

PC ae C30:0	692.6	184	111	37	15
PC ae C30:1	690.5	184	109	36	15
PC ae C30:2	688.5	184	108	36	15
PC ae C32:1	718.6	184	115	38	15
PC ae C32:2	716.6	184	114	37	15
PC ae C34:0	748.6	184	122	40	15
PC ae C34:1	746.6	184	121	39	15
PC ae C34:2	744.6	184	120	39	15
PC ae C34:3	742.6	184	120	39	15
PC ae C36:0	776.7	184	128	42	15
PC ae C36:1	774.6	184	127	41	15
PC ae C36:2	772.6	184	127	41	15
PC ae C36:3	770.6	184	126	41	15
PC ae C36:4	768.6	184	126	41	15
PC ae C36:5	766.6	184	125	41	15
PC ae C38:0	804.7	184	135	44	15
PC ae C38:1	802.7	184	134	44	15
PC ae C38:2	800.7	184	133	43	15
PC ae C38:3	798.6	184	133	43	15
PC ae C38:4	796.6	184	133	43	15
PC ae C38:5	794.6	184	132	43	15
PC ae C38:6	792.6	184	132	43	15
PC ae C40:1	830.7	184	141	46	15
PC ae C40:2	828.7	184	141	46	15
PC ae C40:3	826.7	184	140	46	15
PC ae C40:4	824.7	184	140	45	15
PC ae C40:5	822.6	184	139	45	15
PC ae C40:6	820.6	184	139	45	15
PC ae C42:0	860.8	184	150	49	15
PC ae C42:1	858.7	184	141	46	15
PC ae C42:2	856.7	184	149	48	15

PC ae C42:3	854.7	184	148	48	15
PC ae C42:4	852.7	184	148	48	15
PC ae C42:5	850.7	184	147	48	15
PC ae C44:3	882.7	184	156	51	15
PC ae C44:4	880.7	184	156	51	15
PC ae C44:5	878.7	184	155	51	15
PC ae C44:6	876.7	184	154	50	15
SM C16:0	703.6	184	112	37	15
SM C16:1	701.6	184	111	37	15
SM C18:0	731.6	184	117	38	15
SM C18:1	729.6	184	117	38	15
SM C20:2	755.6	184	123	40	15
SM C24:0	815.7	184	137	45	15
SM C24:1	813.7	184	137	45	15
SM C26:0	843.7	184	145	47	15
SM C26:1	841.7	184	144	47	15
SMOH C14:1	689.6	184	108	36	15
SMOH C16:1	717.6	184	114	38	15
SMOH C22:1	801.7	184	134	43	15
SMOH C22:2	799.7	184	133	43	15
SMOH C24:1	829.7	184	141	46	15
TG(14:0/32:2)	792.708	547.472	126	31	10
TG(14:0/34:0)	824.77	579.535	131	33	10
TG(14:0/34:1)	822.755	577.519	131	33	10
TG(14:0/34:2)	820.739	575.503	131	33	10
TG(14:0/34:3)	818.723	573.488	131	33	10
TG(14:0/35:1)	836.77	591.535	131	34	10
TG(14:0/35:2)	834.755	589.519	131	34	10
TG(14:0/36:1)	850.786	605.55	144	35	10
TG(14:0/36:2)	848.77	603.535	144	35	10
TG(14:0/36:3)	846.755	601.519	144	35	10

TG(14:0/36:4)	844.739	599.503	144	35	10
TG(14:0/38:4)	872.77	627.535	144	35	10
TG(14:0/38:5)	870.755	625.519	144	35	10
TG(14:0/39:3)	888.801	643.566	144	36	10
TG(16:0/28:1)	766.692	493.425	126	30	10
TG(16:0/28:2)	764.676	491.409	126	30	10
TG(16:0/30:2)	792.708	519.441	126	31	10
TG(16:0/32:0)	824.77	551.503	131	33	10
TG(16:0/32:1)	822.755	549.488	131	33	10
TG(16:0/32:2)	820.739	547.472	131	33	10
TG(16:0/32:3)	818.723	545.456	131	33	10
TG(16:0/33:1)	836.77	563.503	131	34	10
TG(16:0/33:2)	834.755	561.488	131	34	10
TG(16:0/34:0)	852.801	579.535	161	35	10
TG(16:0/34:1)	850.786	577.519	161	33	10
TG(16:0/34:2)	848.77	575.503	126	35	10
TG(16:0/34:3)	846.755	573.488	144	35	10
TG(16:0/34:4)	844.739	571.472	144	35	10
TG(16:0/35:1)	864.801	591.535	144	35	10
TG(16:0/35:2)	862.786	589.519	144	35	10
TG(16:0/35:3)	860.77	587.503	144	35	10
TG(16:0/36:2)	876.801	603.535	144	35	10
TG(16:0/36:3)	874.786	601.519	144	35	10
TG(16:0/36:4)	872.77	599.503	144	35	10
TG(16:0/36:5)	870.755	597.488	144	35	10
TG(16:0/36:6)	868.739	595.472	144	35	10
TG(16:0/37:3)	888.801	615.535	144	36	10
TG(16:0/38:1)	906.848	633.582	161	37	10
TG(16:0/38:2)	904.833	631.566	161	37	10
TG(16:0/38:3)	902.817	629.55	161	37	10
TG(16:0/38:4)	900.801	627.535	161	37	10

TG(16:0/38:5)	898.786	625.519	161	37	10
TG(16:0/38:6)	896.77	623.503	161	37	10
TG(16:0/38:7)	894.755	621.488	161	37	10
TG(16:0/40:6)	924.801	651.535	161	39	10
TG(16:0/40:7)	922.786	649.519	161	39	10
TG(16:0/40:8)	920.77	647.503	161	39	10
TG(16:1/28:0)	766.692	495.441	126	30	10
TG(16:1/30:1)	792.708	521.456	126	31	10
TG(16:1/32:0)	822.755	551.503	131	33	10
TG(16:1/32:1)	820.739	549.488	131	33	10
TG(16:1/32:2)	818.723	547.472	131	33	10
TG(16:1/33:1)	834.755	563.503	131	34	10
TG(16:1/34:0)	850.786	579.535	144	35	10
TG(16:1/34:1)	848.77	577.519	144	35	10
TG(16:1/34:2)	846.755	575.503	144	35	10
TG(16:1/34:3)	844.739	573.488	144	35	10
TG(16:1/36:1)	876.801	605.55	144	35	10
TG(16:1/36:2)	874.786	603.535	144	35	10
TG(16:1/36:3)	872.77	601.519	144	35	10
TG(16:1/36:4)	870.755	599.503	144	35	10
TG(16:1/36:5)	868.739	597.488	144	35	10
TG(16:1/38:3)	900.801	629.55	161	37	10
TG(16:1/38:4)	898.786	627.535	161	37	10
TG(16:1/38:5)	896.77	625.519	161	37	10
TG(17:0/32:1)	836.77	549.488	131	34	10
TG(17:0/34:1)	864.801	577.519	144	35	10
TG(17:0/34:2)	862.786	575.503	144	35	10
TG(17:0/34:3)	860.77	573.488	144	35	10
TG(17:0/36:3)	888.801	601.519	144	36	10
TG(17:0/36:4)	886.786	599.503	144	36	10
TG(17:1/32:1)	834.755	549.488	131	34	10

TG(17:1/34:1)	862.786	577.519	144	35	10
TG(17:1/34:2)	860.77	575.503	144	35	10
TG(17:1/34:3)	858.755	573.488	144	35	10
TG(17:1/36:3)	886.786	601.519	144	36	10
TG(17:1/36:4)	884.77	599.503	144	36	10
TG(17:1/36:5)	882.755	597.488	144	36	10
TG(17:1/38:5)	910.786	625.519	161	38	10
TG(17:1/38:6)	908.77	623.503	161	38	10
TG(17:1/38:7)	906.755	621.488	161	38	10
TG(17:2/34:2)	858.755	575.503	144	35	10
TG(17:2/34:3)	856.739	573.488	144	35	10
TG(17:2/36:2)	886.786	603.535	144	36	10
TG(17:2/36:3)	884.77	601.519	144	36	10
TG(17:2/36:4)	882.755	599.503	144	36	10
TG(17:2/38:5)	908.77	625.519	161	38	10
TG(17:2/38:6)	906.755	623.503	161	38	10
TG(17:2/38:7)	904.739	621.488	161	38	10
TG(18:0/30:0)	824.77	523.472	131	33	10
TG(18:0/30:1)	822.755	521.456	131	33	10
TG(18:0/32:0)	852.801	551.503	144	35	10
TG(18:0/32:1)	850.786	549.488	144	35	10
TG(18:0/32:2)	848.77	547.472	144	35	10
TG(18:0/34:2)	876.801	575.503	144	35	10
TG(18:0/34:3)	874.786	573.488	144	35	10
TG(18:0/36:1)	906.848	605.55	116	37	10
TG(18:0/36:2)	904.833	603.535	161	37	10
TG(18:0/36:3)	902.817	601.519	161	37	10
TG(18:0/36:4)	900.801	599.503	161	37	10
TG(18:0/36:5)	898.786	597.488	161	37	10
TG(18:0/38:6)	924.801	623.503	161	39	10
TG(18:0/38:7)	922.786	621.488	161	39	10

TG(18:1/26:0)	766.692	467.409	126	30	10
TG(18:1/28:1)	792.708	493.425	126	31	10
TG(18:1/30:0)	822.755	523.472	131	33	10
TG(18:1/30:1)	820.739	521.456	131	33	10
TG(18:1/30:2)	818.723	519.441	131	33	10
TG(18:1/31:0)	836.77	537.488	131	34	10
TG(18:1/32:0)	850.786	551.503	144	35	10
TG(18:1/32:1)	848.77	549.488	144	35	10
TG(18:1/32:2)	846.755	547.472	144	35	10
TG(18:1/32:3)	844.739	545.456	144	35	10
TG(18:1/33:0)	864.801	565.519	144	35	10
TG(18:1/33:1)	862.786	563.503	144	35	10
TG(18:1/33:2)	860.77	561.488	144	35	10
TG(18:1/33:3)	858.755	559.472	144	35	10
TG(18:1/34:1)	876.801	577.519	144	35	10
TG(18:1/34:2)	874.786	575.503	144	35	10
TG(18:1/34:3)	872.77	573.488	144	35	10
TG(18:1/34:4)	870.755	571.472	144	35	10
TG(18:1/35:2)	888.801	589.519	144	36	10
TG(18:1/35:3)	886.786	587.503	144	36	10
TG(18:1/36:0)	906.848	607.566	161	37	10
TG(18:1/36:1)	904.833	605.55	161	37	10
TG(18:1/36:2)	902.817	603.535	46	37	10
TG(18:1/36:3)	900.801	601.519	161	37	10
TG(18:1/36:4)	898.786	599.503	161	37	10
TG(18:1/36:5)	896.77	597.488	161	37	10
TG(18:1/36:6)	894.755	595.472	161	37	10
TG(18:1/38:5)	924.801	625.519	161	39	10
TG(18:1/38:6)	922.786	623.503	161	39	10
TG(18:1/38:7)	920.77	621.488	161	39	10
TG(18:2/28:0)	792.708	495.441	126	31	10

TG(18:2/30:0)	820.739	523.472	131	33	10
TG(18:2/30:1)	818.723	521.456	131	33	10
TG(18:2/31:0)	834.755	537.488	131	34	10
TG(18:2/32:0)	848.77	551.503	144	35	10
TG(18:2/32:1)	846.755	549.488	144	35	10
TG(18:2/32:2)	844.739	547.472	144	35	10
TG(18:2/33:0)	862.786	565.519	144	35	10
TG(18:2/33:1)	860.77	563.503	144	35	10
TG(18:2/33:2)	858.755	561.488	144	35	10
TG(18:2/34:0)	876.801	579.535	144	35	10
TG(18:2/34:1)	874.786	577.519	144	35	10
TG(18:2/34:2)	872.77	575.503	144	35	10
TG(18:2/34:3)	870.755	573.488	144	35	10
TG(18:2/34:4)	868.739	571.472	144	35	10
TG(18:2/35:1)	888.801	591.535	144	36	10
TG(18:2/35:2)	886.786	589.519	144	36	10
TG(18:2/35:3)	884.77	587.503	144	36	10
TG(18:2/36:0)	904.833	607.566	161	37	10
TG(18:2/36:1)	902.817	605.55	161	37	10
TG(18:2/36:2)	900.801	603.535	161	37	10
TG(18:2/36:3)	898.786	601.519	161	37	10
TG(18:2/36:4)	896.77	599.503	161	37	10
TG(18:2/36:5)	894.755	597.488	161	37	10
TG(18:2/38:4)	924.801	627.535	161	39	10
TG(18:2/38:5)	922.786	625.519	161	39	10
TG(18:2/38:6)	920.77	623.503	161	39	10
TG(18:3/30:0)	818.723	523.472	131	33	10
TG(18:3/32:0)	846.755	551.503	144	35	10
TG(18:3/32:1)	844.739	549.488	144	35	10
TG(18:3/33:2)	856.739	561.488	144	35	10
TG(18:3/34:0)	874.786	579.535	144	35	10

TG(18:3/34:1)	872.77	577.519	144	35	10
TG(18:3/34:2)	870.755	575.503	144	35	10
TG(18:3/34:3)	868.739	573.488	144	35	10
TG(18:3/35:2)	884.77	589.519	144	36	10
TG(18:3/36:1)	900.801	605.55	161	37	10
TG(18:3/36:2)	898.786	603.535	161	37	10
TG(18:3/36:3)	896.77	601.519	161	37	10
TG(18:3/36:4)	894.755	599.503	161	37	10
TG(18:3/38:5)	920.77	625.519	161	39	10
TG(18:3/38:6)	918.755	623.503	161	39	10
TG(20:0/32:3)	874.786	545.456	144	35	10
TG(20:0/32:4)	872.77	543.441	144	35	10
TG(20:0/34:1)	906.848	577.519	161	37	10
TG(20:1/24:3)	760.645	433.331	126	30	10
TG(20:1/26:1)	792.708	465.394	126	31	10
TG(20:1/30:1)	848.77	521.456	144	35	10
TG(20:1/31:0)	864.801	537.488	144	35	10
TG(20:1/32:1)	876.801	549.488	144	35	10
TG(20:1/32:2)	874.786	547.472	144	35	10
TG(20:1/32:3)	872.77	545.456	144	35	10
TG(20:1/34:0)	906.848	579.535	161	37	10
TG(20:1/34:1)	904.833	577.519	161	37	10
TG(20:1/34:2)	902.817	575.503	161	37	10
TG(20:1/34:3)	900.801	573.488	161	37	10
TG(20:2/32:0)	876.801	551.503	144	35	10
TG(20:2/32:1)	874.786	549.488	144	35	10
TG(20:2/34:1)	902.817	577.519	161	37	10
TG(20:2/34:2)	900.801	575.503	161	37	10
TG(20:2/34:3)	898.786	573.488	161	37	10
TG(20:2/34:4)	896.77	571.472	161	37	10
TG(20:2/36:5)	922.786	597.488	161	39	10

TG(20:3/32:0)	874.786	551.503	144	35	10
TG(20:3/32:1)	872.77	549.488	144	35	10
TG(20:3/32:2)	870.755	547.472	144	35	10
TG(20:3/34:0)	902.817	579.535	161	37	10
TG(20:3/34:1)	900.801	577.519	161	37	10
TG(20:3/34:2)	898.786	575.503	161	37	10
TG(20:3/34:3)	896.77	573.488	161	37	10
TG(20:3/36:3)	924.801	601.519	161	39	10
TG(20:3/36:4)	922.786	599.503	161	39	10
TG(20:3/36:5)	920.77	597.488	161	39	10
TG(20:4/30:0)	844.739	523.472	144	35	10
TG(20:4/32:0)	872.77	551.503	144	35	10
TG(20:4/32:1)	870.755	549.488	144	35	10
TG(20:4/32:2)	868.739	547.472	144	35	10
TG(20:4/33:2)	882.755	561.488	144	36	10
TG(20:4/34:0)	900.801	579.535	161	37	10
TG(20:4/34:1)	898.786	577.519	161	37	10
TG(20:4/34:2)	896.77	575.503	161	37	10
TG(20:4/34:3)	894.755	573.488	161	37	10
TG(20:4/35:3)	908.77	587.503	161	38	10
TG(20:4/36:2)	924.801	603.535	161	39	10
TG(20:4/36:3)	922.786	601.519	161	39	10
TG(20:4/36:4)	920.77	599.503	161	39	10
TG(20:4/36:5)	918.755	597.488	161	39	10
TG(20:5/34:0)	898.786	579.535	161	37	10
TG(20:5/34:1)	896.77	577.519	161	37	10
TG(20:5/34:2)	894.755	575.503	161	37	10
TG(20:5/36:2)	922.786	603.535	161	39	10
TG(20:5/36:3)	920.77	601.519	161	39	10
TG(22:0/32:4)	900.801	543.441	161	37	10
TG(22:1/32:5)	896.77	541.425	161	37	10

TG(22:2/32:4)	896.77	543.441	161	37	10
TG(22:3/30:2)	870.755	519.441	144	35	10
TG(22:4/32:0)	900.801	551.503	161	37	10
TG(22:4/32:2)	896.77	547.472	161	37	10
TG(22:4/34:2)	924.801	575.503	161	39	10
TG(22:5/32:0)	898.786	551.503	161	37	10
TG(22:5/32:1)	896.77	549.488	161	37	10
TG(22:5/34:1)	924.801	577.519	161	39	10
TG(22:5/34:2)	922.786	575.503	161	39	10
TG(22:5/34:3)	920.77	573.488	161	39	10
TG(22:6/32:0)	896.77	551.503	161	37	10
TG(22:6/32:1)	894.755	549.488	161	37	10
TG(22:6/34:1)	922.786	577.519	161	39	10
TG(22:6/34:2)	920.77	575.503	161	39	10
TG(22:6/34:3)	918.755	573.488	161	39	10

Supplementary Table S3. Calibration Regression, LOD and LOQ

Analyte	Correlation Coefficient (R^2)	LOD (µM)	LOQ (µM)
1,3-Diaminopropane	0.9976	0.0278	0.0926
1-Methylnicotinamide	0.9983	0.125	0.417
3-Methoxytyramine	0.9997	0.00619	0.0206
3-Nitrotyrosine	0.9952	0.0595	0.198
5-Methoxytryptamine	0.9925	0.0136	0.0452
5-Methyluridine	0.9974	0.0750	0.250
7-Methylguanine	0.9989	0.0708	0.236
Adenine	0.9990	0.0120	0.0400
Adenosine	0.9997	0.00382	0.0127
Agmatine	0.9995	0.00552	0.0184
Alanine	0.9950	0.176	0.587
Allantoin	0.9993	0.409	1.36
alpha-Aminoadipic acid	0.9984	0.0750	0.250
alpha-Aminobutyric acid	0.9922	0.0209	0.0696
Arginine	0.9956	0.137	0.457
Asparagine	0.9995	0.102	0.340
Aspartic acid	0.9989	0.303	1.01
Asymmetric dimethylarginine	0.9935	0.0349	0.116
beta-Alanine	0.9987	0.0423	0.141
Betaine	0.9994	0.481	1.60
Cadaverine	0.9961	0.0112	0.0373
Carnosine	0.9993	0.0155	0.0517
Choline	0.9990	0.178	0.593
cis-4-Hydroxyproline	0.9993	0.0124	0.0415
Citrulline	0.9997	0.0756	0.252
Creatine	0.9993	0.213	0.710
Creatinine	0.9995	0.327	1.09

Cystathionine	0.9987	0.0518	0.173
Cytidine	0.9978	0.0361	0.120
Cytosine	0.9985	0.0636	0.212
Deoxyadenosine	0.9992	0.0037	0.0122
Deoxycytidine	0.9996	0.0174	0.0581
Deoxyguanosine	0.9992	0.00581	0.0194
Deoxyinosine	0.9997	0.00326	0.0109
Deoxyuridine	0.9993	0.219	0.730
Dimethylamine	0.9985	0.00785	0.0262
DOPA	0.9987	0.0243	0.0810
Dopamine	0.9993	0.0150	0.0501
Epinephrine	0.9997	0.0100	0.0334
Ethanolamine	0.9975	0.0432	0.144
gamma-Aminobutyric acid	0.9976	0.00711	0.0237
Glutamic acid	0.9983	0.129	0.428
Glutamine	0.9995	0.129	0.430
Glycine	0.9988	0.390	1.30
Guanine	0.9998	0.00721	0.0240
Guanosine	0.9987	0.00279	0.00929
Histamine	0.9993	0.0115	0.0382
Histidine	0.9977	0.0555	0.185
Homoarginine	0.9989	0.00653	0.0218
Homocitrulline	0.9995	0.0564	0.188
Hypoxanthine	0.9993	0.00787	0.0262
Indole	0.9993	0.647	2.16
Indole-3-acetamide	0.9909	0.0219	0.0731
Inosine	0.9948	0.0203	0.0678
Isoleucine	0.9996	0.361	1.20
Kynurenine	0.9998	0.0237	0.0789
Leucine	0.9992	0.255	0.850
Lysine	0.9976	0.404	1.35

Methionine	0.9986	0.111	0.369
Methionine sulfoxide	0.9996	0.132	0.439
Methylamine	0.9908	0.121	0.403
Methylhistidine	0.9984	0.0938	0.313
N1,N12-Diacetylspermine	0.9981	0.0676	0.225
N1-Acetylspermidine	0.9985	0.0207	0.0690
N2-Acetyl-Ornithine	0.9979	0.0920	0.307
N-Acetylputrescine	0.9973	0.0144	0.0481
Nicotinamide ribotide	0.9991	0.332	1.11
Norepinephrine	0.9992	0.0987	0.329
Nudifloramide	0.9987	0.358	1.19
Ornithine	0.9934	0.0588	0.196
Phenylalanine	0.9933	0.0445	0.148
Phenylethylamine	0.9998	0.0192	0.0640
Proline	0.9932	0.694	2.31
Putrescine	0.9977	0.0667	0.222
Sarcosine	0.9996	0.0298	0.0992
Serine	0.9967	0.154	0.515
Serotonin	0.9987	0.0236	0.0787
Spermidine	0.9998	0.0219	0.0729
Spermine	0.9994	0.0441	0.147
Taurine	0.9994	0.256	0.853
Threonine	0.9989	0.670	2.23
Thymidine	0.9996	0.0326	0.109
Thymine	0.9980	0.0592	0.197
Total dimethylarginine	0.9928	0.0090	0.0299
trans-4-Hydroxyproline	0.9999	0.0906	0.302
Trimethylamine	0.9989	0.0828	0.276
Trimethylamine N-Oxide	0.9958	0.0973	0.324
Tryptamine	0.9990	0.0014	0.00464
Tryptophan	0.9998	0.0340	0.113

Tyramine	0.9952	0.0259	0.0862
Tyrosine	0.9995	0.1807	0.602
Uracil	0.9981	9.52	31.7
Urea	0.9993	102	341
Uridine	0.9993	0.308	1.03
Valine	0.9989	0.295	0.982
2,5-Furandicarboxylic acid	0.9998	0.0124	0.0415
2-Hydroxy-2-methylbutyric acid	0.9977	0.213	0.710
2-Hydroxy-3-methylvaleric acid	0.9959	0.0176	0.0587
2-Hydroxybutyric acid	0.9993	0.115	0.384
2-Hydroxyglutaric acid	0.9978	0.0100	0.0334
2-Hydroxyisobutyric acid	0.9926	0.0223	0.0744
2-Hydroxyisovaleric acid	0.9991	0.0100	0.0334
2-Hydroxyphenylacetic acid	0.9937	0.0426	0.142
2-Oxoadipic acid	0.9995	1.34	4.46
2-Oxoisocaproic acid	0.9985	0.0570	0.190
3-(3-Hydroxyphenyl)-3-hydroxypropanoic acid	0.9986	0.00195	0.00648
3,4-Dihydroxybutyric acid	0.9996	0.0984	0.328
3-Aminoisobutyric acid	0.9989	0.240	0.800
3-Carboxy-4-methyl-5-propyl-2-furanpropionic acid	0.9997	0.0260	0.0866
3-Deoxyglucosone	0.9972	0.0110	0.0368
3-Hydroxybutyric acid	0.9975	0.224	0.745
3-Hydroxyisobutyric acid	0.9990	0.250	0.833
3-Hydroxyisovaleric acid	0.9998	0.0879	0.293
3-Hydroxyphenylacetic acid	0.9982	0.203	0.676
3-Indoleacetic acid	0.9998	0.0568	0.189
3-Methyladipic acid	0.9985	0.00801	0.0267
4-Ethylphenyl sulfate	0.9993	0.144	0.478
4-Hydroxybenzoic acid	0.9966	0.0808	0.269
4-Hydroxyhippuric acid	0.9990	0.0349	0.116

4-Hydroxyphenylacetic acid	0.9984	0.100	0.333
4-Hydroxyphenylpyruvic acid	0.9966	0.283	0.943
5-Aminolevulinic Acid	0.9970	0.320	1.07
5-Hydroxyindoleacetic acid	0.9960	0.0133	0.0444
5-Oxoproline	0.9920	0.135	0.448
Acetoacetic acid	0.9965	0.130	0.432
alpha-Ketoglutaric acid	0.9949	0.0193	0.0644
alpha-Ketoisovaleric acid	0.9968	0.0565	0.188
Argininic acid	0.9976	0.199	0.663
Benzoic acid	0.9958	0.142	0.472
Butyric acid + Isobutyric acid	0.9994	0.0835	0.278
Caffeic acid	0.9986	0.843	2.81
Caproic acid	0.9982	0.273	0.911
Caprylic acid	0.9969	0.133	0.443
cis-Aconitic acid	0.9980	0.0162	0.0541
Citric acid	0.9988	0.7043	2.35
Cyclic AMP	0.9989	0.00898	0.0299
Dimethylglycine	0.9979	0.158	0.525
Ethylmalonic acid	0.9996	0.0507	0.169
Fumaric acid	0.9966	0.0236	0.0786
Glutaric acid	0.9933	0.00477	0.0159
Glyceric acid	0.9996	0.00841	0.0280
Guanidinopropionic acid	0.9981	0.105	0.350
Guanidoacetic acid	0.9959	0.135	0.450
Hippuric acid	0.9969	0.0327	0.109
Homovanillic acid	0.9982	0.0521	0.174
Indole-3-carboxylic acid	0.9981	0.0698	0.233
Indole-3-propionic acid	0.9986	0.00880	0.0293
Indolelactic acid	0.9990	0.0125	0.0417
Indoxyl glucoside	0.9982	0.323	1.08
Indoxyl glucuronide	0.9985	0.0375	0.125

Indoxyl sulfate	0.9956	0.220	0.732
Isocitric acid	0.9996	1.54	5.13
Isovaleric acid	0.9997	0.0611	0.204
Kynurenic acid	0.9947	0.00700	0.0233
Lactic acid	0.9992	2.00	6.66
Maleic acid	0.9974	0.125	0.417
Malic acid	0.9986	0.0225	0.0750
Malonic acid	0.9955	0.0182	0.0608
Methylmalonic acid	0.9997	0.0125	0.0417
N1-Acetyl-Lysine	0.9997	0.0600	0.200
N6-Acetyl-Lysine	0.9968	0.0625	0.208
N-Acetyl-Alanine	0.9983	0.0315	0.105
N-Acetyl-Arginine	0.9946	0.341	1.14
N-Acetyl-Asparagine	0.9984	0.0500	0.167
N-Acetyl-Aspartic acid	0.9978	0.00750	0.0250
N-Acetyl-Glutamic acid	0.9999	0.0150	0.0500
N-Acetyl-Glutamine	0.9930	0.221	0.735
N-Acetyl-Glycine	0.9997	0.0605	0.202
N-Acetyl-Histidine	0.9995	0.385	1.28
N-Acetyl-Isoleucine	0.9975	0.0200	0.0667
N-Acetyl-Leucine	0.9909	0.0798	0.266
N-Acetyl-Methionine	0.9939	0.0189	0.0631
N-Acetyl-Proline	0.9982	0.0131	0.0437
N-Acetyl-Serine	0.9957	0.0286	0.0954
N-Acetyl-Tryptophan	0.9977	0.0500	0.167
N-Acetyl-Tyrosine	0.9987	0.0694	0.231
N-Acetyl-Valine	0.9959	0.0347	0.116
N-Methyl-Aspartic acid	0.9920	0.357	1.19
Orotic acid	0.9992	0.0570	0.190
p-Cresol sulfate	0.9976	0.435	1.45
Phenylacetic acid	0.9984	0.0232	0.0774

Phenylacetylglutamine	0.9982	0.0125	0.0417
Picolinic acid	0.9995	0.0719	0.240
Pipecolic acid	0.9974	0.0527	0.176
Propionic acid	0.9992	0.0573	0.191
Pyruvic acid	0.9994	0.195	0.649
Quinaldic acid	0.9946	0.120	0.400
Quinoline-4-carboxylic acid	0.9995	0.00995	0.0332
Quinolinic acid	0.9984	0.00987	0.0329
Salicylic acid	0.9992	0.0968	0.323
Shikimic acid	0.9981	0.138	0.460
Succinic acid	0.9997	0.167	0.556
Tartaric acid	0.9977	0.0375	0.125
Threonic acid	0.9969	0.584	1.95
Tiglylglycine	0.9970	0.00300	0.0100
Uric acid	0.9975	2.93	9.76
Valeric acid	0.9997	0.112	0.372
Xanthine	0.9998	0.417	1.39
Xanthosine	0.9993	0.366	1.22

Supplementary Table S4. Intra- and Inter-day Accuracy and Precision

Analyte	Low				Mid				High			
	Intra-day		Inter-day		Intra-day		Inter-day		Intra-day		Inter-day	
	Accuracy (%)	CV (%)	Accuracy (%)	CV (%)	Accuracy (%)	CV (%)	Accuracy (%)	CV (%)	Accuracy (%)	CV (%)	Accuracy (%)	CV (%)
1,3-Diaminopropane	104	5.56	110	6.35	106	0.891	105	2.64	116	3.14	113	5.16
1-Methylnicotinamide	97.8	4.55	102	4.55	93.5	1.49	102	7.97	113	2.27	103	9.43
3-Methoxytyramine	99.8	1.17	101	6.57	98.4	2.50	101	4.07	100	1.87	98.6	4.02
3-Nitrotyrosine	103	4.57	108	7.60	107	0.292	107	5.91	110	0.363	109	6.49
5-Methoxytryptamine	94.0	1.70	92.4	9.90	94.8	1.26	93.6	4.40	101	4.19	97.2	11.7
5-Methyluridine	97.4	2.85	94.7	10.9	93.6	5.25	98.8	7.14	99.0	3.57	105	5.98
7-Methylguanine	105	5.22	107	6.37	108	1.86	98.1	4.60	108	3.66	105	5.85
Adenine	104	5.31	110	5.38	111	1.92	113	5.31	118	0.537	118	3.64
Adenosine	110	3.67	106	4.54	109	1.82	107	2.82	118	0.824	109	8.56
Agmatine	93.6	4.11	97.6	6.78	98.2	0.954	99.1	7.67	94.2	3.13	91.4	6.72
Alanine	96.3	0.106	96.2	4.00	97.1	0.208	96.9	2.50	101	2.01	98.9	10.0
Allantoin	115	8.27	115	8.80	99.8	5.17	101	6.98	97.7	8.16	101	8.93
alpha-Aminoadipic acid	94.1	5.98	88.8	15.2	96.5	0.479	97.0	2.86	85.4	2.47	87.6	6.20
alpha-Aminobutyric acid	88.2	4.39	92.3	8.89	87.6	2.59	90.5	3.17	91.3	1.06	92.2	10.4
Arginine	91.8	2.58	94.2	7.51	92.5	2.06	94.5	5.77	101	2.85	98.0	10.1
Asparagine	97.2	0.234	97.5	11.5	102	0.950	101	5.36	107	3.28	104	8.88
Aspartic acid	99.0	0.720	99.8	18.8	105	1.20	103	12.6	108	4.87	98.3	9.73
Asymmetric dimethylarginine	99.3	4.54	104	5.48	98.4	2.77	101	5.77	95.8	0.147	95.7	7.16
beta-Alanine	93.4	3.54	98.1	4.88	84.1	2.41	86.2	4.56	97.6	5.95	92.1	13.8
Betaine	105	3.41	102	4.60	116	3.09	108	7.48	105	0.553	106	7.33
Cadaverine	104	8.64	90.5	13.2	101	6.82	94.1	9.38	105	17.9	95.2	19.7
Carnosine	104	7.31	92.4	7.52	108	3.40	98.1	6.60	105	5.28	100	5.90
Choline	113	3.42	107	5.00	111	2.01	113	5.40	120	1.53	117	2.73

cis-4-Hydroxyproline	94.2	1.18	95.3	3.13	90.8	0.776	90.1	4.35	95.4	2.16	92.4	3.21
Citrulline	102	2.18	104	9.01	112	5.10	106	7.15	116	7.52	107	7.95
Creatine	92.7	4.91	97.5	5.92	105	0.518	105	2.31	110	3.66	106	5.21
Creatinine	104	2.26	106	3.72	106	4.02	111	5.72	105	0.223	105	4.60
Cystathionine	114	9.11	119	14.7	113	11.7	119	15.8	108	3.31	117	9.09
Cytidine	105	2.01	103	5.96	111	3.41	107	6.38	108	1.14	109	4.01
Cytosine	119	3.27	115	5.91	112	0.739	112	5.13	104	0.216	105	2.37
Deoxyadenosine	109	0.416	108	5.17	117	0.256	117	2.51	119	0.973	118	1.97
Deoxycytidine	108	1.55	103	4.51	112	1.78	106	5.45	105	1.12	106	5.01
Deoxyguanosine	99.7	8.74	91.7	12.4	99.1	5.16	94.3	6.45	104	2.37	98.1	5.81
Deoxyinosine	111	0.187	111	2.66	106	1.11	107	7.88	104	1.07	105	8.02
Deoxyuridine	94.5	7.07	102	8.88	94.6	2.41	103	2.42	111	1.44	110	6.00
Dimethylamine	102	4.90	109	5.02	104	5.56	113	11.1	107	4.84	115	6.70
DOPA	98.3	2.19	96.2	4.19	102	4.32	97.4	6.57	106	4.64	102	5.80
Dopamine	99.3	4.05	105	5.64	108	2.51	112	3.12	110	0.250	110	8.27
Epinephrine	114	1.00	111	2.39	105	0.938	104	7.21	94.0	2.48	96.4	9.82
Ethanolamine	106	4.99	96.1	10.3	102	4.62	95.7	6.11	94.7	4.69	90.1	5.03
gamma-Aminobutyric acid	104	2.97	106	7.83	109	3.32	95.2	11.6	95.3	0.970	92.8	4.81
Glutamic acid	97.2	1.75	94.6	6.23	98.7	0.441	97.5	4.31	110	4.41	101	7.04
Glutamine	92.9	1.69	98.8	12.8	97.9	1.75	100	4.05	106	7.33	102	7.52
Glycine	98.9	1.59	93.7	1.72	93.6	0.729	94.2	4.02	107	3.80	101	6.76
Guanine	97.2	1.91	99.1	9.93	107	4.50	101	5.68	105	3.43	101	4.05
Guanosine	93.2	0.337	92.9	5.39	98.9	5.85	88.9	11.2	98.9	4.36	92.3	7.10
Histamine	94.1	6.35	100	7.20	90.7	4.74	95.2	5.90	94.8	6.29	89.2	6.33
Histidine	97.8	1.58	99.4	6.91	91.6	2.87	96.2	4.80	98.7	0.571	99.3	3.58
Homoarginine	98.2	4.76	93.7	4.81	97.9	1.91	99.9	2.03	113	1.58	115	10.1
Homocitrulline	104	1.85	102	5.02	92.5	1.46	93.9	8.70	96.0	2.50	93.6	2.80
Hypoxanthine	94.4	2.58	103	5.49	92.7	3.43	96.8	4.66	101	4.20	94.6	6.99
Indole	87.2	6.43	102	15.2	87.9	5.97	101	7.64	92.4	4.39	89.6	6.86
Indole-3-acetamide	95.4	4.47	102	6.75	96.0	7.11	103	9.67	97.0	0.885	96.2	4.97
Inosine	91.3	1.86	89.6	5.52	95.4	0.742	96.1	4.10	104	3.14	99.5	4.26

Isoleucine	91.4	0.713	92.0	5.32	96.8	0.210	97.0	5.26	106	2.90	103	4.50
Kynurenine	98.9	2.96	96.1	4.49	96.6	2.61	94.1	6.49	101	2.26	99.2	3.77
Leucine	90.5	3.34	93.6	9.75	94.2	1.46	95.6	2.80	115	6.65	107	7.50
Lysine	91.4	2.08	93.4	4.57	94.4	0.0703	94.4	2.64	103	5.20	97.9	11.7
Methionine	108	3.47	104	7.43	101	0.351	100	2.27	108	1.69	106	1.79
Methionine sulfoxide	108	2.34	106	15.8	103	1.38	96.7	6.92	116	12.0	113	13.1
Methylamine	92.6	4.16	103	8.01	96.5	3.93	108	10.2	96.8	1.46	105	3.10
Methylhistidine	95.5	2.79	98.2	10.4	91.5	3.82	95.1	5.38	98.8	3.50	95.5	7.66
N1,N12-Diacetylspermine	93.9	6.27	103	9.07	110	4.60	105	5.02	108	7.16	99.4	8.56
N1-Acetylspermidine	97.2	2.76	100	4.98	104	1.95	102	8.85	102	0.756	103	9.91
N2-Acetyl-Ornithine	88.1	3.92	91.7	14.3	93.6	0.638	93.0	6.97	109	4.46	101	8.29
N-Acetylputrescine	95.0	2.55	97.5	8.11	99.7	3.22	94.8	5.22	99.6	3.45	91.8	8.50
Nicotinamide ribotide	102	10.9	113	13.4	95.8	7.18	102	9.70	106	4.68	108	6.89
Norepinephrine	108	2.75	111	5.05	107	1.86	109	2.65	98.2	5.00	103	14.9
Nudifloramide	88.6	3.15	82.4	4.68	87.4	1.99	90.6	5.74	88.7	2.04	95.1	5.88
Ornithine	101	2.63	98.7	15.6	96.7	0.365	96.3	3.55	115	8.48	106	14.1
Phenylalanine	96.2	1.93	98.1	4.28	94.5	0.299	94.2	1.99	93.3	0.694	94.0	6.93
Phenylethylamine	98.9	2.88	102	7.90	111	1.50	113	8.22	117	3.96	117	11.1
Proline	96.1	3.58	101	4.78	93.8	4.80	102	7.90	105	1.17	104	5.59
Putrescine	89.7	0.220	89.5	5.50	89.0	5.59	94.2	6.82	103	5.83	97.4	12.5
Sarcosine	98.6	4.25	103	17.6	89.7	3.38	92.9	8.65	89.9	1.24	91.0	10.8
Serine	97.9	2.73	101	6.20	96.7	2.87	99.5	11.5	104	0.215	104	2.55
Serotonin	95.5	1.42	96.9	9.99	103	3.02	99.5	6.50	106	3.99	102	7.24
Spermidine	104	2.40	101	10.3	101	2.51	103	4.53	102	0.386	101	15.1
Spermine	99.9	5.09	105	5.61	101	2.26	103	3.60	99.8	0.204	99.6	4.20
Taurine	100	0.398	101	4.78	98.9	0.0980	98.8	7.39	98.7	1.31	97.4	10.8
Threonine	90.1	3.29	87.3	7.50	87.0	0.866	88.7	1.87	98.9	3.01	96.0	5.97
Thymidine	102	2.19	105	8.53	102	5.33	108	10.6	102	4.77	108	5.33
Thymine	90.4	4.21	86.7	6.76	91.1	4.78	85.9	6.12	88.8	2.55	86.6	2.55
Total dimethylarginine	101	2.12	91.8	3.50	102	0.970	111	3.87	102	1.11	96.3	3.64
trans-4-Hydroxyproline	97.7	2.85	102	4.20	99.1	0.328	98.8	2.66	104	3.45	98.3	5.45

Trimethylamine	100	3.55	111	8.51	101	1.97	95.2	5.37	105	4.33	98.8	4.59
Trimethylamine N-Oxide	86.9	0.675	87.5	5.16	89.3	1.61	85.3	4.75	88.8	4.44	85.1	5.14
Tryptamine	87.9	2.01	96.0	8.39	94.5	3.38	98.6	4.13	105	1.22	103	6.49
Tryptophan	102	2.43	99.8	3.07	102	1.49	101	4.51	111	4.79	104	6.83
Tyramine	99.2	0.35	99.5	6.18	96.3	1.81	98.0	3.19	101	3.42	98.1	5.65
Tyrosine	102	5.17	96.5	6.17	98.4	4.53	94.2	5.87	102	2.00	94.3	7.99
Uracil	92.3	2.94	92.6	5.23	91.2	2.66	92.9	6.13	90.3	1.38	98.9	4.23
Urea	85.8	7.84	96.7	11.3	94.2	4.28	102	11.5	94.1	3.50	101	6.52
Uridine	86.2	2.81	93.3	7.52	92.9	6.79	99.7	6.98	104	4.21	108	13.4
Valine	97.5	2.53	100	2.54	99.7	1.60	101	2.76	113	3.92	108	4.53
2,5-Furandicarboxylic acid	96.6	0.368	96.3	0.600	96.7	2.05	93.0	3.97	101	1.86	103	2.20
2-Hydroxy-2-methylbutyric acid	85.9	8.91	95.6	10.2	94.0	1.26	92.9	3.13	101	1.30	99.9	2.40
2-Hydroxy-3-methylvaleric acid	101	1.93	97.3	4.07	99.4	3.45	96.0	5.59	103	1.87	101	3.36
2-Hydroxybutyric acid	99.1	1.98	96.9	2.34	97.4	2.74	94.8	6.02	103	1.24	101	4.75
2-Hydroxyglutaric acid	102	0.198	101	2.10	97.6	4.18	92.6	5.43	102	0.677	105	2.34
2-Hydroxyisobutyric acid	108	1.72	102	5.79	98.0	3.91	94.3	5.02	103	0.333	102	1.07
2-Hydroxyisovaleric acid	101	1.20	100	2.39	96.3	1.68	94.7	3.11	104	1.16	105	3.66
2-Hydroxyphenylacetic acid	112	1.12	114	8.59	94.4	5.87	102	7.37	95.5	4.59	100	8.02
2-Oxoadipic acid	94.8	2.24	96.9	7.99	86.8	6.93	93.2	9.93	105	10.3	113	18.1
2-Oxoisocaproic acid	99.7	0.300	100	4.27	93.8	2.95	96.6	3.54	95.5	3.95	103	6.95
3-(3-Hydroxyphenyl)-3-hydroxypropanoic acid	104	3.62	96.7	7.20	96.0	4.51	91.7	4.65	101	0.198	101	3.24
3,4-Dihydroxybutyric acid	104	1.10	105	5.29	108	2.76	105	4.97	105	1.31	107	5.62
3-Aminoisobutyric acid	88.4	2.44	90.6	7.26	96.7	5.89	91.3	9.48	93.6	4.22	97.7	5.44
3-Carboxy-4-methyl-5-propyl-2-furanpropionic acid	98.9	2.44	101	4.05	92.8	1.82	97.6	4.92	93.8	5.46	102	7.88
3-Deoxyglucosone	90.7	4.03	94.5	4.84	90.6	1.17	93.4	3.00	94.1	4.40	98.4	4.50
3-Hydroxybutyric acid	99.6	1.70	97.9	2.21	97.7	2.64	95.0	2.92	103	0.885	102	1.69
3-Hydroxyisobutyric acid	100	2.18	97.4	2.98	98.9	2.97	96.0	3.03	102	0.371	102	1.48
3-Hydroxyisovaleric acid	103	2.62	98.8	4.59	100	2.90	95.4	5.28	103	0.105	103	3.84

3-Hydroxyphenylacetic acid	100	2.47	97.8	6.34	93.4	0.619	93.9	3.16	106	1.57	104	2.77
3-Indoleacetic acid	98.8	1.56	97.3	5.70	97.6	1.59	94.2	3.64	99.0	0.280	99.3	6.87
3-Methyladipic acid	95.9	0.204	95.7	2.29	93.3	1.24	92.1	2.24	101	0.570	101	2.47
4-Ethylphenyl sulfate	102	1.11	101	4.94	95.6	0.564	95.0	5.74	101	1.42	99.3	3.67
4-Hydroxybenzoic acid	100	0.263	100	4.33	93.6	1.73	95.3	8.22	97.9	2.19	95.8	9.17
4-Hydroxyhippuric acid	97.7	1.26	96.4	5.95	99.2	0.279	99.5	3.80	103	0.477	103	2.42
4-Hydroxyphenylacetic acid	95.2	2.93	98.1	3.75	92.6	1.75	91.0	4.36	103	2.47	99.3	4.18
4-Hydroxyphenylpyruvic acid	97.5	0.358	97.2	2.88	92.4	0.505	92.8	4.52	91.6	5.43	99.9	8.27
5-Aminolevulinic Acid	103	0.588	102	5.83	92.7	3.99	96.6	9.08	94.4	1.49	95.9	6.98
5-Hydroxyindoleacetic acid	96.6	0.205	96.4	5.40	101	2.45	98.0	2.90	103	1.32	102	3.85
5-Oxoproline	100	2.21	98.2	3.33	97.2	3.64	93.8	4.81	101	1.18	102	1.19
Acetoacetic acid	90.0	0.938	89.2	3.50	96.9	0.405	97.3	4.12	110	1.88	106	3.54
alpha-Ketoglutaric acid	102	2.90	105	3.99	97.0	3.06	100	3.74	98.8	5.02	104	5.06
alpha-Ketoisovaleric acid	96.9	1.08	97.9	3.09	92.7	0.478	93.1	1.40	98.4	3.92	103	4.20
Argininic acid	97.3	0.630	96.7	5.73	95.7	0.393	95.4	1.61	104	1.74	106	6.90
Benzoic acid	101	0.569	100	5.56	108	1.65	102	5.59	103	5.63	109	6.12
Butyric acid + Isobutyric acid	106	2.16	103	3.16	102	5.39	97.0	6.11	112	2.63	106	6.38
Caffeic acid	103	5.15	97.8	5.72	90.3	1.21	96.3	6.24	105	1.18	104	6.84
Caproic acid	113	4.44	118	7.32	103	2.18	101	4.07	107	1.27	106	1.74
Caprylic acid	98.9	2.02	96.9	4.20	92.8	0.581	93.3	6.53	98.2	1.91	101	2.58
cis-Aconitic acid	93.2	3.29	96.4	6.93	89.6	2.38	91.8	2.90	94.9	2.19	100	5.16
Citric acid	90.9	0.990	90.0	5.32	85.4	2.15	87.3	6.01	90.6	5.66	96.0	5.72
Cyclic AMP	101	1.96	99.2	2.17	95.9	2.49	93.6	6.20	88.9	2.14	95.3	6.72
Dimethylglycine	105	7.41	96.9	8.05	104	2.22	96.6	7.14	104	0.234	102	1.79
Ethylmalonic acid	89.4	0.453	89.0	6.16	83.1	2.73	85.5	4.44	90.1	2.96	96.0	6.12
Fumaric acid	101	1.76	105	3.51	101	1.18	99.7	2.32	108	0.392	107	0.864
Glutaric acid	103	0.274	103	4.37	98.1	1.86	100	3.38	103	1.13	104	2.52
Glyceric acid	92.1	0.565	99.0	7.32	90.5	0.123	93.7	0.885	108	4.05	102	4.98
Guanidinopropionic acid	103	3.90	98.8	4.04	97.0	0.517	97.5	6.70	97.2	1.77	101	3.49
Guanidoacetic acid	93.0	2.25	95.1	2.30	88.0	2.67	92.2	4.55	101	2.51	98.8	6.36
Hippuric acid	102	0.166	102	5.32	93.2	0.609	93.7	3.13	103	1.32	101	3.52

Homovanillic acid	99.7	4.50	105	5.33	91.2	0.904	90.3	9.13	95.9	6.24	102	6.26
Indole-3-carboxylic acid	99.4	2.02	102	3.04	93.1	2.16	95.2	5.61	105	3.52	100	4.98
Indole-3-propionic acid	100	1.20	99.1	3.38	99.1	2.01	97.1	4.36	102	0.447	102	1.01
Indolelactic acid	105	3.16	101	5.89	98.3	3.19	95.2	6.15	106	4.42	100	5.87
Indoxyl glucoside	91.5	2.54	93.9	7.29	86.1	1.31	87.5	1.63	94.5	1.87	92.8	6.96
Indoxyl glucuronide	98.2	1.87	94.2	4.20	96.7	2.72	94.1	4.16	107	1.65	105	2.19
Indoxyl sulfate	103	1.88	101	8.76	101	2.81	98.2	4.46	103	0.766	103	2.91
Isocitric acid	88.4	5.22	87.1	7.34	85.2	2.40	88.5	8.76	95.2	1.67	91.1	6.25
Isovaleric acid	103	1.42	105	2.17	101	1.98	99.0	4.11	102	2.35	99.9	3.16
Kynurenic acid	107	2.62	104	3.09	102	1.90	97.8	3.92	103	3.86	107	5.51
Lactic acid	104	3.23	100	4.78	98.9	3.87	95.2	4.11	106	1.68	103	2.18
Maleic acid	101	1.76	96.0	5.01	96.9	0.746	96.9	1.37	99.9	4.53	105	4.55
Malic acid	99.9	1.48	101	1.57	99.3	1.84	97.5	6.68	101	0.0813	101	1.73
Malonic acid	87.7	1.99	89.5	2.60	91.1	1.92	89.4	5.06	96.4	0.896	95.5	2.25
Methylmalonic acid	85.4	2.31	90.3	5.44	90.2	1.66	88.7	8.22	89.7	2.91	97.5	8.00
N1-Acetyl-Lysine	97.2	2.25	95.1	5.46	105	5.42	99.3	12.8	120	4.96	112	7.47
N6-Acetyl-Lysine	94.1	2.64	98.3	4.24	95.6	1.45	97.1	6.93	103	2.90	107	3.11
N-Acetyl-Alanine	96.3	0.916	97.2	2.75	94.6	0.651	94.0	2.94	102	0.276	102	3.93
N-Acetyl-Arginine	105	5.04	97.0	8.17	109	2.61	98.0	11.3	102	2.35	99.8	2.77
N-Acetyl-Asparagine	101	3.29	97.9	7.60	100	4.30	96.0	6.62	100	1.42	98.6	2.30
N-Acetyl-Aspartic acid	94.5	2.30	96.7	5.85	97.6	0.312	98.0	3.42	97.7	2.76	103	5.07
N-Acetyl-Glutamic acid	107	2.72	104	4.91	97.1	0.910	96.2	1.58	100	2.30	103	5.48
N-Acetyl-Glutamine	102	2.17	100	6.67	96.8	0.983	95.9	5.34	90.6	1.62	94.7	4.30
N-Acetyl-Glycine	107	4.06	102	4.91	96.7	2.01	94.8	2.50	102	0.164	102	2.30
N-Acetyl-Histidine	102	0.793	101	8.50	99.7	3.48	96.4	6.40	107	2.97	104	3.46
N-Acetyl-Isoleucine	97.0	0.623	96.9	3.89	97.1	0.450	97.5	2.10	106	2.56	109	7.35
N-Acetyl-Leucine	98.3	4.65	93.9	4.84	98.1	2.81	95.4	8.43	105	0.568	105	2.18
N-Acetyl-Methionine	105	2.70	102	5.78	94.6	0.706	95.2	6.98	99.4	0.778	100	3.28
N-Acetyl-Proline	90.4	1.06	91.4	3.10	91.7	1.28	90.6	4.79	97.9	1.64	99.5	1.88
N-Acetyl-Serine	105	0.659	105	6.78	98.4	1.49	97.0	5.15	103	0.783	102	3.81
N-Acetyl-Tryptophan	103	1.27	105	5.18	98.6	0.501	99.1	1.78	106	4.13	102	5.24

N-Acetyl-Tyrosine	96.2	1.35	94.9	4.84	93.1	1.16	92.0	8.42	97.9	0.907	97.0	4.38
N-Acetyl-Valine	102	1.31	103	5.12	103	0.844	97.7	5.33	106	0.561	102	3.59
N-Methyl-Aspartic acid	97.6	2.35	93.9	3.96	93.1	0.374	88.2	5.53	103	0.848	102	4.17
Orotic acid	105	4.77	100	5.08	99.5	1.44	97.2	2.31	106	0.915	104	1.83
p-Cresol sulfate	101	2.05	101	2.05	88.4	3.88	88.4	3.88	81.9	2.00	89.9	2.00
Phenylacetic acid	102	0.164	102	1.98	97.4	1.91	95.5	4.82	106	1.75	103	2.23
Phenylacetylglutamine	97.7	1.76	96.0	2.81	94.4	1.15	93.3	3.78	101	1.98	98.6	3.90
Picolinic acid	96.0	0.532	95.5	7.95	95.5	0.788	95.6	4.56	101	3.15	105	3.80
Pipecolic acid	97.4	2.39	95.1	5.85	99.6	1.98	97.7	3.54	103	1.19	98.4	4.36
Propionic acid	106	4.24	99.3	4.34	107	2.18	105	6.17	107	0.142	107	2.10
Pyruvic acid	96.4	2.23	94.3	3.05	93.3	3.02	90.5	3.55	96.2	2.87	101	4.75
Quinaldic acid	101	5.19	95.9	5.99	97.2	5.32	92.3	5.47	105	0.875	104	1.65
Quinoline-4-carboxylic acid	99.3	0.147	99.5	2.32	95.8	0.936	95.7	1.82	102	1.66	104	2.32
Quinolinic acid	96.8	0.509	96.3	4.36	95.6	1.74	93.9	6.47	99.2	0.640	99.8	3.35
Salicylic acid	91.9	1.06	94.1	2.34	94.0	1.71	92.4	8.47	101	0.317	101	3.84
Shikimic acid	100	5.11	94.7	6.12	93.9	2.95	96.8	7.08	97.2	4.74	102	6.02
Succinic acid	99.0	1.13	102	2.74	97.8	0.322	97.5	4.77	104	1.43	106	1.98
Tartaric acid	101	10.3	91.0	10.8	94.8	7.38	88.3	10.1	101	1.55	103	1.92
Threonic acid	104	5.76	98.7	6.98	104	3.01	95.0	9.22	103	1.32	101	4.34
Tiglylglycine	103	2.61	100	4.66	97.7	0.769	98.5	1.81	102	1.80	105	2.70
Uric acid	101	2.19	97.8	3.73	97.0	2.33	94.8	3.36	94.6	1.96	99.7	5.13
Valeric acid	99.2	1.64	97.6	7.33	95.7	4.57	91.5	7.07	104	5.22	98.6	9.89
Xanthine	102	1.33	97.8	4.16	95.8	2.65	93.4	4.62	93.1	2.26	98.6	5.53
Xanthosine	102	3.22	97.0	4.83	93.3	0.812	94.1	2.96	93.2	2.72	99.1	6.04

Supplementary Table S5. Recovery Performance in Spiked Pooled Human Serum

Analyte	Recovery (%)		
	Low	Mid	High
1,3-Diaminopropane	114	111	114
1-Methylnicotinamide	96.1	97.3	99.1
3-Methoxytyramine	103	101	101
3-Nitrotyrosine	115	114	118
5-Methoxytryptamine	110	104	102
5-Methyluridine	102	107	108
7-Methylguanine	118	104	99.9
Adenine	113	114	115
Adenosine	109	109	110
Agmatine	93.1	88.2	83.6
Alanine	108	108	104
Allantoin	80.3	84.9	88.0
alpha-Aminoadipic acid	94.1	96.6	90.5
alpha-Aminobutyric acid	91.2	89.8	91.3
Arginine	88.5	88.5	94.8
Asparagine	109	108	112
Aspartic acid	99.7	112	107
Asymmetric dimethylarginine	111	106	106
beta-Alanine	108	109	103
Betaine	105	109	107
Cadaverine	94.4	98.8	98.5
Carnosine	93.6	88.4	92.0
Choline	106	99.4	98.3
cis-4-Hydroxyproline	82.2	82.9	81.7
Citrulline	101	97.1	99.7

Creatine	114	111	114
Creatinine	109	110	112
Cystathionine	104	108	116
Cytidine	89.3	101	103
Cytosine	88.1	87.5	88.5
Deoxyadenosine	106	111	114
Deoxycytidine	105	114	116
Deoxyguanosine	89.5	88.9	88.1
Deoxyinosine	115	114	119
Deoxyuridine	116	120	119
Dimethylamine	117	119	107
DOPA	105	105	106
Dopamine	117	117	113
Epinephrine	83.6	86.4	83.4
Ethanolamine	93.0	90.2	88.0
gamma-Aminobutyric acid	115	114	99.3
Glutamic acid	114	112	114
Glutamine	113	112	118
Glycine	99.0	100	103
Guanine	104.0	99.5	98.7
Guanosine	82.2	83.7	81.4
Histamine	101	95.2	87.5
Histidine	119	114	114
Homoarginine	92.3	90.1	88.1
Homocitrulline	104	102	110
Hypoxanthine	95.4	94.5	91.1
Indole	80.9	81.9	89.0
Indole-3-acetamide	94.5	94.1	91.7
Inosine	92.9	97.4	99.0
Isoleucine	99.1	101	102
Kynurenine	114	114	114

Leucine	112	102	107
Lysine	110	106	101
Methionine	114	116	117
Methionine sulfoxide	108	116	117
Methylamine	97.2	91.1	98.2
Methylhistidine	116	114	119
N1,N12-Diacetylspermine	112	107	104
N1-Acetylspermidine	104	99.8	102
N2-Acetyl-Ornithine	89.9	90.6	91.1
N-Acetylputrescine	110	109	98.7
Nicotinamide ribotide	102	104	98.4
Norepinephrine	106	110	108
Nudifloramide	119	114	115
Ornithine	104	104	101
Phenylalanine	99.0	100	98.6
Phenylethylamine	106	99.9	103.0
Proline	105	106	112
Putrescine	107	113	112
Sarcosine	90.3	89.8	87.7
Serine	114	108	107
Serotonin	102	103	104
Spermidine	95.3	98.5	109
Spermine	107	105	111
Taurine	104	103	101
Threonine	94.0	98.5	103
Thymidine	102	113	104
Thymine	102	109	105
Total dimethylarginine	107	89.9	102
trans-4-Hydroxyproline	92.7	93.5	95.5
Trimethylamine	109.0	93.9	92.6
Trimethylamine N-Oxide	89.3	90.1	89.5

Tryptamine	101	106	106
Tryptophan	106	105	102
Tyramine	95.7	93.6	89.4
Tyrosine	105	102	103
Uracil	91.3	90.2	86.4
Urea	101	101	101
Uridine	108	113	112
Valine	118	107	107
2,5-Furandicarboxylic acid	88.2	91.5	92.5
2-Hydroxy-2-methylbutyric acid	81.9	85.4	82.7
2-Hydroxy-3-methylvaleric acid	94.4	91.6	87.4
2-Hydroxybutyric acid	89.6	93.1	89.1
2-Hydroxyglutaric acid	90.8	89.2	86.4
2-Hydroxyisobutyric acid	89.5	94.3	90.3
2-Hydroxyisovaleric acid	86.0	97.9	89.6
2-Hydroxyphenylacetic acid	91.2	93.6	94.7
2-Oxoadipic acid	94.2	102	91.8
2-Oxoisocaproic acid	92.6	98.0	103
3-(3-Hydroxyphenyl)-3-hydroxypropanoic acid	88.4	90.3	86.7
3,4-Dihydroxybutyric acid	90.4	85.1	84.0
3-Aminoisobutyric acid	80.3	90.1	81.6
3-Carboxy-4-methyl-5-propyl-2-furanpropionic acid	100	101	104
3-Deoxyglucosone	97.4	102	109
3-Hydroxybutyric acid	89.0	93.8	89.2
3-Hydroxyisobutyric acid	93.2	94.6	90.3
3-Hydroxyisovaleric acid	91.6	91.5	88.9
3-Hydroxyphenylacetic acid	89.2	91.7	88.9
3-Indoleacetic acid	90.1	94.7	86.9
3-Methyladipic acid	95.6	90.4	90.0
4-Ethylphenyl sulfate	90.2	90.4	85.8

4-Hydroxybenzoic acid	90.1	87.0	84.5
4-Hydroxyhippuric acid	88.9	92.1	87.9
4-Hydroxyphenylacetic acid	90.9	95.6	91.7
4-Hydroxyphenylpyruvic acid	85.0	88.1	92.8
5-Aminolevulinic Acid	105	106	93.4
5-Hydroxyindoleacetic acid	86.5	94.7	85.7
5-Oxoproline	91.8	95.0	90.5
Acetoacetic acid	94.5	103	101
alpha-Ketoglutaric acid	83.3	87.3	88.0
alpha-Ketoisovaleric acid	102	111	117
Argininic acid	90.0	90.4	85.4
Benzoic acid	96.6	100	95.9
Butyric acid + Isobutyric acid	100	97.6	93.9
Caffeic acid	84.1	85.5	87.6
Caproic acid	118	110	101
Caprylic acid	99.7	104	101
cis-Aconitic acid	94.3	96.4	102
Citric acid	92.2	93.4	91.6
Cyclic AMP	90.5	84.9	82.7
Dimethylglycine	90.9	94.3	89.8
Ethylmalonic acid	87.4	87.1	84.9
Fumaric acid	92.1	99.1	95.1
Glutaric acid	98.3	101	96.0
Glyceric acid	90.0	91.8	87.4
Guanidinopropionic acid	82.6	83.3	83.4
Guanidoacetic acid	84.8	87.3	85.6
Hippuric acid	95.3	93.2	90.5
Homovanillic acid	80.4	94.8	97.2
Indole-3-carboxylic acid	105	104	100
Indole-3-propionic acid	95.5	97.2	92.3
Indolelactic acid	96.2	96.4	91.9

Indoxyl glucoside	101	106	94.1
Indoxyl glucuronide	87.6	86.8	85.9
Indoxyl sulfate	94.5	98.4	92.7
Isocitric acid	83.7	80.0	82.9
Isovaleric acid	103	102	96.8
Kynurenic acid	91.8	99.4	92.2
Lactic acid	92.9	95.4	91.9
Maleic acid	110	109	103
Malic acid	84.8	84.2	81.1
Malonic acid	83.4	85.3	81.4
Methylmalonic acid	84.4	84.5	80.3
N1-Acetyl-Lysine	89.0	89.6	84.0
N6-Acetyl-Lysine	99.4	100	97.1
N-Acetyl-Alanine	90.4	90.4	93.2
N-Acetyl-Arginine	89.8	87.6	85.0
N-Acetyl-Asparagine	89.1	88.7	86.3
N-Acetyl-Aspartic acid	91.0	92.2	88.7
N-Acetyl-Glutamic acid	83.0	83.3	81.3
N-Acetyl-Glutamine	95.6	98.0	89.2
N-Acetyl-Glycine	97.2	96.7	94.0
N-Acetyl-Histidine	90.8	93.0	89.0
N-Acetyl-Isoleucine	94.0	95.8	90.6
N-Acetyl-Leucine	93.4	98.2	93.0
N-Acetyl-Methionine	97.9	98.4	94.1
N-Acetyl-Proline	96.5	92.6	98.0
N-Acetyl-Serine	97.8	96.7	91.6
N-Acetyl-Tryptophan	95.9	104	94.6
N-Acetyl-Tyrosine	90.8	92.0	91.1
N-Acetyl-Valine	99.7	96.5	99.2
N-Methyl-Aspartic acid	95.4	92.4	93.3
Orotic acid	93.5	94.3	93.7

p-Cresol sulfate	81.3	80.7	82.7
Phenylacetic acid	99.0	97.2	91.4
Phenylacetylglutamine	93.1	93.8	89.8
Picolinic acid	91.1	91.8	87.2
Pipecolic acid	105	109	95.2
Propionic acid	103	105	105
Pyruvic acid	93.9	98.6	95.9
Quinaldic acid	90.4	89.7	87.8
Quinoline-4-carboxylic acid	95.0	94.9	96.6
Quinolinic acid	83.8	84.9	82.5
Salicylic acid	92.7	94.9	93.8
Shikimic acid	91.9	92.5	87.2
Succinic acid	93.4	96.3	90.9
Tartaric acid	99.5	100	96.4
Threonic acid	93.0	91.2	86.0
Tiglylglycine	93.7	95.8	91.1
Uric acid	95.0	89.7	89.0
Valeric acid	91.2	93.6	92.2
Xanthine	92.0	89.3	87.6
Xanthosine	92.0	87.7	87.8

Supplementary Table S6. Validation Performance of Analytes Analyzed *via* DFI-MS/MS

Analyte	Full Name	LOD (μM)	Accuracy (%)			CV (%)			Recovery (%)		
			Low	Mid	High	Low	Mid	High	Low	Mid	High
C0	Carnitine	0.222	101	104	90.6	13.0	7.82	1.25	97.3	109	106
C10	Decanoylcarnitine	0.0585	112	104	101	4.37	3.42	3.81	95.2	91.3	99.9
C10:1	Decenoylcarnitine	0.118	/	/	/	/	/	/			
C10:2	Decadienylcarnitine	0.0490	/	/	/	/	/	/			
C12	Dodecanoylcarnitine	0.0443	107	110	105	2.89	2.69	2.20	106	102	105
C12:1	Dodecenoylcarnitine	0.0786	/	/	/	/	/	/			
C12DC	Dodecanedioylcarnitine	0.0148	/	/	/	/	/	/			
C14	Tetradecanoylcarnitine	0.0320	107	111	107	3.61	3.07	2.73	89.4	93.2	92.3
C14:1	Tetradecenoylcarnitine	0.0716	/	/	/	/	/	/			
C14:1OH	Hydroxytetradecenoylcarnitine	0.0560	/	/	/	/	/	/			
C14:2	Tetradecadienylcarnitine	0.0519	/	/	/	/	/	/			
C14:2OH	Hydroxytetradecadienylcarnitine	0.0542	/	/	/	/	/	/			
C16	Hexadecanoylcarnitine	0.0265	106	104	108	0.963	2.69	0.899	101	92.3	93.3
C16:1	Hexadecenoylcarnitine	0.0201	/	/	/	/	/	/			
C16:1OH	Hydroxyhexadecenoylcarnitine	0.0491	/	/	/	/	/	/			
C16:2	Hexadecadienylcarnitine	0.0463	/	/	/	/	/	/			
C16:2OH	Hydroxyhexadecadienylcarnitine	0.0368	/	/	/	/	/	/			
C16OH	Hydroxyhexadecanoylcarnitine	0.0426	/	/	/	/	/	/			
C18	Octadecanoylcarnitine	0.0237	97.8	96.4	100	2.05	2.04	1.48	90.3	91.2	92.5
C18:1	Octadecenoylcarnitine	0.0411	/	/	/	/	/	/			
C18:1OH	Hydroxyoctadecenoylcarnitine	0.0568	/	/	/	/	/	/			
C18:2	Octadecadienylcarnitine	0.0581	/	/	/	/	/	/			
C2	Acetylcarnitine	0.135	82.9	99.1	102	8.98	5.24	3.79	89.0	89.2	88.1
C3	Propionylcarnitine	0.0824	96.0	100	96.8	3.88	2.03	6.09	98.2	96.1	97.2
C3:1	Propenoylcarnitine	0.0256	/	/	/	/	/	/			

C3OH	Hydroxypropionylcarnitine	0.0337	/	/	/	/	/	/			
C4	Butyrylcarnitine	0.0486	100	98.3	101	1.34	2.04	1.01	91.3	98.3	94.5
C4:1	Butenylcarnitine	0.0240	/	/	/	/	/	/			
C4OH	Hydroxybutyrylcarnitine	0.0237	/	/	/	/	/	/			
C5	Valerylcarnitine	0.0256	/	/	/	/	/	/			
C5:1	Tiglylcarnitine	0.0305	/	/	/	/	/	/			
C5:1DC	Glutaconylcarnitin	0.0250	/	/	/	/	/	/			
C5DC	Glutarylcarnitine	0.0191	/	/	/	/	/	/			
C5MDC	Methylglutarylcarnitine	0.0218	/	/	/	/	/	/			
C5OH	Hydroxyvalerylcarnitine	0.0341	/	/	/	/	/	/			
C6	Hexanoylcarnitine	0.0929	108	104	101	2.00	3.76	3.72	105	99.2	102
C6:1	Hexenoylcarnitine	0.0247	/	/	/	/	/	/			
C7DC	Pimelylcarnitine	0.0200	/	/	/	/	/	/			
C8	Octanoylcarnitine	0.0316	101	95.5	93.7	3.32	4.61	4.03	97.3	99.2	93.5
C9	Nonaylcarnitine	0.0110	/	/	/	/	/	/			
CE(14:0)	Cholesterol ester(14:0)	0.235	/	/	/	/	/	/			
CE(14:1)	Cholesterol ester(14:1)	0.242	/	/	/	/	/	/			
CE(15:0)	Cholesterol ester(15:0)	0.180	/	/	/	/	/	/			
CE(15:1)	Cholesterol ester(15:1)	0.0759	/	/	/	/	/	/			
CE(16:0)	Cholesterol ester(16:0)	0.435	/	/	/	/	/	/			
CE(16:1)	Cholesterol ester(16:1)	0.596	/	/	/	/	/	/			
CE(17:0)	Cholesterol ester(17:0)	0.126	98.9	97.0	98.3	6.91	9.79	6.76	110	108	94.5
CE(17:1)	Cholesterol ester(17:1)	0.256	/	/	/	/	/	/			
CE(18:0)	Cholesterol ester(18:0)	0.232	/	/	/	/	/	/			
CE(18:1)	Cholesterol ester(18:1)	0.368	/	/	/	/	/	/			
CE(18:2)	Cholesterol ester(18:2)	0.510	/	/	/	/	/	/			
CE(18:3)	Cholesterol ester(18:3)	0.218	/	/	/	/	/	/			
CE(20:0)	Cholesterol ester(20:0)	0.258	/	/	/	/	/	/			
CE(20:1)	Cholesterol ester(20:1)	0.143	/	/	/	/	/	/			
CE(20:3)	Cholesterol ester(20:3)	0.111	/	/	/	/	/	/			
CE(20:4)	Cholesterol ester(20:4)	0.111	/	/	/	/	/	/			

CE(20:5)	Cholesterol ester(20:5)	0.513	/	/	/	/	/	/			
CE(22:0)	Cholesterol ester(22:0)	0.229	/	/	/	/	/	/			
CE(22:1)	Cholesterol ester(22:1)	0.137	/	/	/	/	/	/			
CE(22:2)	Cholesterol ester(22:2)	0.0950	/	/	/	/	/	/			
CE(22:5)	Cholesterol ester(22:5)	0.114	/	/	/	/	/	/			
CE(22:6)	Cholesterol ester(22:6)	0.0640	/	/	/	/	/	/			
Cer(d16:1/18:0)	Ceramide(d16:1/18:0)	0.0545	/	/	/	/	/	/			
Cer(d16:1/20:0)	Ceramide(d16:1/20:0)	0.0245	/	/	/	/	/	/			
Cer(d16:1/22:0)	Ceramide(d16:1/22:0)	0.0125	/	/	/	/	/	/			
Cer(d16:1/23:0)	Ceramide(d16:1/23:0)	0.0200	/	/	/	/	/	/			
Cer(d16:1/24:0)	Ceramide(d16:1/24:0)	0.0185	/	/	/	/	/	/			
Cer(d18:0/18:0(OH))	Ceramide(d18:0/18:0(OH))	0.0399	/	/	/	/	/	/			
Cer(d18:0/18:0)	Ceramide(d18:0/18:0)	0.0420	/	/	/	/	/	/			
Cer(d18:0/20:0)	Ceramide(d18:0/20:0)	0.0229	/	/	/	/	/	/			
Cer(d18:0/22:0)	Ceramide(d18:0/22:0)	0.0108	/	/	/	/	/	/			
Cer(d18:0/24:0)	Ceramide(d18:0/24:0)	0.0106	/	/	/	/	/	/			
Cer(d18:0/24:1)	Ceramide(d18:0/24:1)	0.0108	/	/	/	/	/	/			
Cer(d18:0/26:1(OH))	Ceramide(d18:0/26:1(OH))	0.0190	/	/	/	/	/	/			
Cer(d18:0/26:1)	Ceramide(d18:0/26:1)	0.0330	/	/	/	/	/	/			
Cer(d18:1/14:0)	Ceramide(d18:1/14:0)	0.0332	/	/	/	/	/	/			
Cer(d18:1/16:0)	Ceramide(d18:1/16:0)	0.0320	/	/	/	/	/	/			
Cer(d18:1/18:0(OH))	Ceramide(d18:1/18:0(OH))	0.0125	/	/	/	/	/	/			
Cer(d18:1/18:0)	Ceramide(d18:1/18:0)	0.0104	112	114	120	3.23	2.20	4.51	96.3	91.2	97.2
Cer(d18:1/18:1)	Ceramide(d18:1/18:1)	0.0198	/	/	/	/	/	/			
Cer(d18:1/20:0(OH))	Ceramide(d18:1/20:0(OH))	0.0220	/	/	/	/	/	/			
Cer(d18:1/20:0)	Ceramide(d18:1/20:0)	0.0250	/	/	/	/	/	/			
Cer(d18:1/22:0)	Ceramide(d18:1/22:0)	0.0197	/	/	/	/	/	/			
Cer(d18:1/23:0)	Ceramide(d18:1/23:0)	0.0262	/	/	/	/	/	/			
Cer(d18:1/24:0)	Ceramide(d18:1/24:0)	0.0151	/	/	/	/	/	/			
Cer(d18:1/24:1)	Ceramide(d18:1/24:1)	0.0329	/	/	/	/	/	/			
Cer(d18:1/25:0)	Ceramide(d18:1/25:0)	0.120	/	/	/	/	/	/			

Cer(d18:1/26:0)	Ceramide(d18:1/26:0)	0.1107	/	/	/	/	/	/			
Cer(d18:1/26:1)	Ceramide(d18:1/26:1)	0.1093	/	/	/	/	/	/			
Cer(d18:2/14:0)	Ceramide(d18:2/14:0)	0.1299	/	/	/	/	/	/			
Cer(d18:2/16:0)	Ceramide(d18:2/16:0)	0.0997	/	/	/	/	/	/			
Cer(d18:2/18:0)	Ceramide(d18:2/18:0)	0.1098	/	/	/	/	/	/			
Cer(d18:2/18:1)	Ceramide(d18:2/18:1)	0.1250	/	/	/	/	/	/			
Cer(d18:2/20:0)	Ceramide(d18:2/20:0)	0.1130	/	/	/	/	/	/			
Cer(d18:2/22:0)	Ceramide(d18:2/22:0)	0.0101	/	/	/	/	/	/			
Cer(d18:2/23:0)	Ceramide(d18:2/23:0)	0.0211	/	/	/	/	/	/			
Cer(d18:2/24:0)	Ceramide(d18:2/24:0)	0.0104	/	/	/	/	/	/			
Cer(d18:2/24:1)	Ceramide(d18:2/24:1)	0.0140	/	/	/	/	/	/			
DG(14:0/14:0)	Diacylglycerol(14:0/14:0)	0.0988	/	/	/	/	/	/			
DG(14:0/18:1)	Diacylglycerol(14:0/18:1)	0.0884	/	/	/	/	/	/			
DG(14:0/18:2)	Diacylglycerol(14:0/18:2)	0.0660	/	/	/	/	/	/			
DG(14:0/20:0)	Diacylglycerol(14:0/20:0)	0.128	/	/	/	/	/	/			
DG(14:1/18:1)	Diacylglycerol(14:1/18:1)	0.125	/	/	/	/	/	/			
DG(14:1/20:2)	Diacylglycerol(14:1/20:2)	0.120	/	/	/	/	/	/			
DG(16:0/16:0)	Diacylglycerol(16:0/16:0)	0.180	/	/	/	/	/	/			
DG(16:0/16:1)	Diacylglycerol(16:0/16:1)	0.0271	/	/	/	/	/	/			
DG(16:0/18:1)	Diacylglycerol(16:0/18:1)	0.0270	/	/	/	/	/	/			
DG(16:0/18:2)	Diacylglycerol(16:0/18:2)	0.0511	/	/	/	/	/	/			
DG(16:0/20:0)	Diacylglycerol(16:0/20:0)	0.0338	/	/	/	/	/	/			
DG(16:0/20:3)	Diacylglycerol(16:0/20:3)	0.0470	/	/	/	/	/	/			
DG(16:0/20:4)	Diacylglycerol(16:0/20:4)	0.0600	/	/	/	/	/	/			
DG(16:1/18:0)	Diacylglycerol(16:1/18:0)	0.236	/	/	/	/	/	/			
DG(16:1/18:1)	Diacylglycerol(16:1/18:1)	0.158	/	/	/	/	/	/			
DG(16:1/18:2)	Diacylglycerol(16:1/18:2)	0.0965	/	/	/	/	/	/			
DG(16:1/20:0)	Diacylglycerol(16:1/20:0)	0.0748	/	/	/	/	/	/			
DG(17:0/17:1)	Diacylglycerol(17:0/17:1)	0.0150	/	/	/	/	/	/			
DG(17:0/18:1)	Diacylglycerol(17:0/18:1)	0.0109	/	/	/	/	/	/			
DG(18:0/20:0)	Diacylglycerol(18:0/20:0)	0.172	/	/	/	/	/	/			

DG(18:0/20:4)	Diacylglycerol(18:0/20:4)	0.123	/	/	/	/	/	/			
DG(18:1/18:1)	Diacylglycerol(18:1/18:1)	0.0356	111	111	112	4.99	4.47	5.63	88.2	89.1	87.3
DG(18:1/18:2)	Diacylglycerol(18:1/18:2)	0.0161	/	/	/	/	/	/			
DG(18:1/18:3)	Diacylglycerol(18:1/18:3)	0.0212	/	/	/	/	/	/			
DG(18:1/18:4)	Diacylglycerol(18:1/18:4)	0.0324	/	/	/	/	/	/			
DG(18:1/20:0)	Diacylglycerol(18:1/20:0)	0.0325	/	/	/	/	/	/			
DG(18:1/20:1)	Diacylglycerol(18:1/20:1)	0.0435	/	/	/	/	/	/			
DG(18:1/20:2)	Diacylglycerol(18:1/20:2)	0.0330	/	/	/	/	/	/			
DG(18:1/20:3)	Diacylglycerol(18:1/20:3)	0.0123	/	/	/	/	/	/			
DG(18:1/20:4)	Diacylglycerol(18:1/20:4)	0.0109	/	/	/	/	/	/			
DG(18:1/22:5)	Diacylglycerol(18:1/22:5)	0.0326	/	/	/	/	/	/			
DG(18:1/22:6)	Diacylglycerol(18:1/22:6)	0.0674	/	/	/	/	/	/			
DG(18:2/18:2)	Diacylglycerol(18:2/18:2)	0.0351	/	/	/	/	/	/			
DG(18:2/18:3)	Diacylglycerol(18:2/18:3)	0.0435	/	/	/	/	/	/			
DG(18:2/18:4)	Diacylglycerol(18:2/18:4)	0.0231	/	/	/	/	/	/			
DG(18:2/20:0)	Diacylglycerol(18:2/20:0)	0.0511	/	/	/	/	/	/			
DG(18:2/20:4)	Diacylglycerol(18:2/20:4)	0.0433	/	/	/	/	/	/			
DG(18:3/18:3)	Diacylglycerol(18:3/18:3)	0.0691	/	/	/	/	/	/			
DG(18:3/20:2)	Diacylglycerol(18:3/20:2)	0.0330	/	/	/	/	/	/			
DG(21:0/22:6)	Diacylglycerol(21:0/22:6)	0.0549	/	/	/	/	/	/			
DG(22:1/22:2)	Diacylglycerol(22:1/22:2)	0.0733	/	/	/	/	/	/			
DG-O(14:0/18:2)	Diacylglycerol-O(14:0/18:2)	0.0567	/	/	/	/	/	/			
DG-O(16:0/18:1)	Diacylglycerol-O(16:0/18:1)	0.0608	/	/	/	/	/	/			
DG-O(16:0/20:4)	Diacylglycerol-O(16:0/20:4)	0.0454	/	/	/	/	/	/			
LacCer(d18:1/14:0)	LacCer(d18:1/14:0)	0.0107	/	/	/	/	/	/			
LacCer(d18:1/16:0)	LacCer(d18:1/16:0)	0.0143	/	/	/	/	/	/			
LacCer(d18:1/18:0)	LacCer(d18:1/18:0)	0.0104	105	106	110	4.14	3.60	1.61	109	116	102
LacCer(d18:1/20:0)	LacCer(d18:1/20:0)	0.0146	/	/	/	/	/	/			
LacCer(d18:1/22:0)	LacCer(d18:1/22:0)	0.0119	/	/	/	/	/	/			
LacCer(d18:1/24:0)	LacCer(d18:1/24:0)	0.0758	/	/	/	/	/	/			
LacCer(d18:1/24:1)	LacCer(d18:1/24:1)	0.0124	/	/	/	/	/	/			

LacCer(d18:1/26:0)	LacCer(d18:1/26:0)	0.0437	/	/	/	/	/	/			
LacCer(d18:1/26:1)	LacCer(d18:1/26:1)	0.0340	/	/	/	/	/	/			
Trihexosylceramide(d18:1/16:0)	Trihexosylceramide(d18:1/16:0)	0.0154	/	/	/	/	/	/			
Trihexosylceramide(d18:1/18:0)	Trihexosylceramide(d18:1/18:0)	0.0136	/	/	/	/	/	/			
Trihexosylceramide(d18:1/20:0)	Trihexosylceramide(d18:1/20:0)	0.0125	/	/	/	/	/	/			
Trihexosylceramide(d18:1/24:1)	Trihexosylceramide(d18:1/24:1)	0.0171	/	/	/	/	/	/			
Trihexosylceramide(d18:1/26:1)	Trihexosylceramide(d18:1/26:1)	0.0235	/	/	/	/	/	/			
Trihexosylceramide(d18:1/22:0)	Trihexosylceramide(d18:1/22:0)	0.0131	/	/	/	/	/	/			
GlcCer(d16:1/22:0)	GlcCer(d16:1/22:0)	0.0106	/	/	/	/	/	/			
GlcCer(d16:1/24:0)	GlcCer(d16:1/24:0)	0.0330	/	/	/	/	/	/			
GlcCer(d18:1/14:0)	GlcCer(d18:1/14:0)	0.0300	/	/	/	/	/	/			
GlcCer(d18:1/16:0)	GlcCer(d18:1/16:0)	0.0279	/	/	/	/	/	/			
GlcCer(d18:1/18:0)	GlcCer(d18:1/18:0)	0.0180	107	108	113	2.37	2.33	2.64	97.4	92.4	106
GlcCer(d18:1/18:1)	GlcCer(d18:1/18:1)	0.0193	/	/	/	/	/	/			
GlcCer(d18:1/20:0)	GlcCer(d18:1/20:0)	0.0109	/	/	/	/	/	/			
GlcCer(d18:1/22:0)	GlcCer(d18:1/22:0)	0.0191	/	/	/	/	/	/			
GlcCer(d18:1/23:0)	GlcCer(d18:1/23:0)	0.0108	/	/	/	/	/	/			
GlcCer(d18:1/24:0)	GlcCer(d18:1/24:0)	0.0327	/	/	/	/	/	/			
GlcCer(d18:1/24:1)	GlcCer(d18:1/24:1)	0.104	/	/	/	/	/	/			
GlcCer(d18:1/26:0)	GlcCer(d18:1/26:0)	0.0371	/	/	/	/	/	/			
GlcCer(d18:1/26:1)	GlcCer(d18:1/26:1)	0.0105	/	/	/	/	/	/			
GlcCer(d18:2/16:0)	GlcCer(d18:2/16:0)	0.0103	/	/	/	/	/	/			
GlcCer(d18:2/18:0)	GlcCer(d18:2/18:0)	0.0124	/	/	/	/	/	/			
GlcCer(d18:2/20:0)	GlcCer(d18:2/20:0)	0.0111	/	/	/	/	/	/			
GlcCer(d18:2/22:0)	GlcCer(d18:2/22:0)	0.0109	/	/	/	/	/	/			
GlcCer(d18:2/23:0)	GlcCer(d18:2/23:0)	0.0107	/	/	/	/	/	/			
GlcCer(d18:2/24:0)	GlcCer(d18:2/24:0)	0.0199	/	/	/	/	/	/			
Hexose	Hexose	22.5	99.1	100	101	1.88	0.961	3.92	103	97.2	105
LysoPC a C14:0	LysoPhosphatidylcholine acyl C14:0	0.130	/	/	/	/	/	/			
LysoPC a C16:0	LysoPhosphatidylcholine acyl C16:0	0.108	/	/	/	/	/	/			
LysoPC a C16:1	LysoPhosphatidylcholine acyl C16:1	0.0747	/	/	/	/	/	/			

LysoPC a C17:0	LysoPhosphatidylcholine acyl C17:0	0.0164	/	/	/	/	/	/			
LysoPC a C18:0	LysoPhosphatidylcholine acyl C18:0	0.0532	99.3	93.3	93.3	3.34	2.55	2.99	92.5	98.3	91.2
LysoPC a C18:1	LysoPhosphatidylcholine acyl C18:1	0.0518	110	102	100	3.54	3.18	1.47	103	96.3	105
LysoPC a C18:2	LysoPhosphatidylcholine acyl C18:2	0.0399	/	/	/	/	/	/			
LysoPC a C20:3	LysoPhosphatidylcholine acyl C20:3	0.0895	/	/	/	/	/	/			
LysoPC a C20:4	LysoPhosphatidylcholine acyl C20:4	0.0282	/	/	/	/	/	/			
LysoPC a C24:0	LysoPhosphatidylcholine acyl C24:0	0.0374	/	/	/	/	/	/			
LysoPC a C26:0	LysoPhosphatidylcholine acyl C26:0	0.129	/	/	/	/	/	/			
LysoPC a C26:1	LysoPhosphatidylcholine acyl C26:1	0.0327	/	/	/	/	/	/			
LysoPC a C28:0	LysoPhosphatidylcholine acyl C28:0	0.0715	/	/	/	/	/	/			
LysoPC a C28:1	LysoPhosphatidylcholine acyl C28:1	0.0519	/	/	/	/	/	/			
PC aa C24:0	Phosphatidylcholine diacyl C24:0	0.0767	/	/	/	/	/	/			
PC aa C26:0	Phosphatidylcholine diacyl C26:0	0.640	/	/	/	/	/	/			
PC aa C28:1	Phosphatidylcholine diacyl C28:1	0.0278	/	/	/	/	/	/			
PC aa C30:0	Phosphatidylcholine diacyl C30:0	0.108	/	/	/	/	/	/			
PC aa C32:0	Phosphatidylcholine diacyl C32:0	0.0674	/	/	/	/	/	/			
PC aa C32:1	Phosphatidylcholine diacyl C32:1	0.0586	/	/	/	/	/	/			
PC aa C32:2	Phosphatidylcholine diacyl C32:2	0.0742	/	/	/	/	/	/			
PC aa C32:3	Phosphatidylcholine diacyl C32:3	0.0203	/	/	/	/	/	/			
PC aa C34:1	Phosphatidylcholine diacyl C34:1	0.437	/	/	/	/	/	/			
PC aa C34:2	Phosphatidylcholine diacyl C34:2	0.289	/	/	/	/	/	/			
PC aa C34:3	Phosphatidylcholine diacyl C34:3	0.0537	/	/	/	/	/	/			
PC aa C34:4	Phosphatidylcholine diacyl C34:4	0.0420	/	/	/	/	/	/			
PC aa C36:0	Phosphatidylcholine diacyl C36:0	0.159	101	104	105	4.50	1.63	2.34	92.3	107	96.2
PC aa C36:1	Phosphatidylcholine diacyl C36:1	0.123	/	/	/	/	/	/			
PC aa C36:2	Phosphatidylcholine diacyl C36:2	0.121	/	/	/	/	/	/			
PC aa C36:3	Phosphatidylcholine diacyl C36:3	0.128	/	/	/	/	/	/			
PC aa C36:4	Phosphatidylcholine diacyl C36:4	0.146	/	/	/	/	/	/			
PC aa C36:5	Phosphatidylcholine diacyl C36:5	0.0600	/	/	/	/	/	/			
PC aa C36:6	Phosphatidylcholine diacyl C36:6	0.0332	/	/	/	/	/	/			
PC aa C38:0	Phosphatidylcholine diacyl C38:0	0.0369	/	/	/	/	/	/			

PC aa C38:1	Phosphatidylcholine diacyl C38:1	0.0449	/	/	/	/	/	/			
PC aa C38:3	Phosphatidylcholine diacyl C38:3	0.0233	/	/	/	/	/	/			
PC aa C38:4	Phosphatidylcholine diacyl C38:4	0.0700	/	/	/	/	/	/			
PC aa C38:5	Phosphatidylcholine diacyl C38:5	0.0732	/	/	/	/	/	/			
PC aa C38:6	Phosphatidylcholine diacyl C38:6	0.0545	/	/	/	/	/	/			
PC aa C40:1	Phosphatidylcholine diacyl C40:1	0.0194	/	/	/	/	/	/			
PC aa C40:2	Phosphatidylcholine diacyl C40:2	0.0174	/	/	/	/	/	/			
PC aa C40:3	Phosphatidylcholine diacyl C40:3	0.0326	/	/	/	/	/	/			
PC aa C40:4	Phosphatidylcholine diacyl C40:4	0.0205	/	/	/	/	/	/			
PC aa C40:5	Phosphatidylcholine diacyl C40:5	0.0260	/	/	/	/	/	/			
PC aa C40:6	Phosphatidylcholine diacyl C40:6	0.0470	/	/	/	/	/	/			
PC aa C42:0	Phosphatidylcholine diacyl C42:0	0.0337	/	/	/	/	/	/			
PC aa C42:1	Phosphatidylcholine diacyl C42:1	0.0517	/	/	/	/	/	/			
PC aa C42:2	Phosphatidylcholine diacyl C42:2	0.0229	/	/	/	/	/	/			
PC aa C42:4	Phosphatidylcholine diacyl C42:4	0.0186	/	/	/	/	/	/			
PC aa C42:5	Phosphatidylcholine diacyl C42:5	0.0181	/	/	/	/	/	/			
PC aa C42:6	Phosphatidylcholine diacyl C42:6	0.0360	/	/	/	/	/	/			
PC ae C30:0	Phosphatidylcholine acyl-alkyl C30:0	0.0959	/	/	/	/	/	/			
PC ae C30:1	Phosphatidylcholine acyl-alkyl C30:1	0.0364	/	/	/	/	/	/			
PC ae C30:2	Phosphatidylcholine acyl-alkyl C30:2	0.0287	/	/	/	/	/	/			
PC ae C32:1	Phosphatidylcholine acyl-alkyl C32:1	0.0365	/	/	/	/	/	/			
PC ae C32:2	Phosphatidylcholine acyl-alkyl C32:2	0.0387	/	/	/	/	/	/			
PC ae C34:0	Phosphatidylcholine acyl-alkyl C34:0	0.0731	/	/	/	/	/	/			
PC ae C34:1	Phosphatidylcholine acyl-alkyl C34:1	0.0373	/	/	/	/	/	/			
PC ae C34:2	Phosphatidylcholine acyl-alkyl C34:2	0.0334	/	/	/	/	/	/			
PC ae C34:3	Phosphatidylcholine acyl-alkyl C34:3	0.0260	/	/	/	/	/	/			
PC ae C36:0	Phosphatidylcholine acyl-alkyl C36:0	0.0477	/	/	/	/	/	/			
PC ae C36:1	Phosphatidylcholine acyl-alkyl C36:1	0.0468	/	/	/	/	/	/			
PC ae C36:2	Phosphatidylcholine acyl-alkyl C36:2	0.0220	/	/	/	/	/	/			
PC ae C36:3	Phosphatidylcholine acyl-alkyl C36:3	0.0139	/	/	/	/	/	/			
PC ae C36:4	Phosphatidylcholine acyl-alkyl C36:4	0.0256	/	/	/	/	/	/			

PC ae C36:5	Phosphatidylcholine acyl-alkyl C36:5	0.0316	/	/	/	/	/	/			
PC ae C38:0	Phosphatidylcholine acyl-alkyl C38:0	0.0248	/	/	/	/	/	/			
PC ae C38:1	Phosphatidylcholine acyl-alkyl C38:1	0.0361	/	/	/	/	/	/			
PC ae C38:2	Phosphatidylcholine acyl-alkyl C38:2	0.0403	/	/	/	/	/	/			
PC ae C38:3	Phosphatidylcholine acyl-alkyl C38:3	0.0314	/	/	/	/	/	/			
PC ae C38:4	Phosphatidylcholine acyl-alkyl C38:4	0.0333	/	/	/	/	/	/			
PC ae C38:5	Phosphatidylcholine acyl-alkyl C38:5	0.0155	/	/	/	/	/	/			
PC ae C38:6	Phosphatidylcholine acyl-alkyl C38:6	0.0393	/	/	/	/	/	/			
PC ae C40:1	Phosphatidylcholine acyl-alkyl C40:1	0.0173	/	/	/	/	/	/			
PC ae C40:2	Phosphatidylcholine acyl-alkyl C40:2	0.0343	/	/	/	/	/	/			
PC ae C40:3	Phosphatidylcholine acyl-alkyl C40:3	0.0226	/	/	/	/	/	/			
PC ae C40:4	Phosphatidylcholine acyl-alkyl C40:4	0.0266	/	/	/	/	/	/			
PC ae C40:5	Phosphatidylcholine acyl-alkyl C40:5	0.0123	/	/	/	/	/	/			
PC ae C40:6	Phosphatidylcholine acyl-alkyl C40:6	0.0358	/	/	/	/	/	/			
PC ae C42:0	Phosphatidylcholine acyl-alkyl C42:0	0.0398	/	/	/	/	/	/			
PC ae C42:1	Phosphatidylcholine acyl-alkyl C42:1	0.0288	/	/	/	/	/	/			
PC ae C42:2	Phosphatidylcholine acyl-alkyl C42:2	0.0394	/	/	/	/	/	/			
PC ae C42:3	Phosphatidylcholine acyl-alkyl C42:3	0.0436	/	/	/	/	/	/			
PC ae C42:4	Phosphatidylcholine acyl-alkyl C42:4	0.0133	/	/	/	/	/	/			
PC ae C42:5	Phosphatidylcholine acyl-alkyl C42:5	0.0275	/	/	/	/	/	/			
PC ae C44:3	Phosphatidylcholine acyl-alkyl C44:3	0.0374	/	/	/	/	/	/			
PC ae C44:4	Phosphatidylcholine acyl-alkyl C44:4	0.0215	/	/	/	/	/	/			
PC ae C44:5	Phosphatidylcholine acyl-alkyl C44:5	0.0219	/	/	/	/	/	/			
PC ae C44:6	Phosphatidylcholine acyl-alkyl C44:6	0.0414	/	/	/	/	/	/			
SM C16:0	Sphingomyeline C16:0	0.102	/	/	/	/	/	/			
SM C16:1	Sphingomyeline C16:1	0.0275	/	/	/	/	/	/			
SM C18:0	Sphingomyeline C18:0	0.127	105	97.4	101	3.39	5.00	3.08	109	110	94.2
SM C18:1	Sphingomyeline C18:1	0.0330	/	/	/	/	/	/			
SM C20:2	Sphingomyeline C20:2	0.0171	/	/	/	/	/	/			
SM C24:0	Sphingomyeline C24:0	0.0484	/	/	/	/	/	/			
SM C24:1	Sphingomyeline C24:1	0.0248	/	/	/	/	/	/			

SM C26:0	Sphingomyeline C26:0	0.0201	/	/	/	/	/	/			
SM C26:1	Sphingomyeline C26:1	0.0293	/	/	/	/	/	/			
SMOH C14:1	Hydroxysphingomyeline C14:1	0.0190	/	/	/	/	/	/			
SMOH C16:1	Hydroxysphingomyeline C16:1	0.0315	/	/	/	/	/	/			
SMOH C22:1	Hydroxysphingomyeline C22:1	0.0227	/	/	/	/	/	/			
SMOH C22:2	Hydroxysphingomyeline C22:2	0.0208	/	/	/	/	/	/			
SMOH C24:1	Hydroxysphingomyeline C24:1	0.0211	/	/	/	/	/	/			
TG(14:0/32:2)	Triacylglycerol(14:0/32:2)	0.120	/	/	/	/	/	/			
TG(14:0/34:0)	Triacylglycerol(14:0/34:0)	0.0583	/	/	/	/	/	/			
TG(14:0/34:1)	Triacylglycerol(14:0/34:1)	0.115	/	/	/	/	/	/			
TG(14:0/34:2)	Triacylglycerol(14:0/34:2)	0.250	/	/	/	/	/	/			
TG(14:0/34:3)	Triacylglycerol(14:0/34:3)	0.0862	/	/	/	/	/	/			
TG(14:0/35:1)	Triacylglycerol(14:0/35:1)	0.111	/	/	/	/	/	/			
TG(14:0/35:2)	Triacylglycerol(14:0/35:2)	0.140	/	/	/	/	/	/			
TG(14:0/36:1)	Triacylglycerol(14:0/36:1)	0.0269	/	/	/	/	/	/			
TG(14:0/36:2)	Triacylglycerol(14:0/36:2)	0.118	/	/	/	/	/	/			
TG(14:0/36:3)	Triacylglycerol(14:0/36:3)	0.0355	/	/	/	/	/	/			
TG(14:0/36:4)	Triacylglycerol(14:0/36:4)	0.0325	/	/	/	/	/	/			
TG(14:0/38:4)	Triacylglycerol(14:0/38:4)	0.0324	/	/	/	/	/	/			
TG(14:0/38:5)	Triacylglycerol(14:0/38:5)	0.0277	/	/	/	/	/	/			
TG(14:0/39:3)	Triacylglycerol(14:0/39:3)	0.0899	/	/	/	/	/	/			
TG(16:0/28:1)	Triacylglycerol(16:0/28:1)	0.0983	/	/	/	/	/	/			
TG(16:0/28:2)	Triacylglycerol(16:0/28:2)	0.145	/	/	/	/	/	/			
TG(16:0/30:2)	Triacylglycerol(16:0/30:2)	0.0764	/	/	/	/	/	/			
TG(16:0/32:0)	Triacylglycerol(16:0/32:0)	0.160	/	/	/	/	/	/			
TG(16:0/32:1)	Triacylglycerol(16:0/32:1)	0.179	/	/	/	/	/	/			
TG(16:0/32:2)	Triacylglycerol(16:0/32:2)	0.0446	/	/	/	/	/	/			
TG(16:0/32:3)	Triacylglycerol(16:0/32:3)	0.0617	/	/	/	/	/	/			
TG(16:0/33:1)	Triacylglycerol(16:0/33:1)	0.0572	/	/	/	/	/	/			
TG(16:0/33:2)	Triacylglycerol(16:0/33:2)	0.116	/	/	/	/	/	/			
TG(16:0/34:0)	Triacylglycerol(16:0/34:0)	0.0352	/	/	/	/	/	/			

TG(16:0/34:1)	Triacylglycerol(16:0/34:1)	0.0736	/	/	/	/	/	/			
TG(16:0/34:2)	Triacylglycerol(16:0/34:2)	0.0596	/	/	/	/	/	/			
TG(16:0/34:3)	Triacylglycerol(16:0/34:3)	0.0208	/	/	/	/	/	/			
TG(16:0/34:4)	Triacylglycerol(16:0/34:4)	0.0105	/	/	/	/	/	/			
TG(16:0/35:1)	Triacylglycerol(16:0/35:1)	0.0258	/	/	/	/	/	/			
TG(16:0/35:2)	Triacylglycerol(16:0/35:2)	0.0477	/	/	/	/	/	/			
TG(16:0/35:3)	Triacylglycerol(16:0/35:3)	0.0431	/	/	/	/	/	/			
TG(16:0/36:2)	Triacylglycerol(16:0/36:2)	0.0255	/	/	/	/	/	/			
TG(16:0/36:3)	Triacylglycerol(16:0/36:3)	0.125	/	/	/	/	/	/			
TG(16:0/36:4)	Triacylglycerol(16:0/36:4)	0.113	/	/	/	/	/	/			
TG(16:0/36:5)	Triacylglycerol(16:0/36:5)	0.0664	/	/	/	/	/	/			
TG(16:0/36:6)	Triacylglycerol(16:0/36:6)	0.0348	/	/	/	/	/	/			
TG(16:0/37:3)	Triacylglycerol(16:0/37:3)	0.0577	/	/	/	/	/	/			
TG(16:0/38:1)	Triacylglycerol(16:0/38:1)	0.0741	/	/	/	/	/	/			
TG(16:0/38:2)	Triacylglycerol(16:0/38:2)	0.163	/	/	/	/	/	/			
TG(16:0/38:3)	Triacylglycerol(16:0/38:3)	0.0109	/	/	/	/	/	/			
TG(16:0/38:4)	Triacylglycerol(16:0/38:4)	0.0400	/	/	/	/	/	/			
TG(16:0/38:5)	Triacylglycerol(16:0/38:5)	0.0394	/	/	/	/	/	/			
TG(16:0/38:6)	Triacylglycerol(16:0/38:6)	0.0766	/	/	/	/	/	/			
TG(16:0/38:7)	Triacylglycerol(16:0/38:7)	0.0196	/	/	/	/	/	/			
TG(16:0/40:6)	Triacylglycerol(16:0/40:6)	0.0456	/	/	/	/	/	/			
TG(16:0/40:7)	Triacylglycerol(16:0/40:7)	0.0410	/	/	/	/	/	/			
TG(16:0/40:8)	Triacylglycerol(16:0/40:8)	0.0577	/	/	/	/	/	/			
TG(16:1/28:0)	Triacylglycerol(16:1/28:0)	0.0405	/	/	/	/	/	/			
TG(16:1/30:1)	Triacylglycerol(16:1/30:1)	0.0258	/	/	/	/	/	/			
TG(16:1/32:0)	Triacylglycerol(16:1/32:0)	0.100	/	/	/	/	/	/			
TG(16:1/32:1)	Triacylglycerol(16:1/32:1)	0.191	/	/	/	/	/	/			
TG(16:1/32:2)	Triacylglycerol(16:1/32:2)	0.0469	/	/	/	/	/	/			
TG(16:1/33:1)	Triacylglycerol(16:1/33:1)	0.0206	/	/	/	/	/	/			
TG(16:1/34:0)	Triacylglycerol(16:1/34:0)	0.0161	/	/	/	/	/	/			
TG(16:1/34:1)	Triacylglycerol(16:1/34:1)	0.0399	/	/	/	/	/	/			

TG(16:1/34:2)	Triacylglycerol(16:1/34:2)	0.0484	/	/	/	/	/	/			
TG(16:1/34:3)	Triacylglycerol(16:1/34:3)	0.0731	/	/	/	/	/	/			
TG(16:1/36:1)	Triacylglycerol(16:1/36:1)	0.126	/	/	/	/	/	/			
TG(16:1/36:2)	Triacylglycerol(16:1/36:2)	0.154	/	/	/	/	/	/			
TG(16:1/36:3)	Triacylglycerol(16:1/36:3)	0.0867	/	/	/	/	/	/			
TG(16:1/36:4)	Triacylglycerol(16:1/36:4)	0.0178	/	/	/	/	/	/			
TG(16:1/36:5)	Triacylglycerol(16:1/36:5)	0.0560	/	/	/	/	/	/			
TG(16:1/38:3)	Triacylglycerol(16:1/38:3)	0.0316	/	/	/	/	/	/			
TG(16:1/38:4)	Triacylglycerol(16:1/38:4)	0.0634	/	/	/	/	/	/			
TG(16:1/38:5)	Triacylglycerol(16:1/38:5)	0.0224	/	/	/	/	/	/			
TG(17:0/32:1)	Triacylglycerol(17:0/32:1)	0.0427	/	/	/	/	/	/			
TG(17:0/34:1)	Triacylglycerol(17:0/34:1)	0.0228	/	/	/	/	/	/			
TG(17:0/34:2)	Triacylglycerol(17:0/34:2)	0.0723	/	/	/	/	/	/			
TG(17:0/34:3)	Triacylglycerol(17:0/34:3)	0.0690	/	/	/	/	/	/			
TG(17:0/36:3)	Triacylglycerol(17:0/36:3)	0.0450	/	/	/	/	/	/			
TG(17:0/36:4)	Triacylglycerol(17:0/36:4)	0.0240	/	/	/	/	/	/			
TG(17:1/32:1)	Triacylglycerol(17:1/32:1)	0.0532	/	/	/	/	/	/			
TG(17:1/34:1)	Triacylglycerol(17:1/34:1)	0.0183	/	/	/	/	/	/			
TG(17:1/34:2)	Triacylglycerol(17:1/34:2)	0.0837	/	/	/	/	/	/			
TG(17:1/34:3)	Triacylglycerol(17:1/34:3)	0.0939	/	/	/	/	/	/			
TG(17:1/36:3)	Triacylglycerol(17:1/36:3)	0.0348	/	/	/	/	/	/			
TG(17:1/36:4)	Triacylglycerol(17:1/36:4)	0.0475	/	/	/	/	/	/			
TG(17:1/36:5)	Triacylglycerol(17:1/36:5)	0.0944	/	/	/	/	/	/			
TG(17:1/38:5)	Triacylglycerol(17:1/38:5)	0.0923	/	/	/	/	/	/			
TG(17:1/38:6)	Triacylglycerol(17:1/38:6)	0.0872	/	/	/	/	/	/			
TG(17:1/38:7)	Triacylglycerol(17:1/38:7)	0.0913	/	/	/	/	/	/			
TG(17:2/34:2)	Triacylglycerol(17:2/34:2)	0.0630	/	/	/	/	/	/			
TG(17:2/34:3)	Triacylglycerol(17:2/34:3)	0.0794	/	/	/	/	/	/			
TG(17:2/36:2)	Triacylglycerol(17:2/36:2)	0.0970	/	/	/	/	/	/			
TG(17:2/36:3)	Triacylglycerol(17:2/36:3)	0.0799	/	/	/	/	/	/			
TG(17:2/36:4)	Triacylglycerol(17:2/36:4)	0.0697	/	/	/	/	/	/			

TG(17:2/38:5)	Triacylglycerol(17:2/38:5)	0.0529	/	/	/	/	/	/			
TG(17:2/38:6)	Triacylglycerol(17:2/38:6)	0.0980	/	/	/	/	/	/			
TG(17:2/38:7)	Triacylglycerol(17:2/38:7)	0.0680	/	/	/	/	/	/			
TG(18:0/30:0)	Triacylglycerol(18:0/30:0)	0.0595	/	/	/	/	/	/			
TG(18:0/30:1)	Triacylglycerol(18:0/30:1)	0.0623	/	/	/	/	/	/			
TG(18:0/32:0)	Triacylglycerol(18:0/32:0)	0.0618	/	/	/	/	/	/			
TG(18:0/32:1)	Triacylglycerol(18:0/32:1)	0.0294	/	/	/	/	/	/			
TG(18:0/32:2)	Triacylglycerol(18:0/32:2)	0.0127	/	/	/	/	/	/			
TG(18:0/34:2)	Triacylglycerol(18:0/34:2)	0.166	/	/	/	/	/	/			
TG(18:0/34:3)	Triacylglycerol(18:0/34:3)	0.0448	/	/	/	/	/	/			
TG(18:0/36:1)	Triacylglycerol(18:0/36:1)	0.0452	/	/	/	/	/	/			
TG(18:0/36:2)	Triacylglycerol(18:0/36:2)	0.0580	/	/	/	/	/	/			
TG(18:0/36:3)	Triacylglycerol(18:0/36:3)	0.0799	/	/	/	/	/	/			
TG(18:0/36:4)	Triacylglycerol(18:0/36:4)	0.0586	/	/	/	/	/	/			
TG(18:0/36:5)	Triacylglycerol(18:0/36:5)	0.0614	/	/	/	/	/	/			
TG(18:0/38:6)	Triacylglycerol(18:0/38:6)	0.0514	/	/	/	/	/	/			
TG(18:0/38:7)	Triacylglycerol(18:0/38:7)	0.0397	/	/	/	/	/	/			
TG(18:1/26:0)	Triacylglycerol(18:1/26:0)	0.0146	/	/	/	/	/	/			
TG(18:1/28:1)	Triacylglycerol(18:1/28:1)	0.0282	/	/	/	/	/	/			
TG(18:1/30:0)	Triacylglycerol(18:1/30:0)	0.0580	/	/	/	/	/	/			
TG(18:1/30:1)	Triacylglycerol(18:1/30:1)	0.0599	/	/	/	/	/	/			
TG(18:1/30:2)	Triacylglycerol(18:1/30:2)	0.0126	/	/	/	/	/	/			
TG(18:1/31:0)	Triacylglycerol(18:1/31:0)	0.0571	/	/	/	/	/	/			
TG(18:1/32:0)	Triacylglycerol(18:1/32:0)	0.0680	/	/	/	/	/	/			
TG(18:1/32:1)	Triacylglycerol(18:1/32:1)	0.0477	/	/	/	/	/	/			
TG(18:1/32:2)	Triacylglycerol(18:1/32:2)	0.0338	/	/	/	/	/	/			
TG(18:1/32:3)	Triacylglycerol(18:1/32:3)	0.0108	/	/	/	/	/	/			
TG(18:1/33:0)	Triacylglycerol(18:1/33:0)	0.0172	/	/	/	/	/	/			
TG(18:1/33:1)	Triacylglycerol(18:1/33:1)	0.0286	/	/	/	/	/	/			
TG(18:1/33:2)	Triacylglycerol(18:1/33:2)	0.0840	/	/	/	/	/	/			
TG(18:1/33:3)	Triacylglycerol(18:1/33:3)	0.0907	/	/	/	/	/	/			

TG(18:1/34:1)	Triacylglycerol(18:1/34:1)	0.118	/	/	/	/	/	/			
TG(18:1/34:2)	Triacylglycerol(18:1/34:2)	0.0113	/	/	/	/	/	/			
TG(18:1/34:3)	Triacylglycerol(18:1/34:3)	0.0860	/	/	/	/	/	/			
TG(18:1/34:4)	Triacylglycerol(18:1/34:4)	0.0126	/	/	/	/	/	/			
TG(18:1/35:2)	Triacylglycerol(18:1/35:2)	0.0647	/	/	/	/	/	/			
TG(18:1/35:3)	Triacylglycerol(18:1/35:3)	0.0402	/	/	/	/	/	/			
TG(18:1/36:0)	Triacylglycerol(18:1/36:0)	0.0280	/	/	/	/	/	/			
TG(18:1/36:1)	Triacylglycerol(18:1/36:1)	0.0638	/	/	/	/	/	/			
TG(18:1/36:2)	Triacylglycerol(18:1/36:2)	0.0334	98.7	94.7	110	4.08	7.45	10.8	89.3	86.3	92.4
TG(18:1/36:3)	Triacylglycerol(18:1/36:3)	0.0884	/	/	/	/	/	/			
TG(18:1/36:4)	Triacylglycerol(18:1/36:4)	0.0681	/	/	/	/	/	/			
TG(18:1/36:5)	Triacylglycerol(18:1/36:5)	0.0988	/	/	/	/	/	/			
TG(18:1/36:6)	Triacylglycerol(18:1/36:6)	0.0380	/	/	/	/	/	/			
TG(18:1/38:5)	Triacylglycerol(18:1/38:5)	0.0135	/	/	/	/	/	/			
TG(18:1/38:6)	Triacylglycerol(18:1/38:6)	0.0170	/	/	/	/	/	/			
TG(18:1/38:7)	Triacylglycerol(18:1/38:7)	0.0310	/	/	/	/	/	/			
TG(18:2/28:0)	Triacylglycerol(18:2/28:0)	0.0160	/	/	/	/	/	/			
TG(18:2/30:0)	Triacylglycerol(18:2/30:0)	0.0166	/	/	/	/	/	/			
TG(18:2/30:1)	Triacylglycerol(18:2/30:1)	0.106	/	/	/	/	/	/			
TG(18:2/31:0)	Triacylglycerol(18:2/31:0)	0.0140	/	/	/	/	/	/			
TG(18:2/32:0)	Triacylglycerol(18:2/32:0)	0.0152	/	/	/	/	/	/			
TG(18:2/32:1)	Triacylglycerol(18:2/32:1)	0.0157	/	/	/	/	/	/			
TG(18:2/32:2)	Triacylglycerol(18:2/32:2)	0.0278	/	/	/	/	/	/			
TG(18:2/33:0)	Triacylglycerol(18:2/33:0)	0.0602	/	/	/	/	/	/			
TG(18:2/33:1)	Triacylglycerol(18:2/33:1)	0.0557	/	/	/	/	/	/			
TG(18:2/33:2)	Triacylglycerol(18:2/33:2)	0.0810	/	/	/	/	/	/			
TG(18:2/34:0)	Triacylglycerol(18:2/34:0)	0.102	/	/	/	/	/	/			
TG(18:2/34:1)	Triacylglycerol(18:2/34:1)	0.0154	/	/	/	/	/	/			
TG(18:2/34:2)	Triacylglycerol(18:2/34:2)	0.0185	/	/	/	/	/	/			
TG(18:2/34:3)	Triacylglycerol(18:2/34:3)	0.114	/	/	/	/	/	/			
TG(18:2/34:4)	Triacylglycerol(18:2/34:4)	0.0748	/	/	/	/	/	/			

TG(18:2/35:1)	Triacylglycerol(18:2/35:1)	0.0440	/	/	/	/	/	/			
TG(18:2/35:2)	Triacylglycerol(18:2/35:2)	0.0850	/	/	/	/	/	/			
TG(18:2/35:3)	Triacylglycerol(18:2/35:3)	0.157	/	/	/	/	/	/			
TG(18:2/36:0)	Triacylglycerol(18:2/36:0)	0.0643	/	/	/	/	/	/			
TG(18:2/36:1)	Triacylglycerol(18:2/36:1)	0.0738	/	/	/	/	/	/			
TG(18:2/36:2)	Triacylglycerol(18:2/36:2)	0.0503	/	/	/	/	/	/			
TG(18:2/36:3)	Triacylglycerol(18:2/36:3)	0.0191	/	/	/	/	/	/			
TG(18:2/36:4)	Triacylglycerol(18:2/36:4)	0.0133	/	/	/	/	/	/			
TG(18:2/36:5)	Triacylglycerol(18:2/36:5)	0.0418	/	/	/	/	/	/			
TG(18:2/38:4)	Triacylglycerol(18:2/38:4)	0.0294	/	/	/	/	/	/			
TG(18:2/38:5)	Triacylglycerol(18:2/38:5)	0.0801	/	/	/	/	/	/			
TG(18:2/38:6)	Triacylglycerol(18:2/38:6)	0.0199	/	/	/	/	/	/			
TG(18:3/30:0)	Triacylglycerol(18:3/30:0)	0.0450	/	/	/	/	/	/			
TG(18:3/32:0)	Triacylglycerol(18:3/32:0)	0.0474	/	/	/	/	/	/			
TG(18:3/32:1)	Triacylglycerol(18:3/32:1)	0.0536	/	/	/	/	/	/			
TG(18:3/33:2)	Triacylglycerol(18:3/33:2)	0.0970	/	/	/	/	/	/			
TG(18:3/34:0)	Triacylglycerol(18:3/34:0)	0.0219	/	/	/	/	/	/			
TG(18:3/34:1)	Triacylglycerol(18:3/34:1)	0.0111	/	/	/	/	/	/			
TG(18:3/34:2)	Triacylglycerol(18:3/34:2)	0.0615	/	/	/	/	/	/			
TG(18:3/34:3)	Triacylglycerol(18:3/34:3)	0.0628	/	/	/	/	/	/			
TG(18:3/35:2)	Triacylglycerol(18:3/35:2)	0.0735	/	/	/	/	/	/			
TG(18:3/36:1)	Triacylglycerol(18:3/36:1)	0.0171	/	/	/	/	/	/			
TG(18:3/36:2)	Triacylglycerol(18:3/36:2)	0.0947	/	/	/	/	/	/			
TG(18:3/36:3)	Triacylglycerol(18:3/36:3)	0.0959	/	/	/	/	/	/			
TG(18:3/36:4)	Triacylglycerol(18:3/36:4)	0.0820	/	/	/	/	/	/			
TG(18:3/38:5)	Triacylglycerol(18:3/38:5)	0.0515	/	/	/	/	/	/			
TG(18:3/38:6)	Triacylglycerol(18:3/38:6)	0.0446	/	/	/	/	/	/			
TG(20:0/32:3)	Triacylglycerol(20:0/32:3)	0.0624	/	/	/	/	/	/			
TG(20:0/32:4)	Triacylglycerol(20:0/32:4)	0.118	/	/	/	/	/	/			
TG(20:0/34:1)	Triacylglycerol(20:0/34:1)	0.104	/	/	/	/	/	/			
TG(20:1/24:3)	Triacylglycerol(20:1/24:3)	0.0544	/	/	/	/	/	/			

TG(20:1/26:1)	Triacylglycerol(20:1/26:1)	0.0529	/	/	/	/	/	/			
TG(20:1/30:1)	Triacylglycerol(20:1/30:1)	0.0333	/	/	/	/	/	/			
TG(20:1/31:0)	Triacylglycerol(20:1/31:0)	0.0566	/	/	/	/	/	/			
TG(20:1/32:1)	Triacylglycerol(20:1/32:1)	0.0561	/	/	/	/	/	/			
TG(20:1/32:2)	Triacylglycerol(20:1/32:2)	0.0790	/	/	/	/	/	/			
TG(20:1/32:3)	Triacylglycerol(20:1/32:3)	0.0297	/	/	/	/	/	/			
TG(20:1/34:0)	Triacylglycerol(20:1/34:0)	0.0279	/	/	/	/	/	/			
TG(20:1/34:1)	Triacylglycerol(20:1/34:1)	0.0409	/	/	/	/	/	/			
TG(20:1/34:2)	Triacylglycerol(20:1/34:2)	0.0149	/	/	/	/	/	/			
TG(20:1/34:3)	Triacylglycerol(20:1/34:3)	0.0641	/	/	/	/	/	/			
TG(20:2/32:0)	Triacylglycerol(20:2/32:0)	0.112	/	/	/	/	/	/			
TG(20:2/32:1)	Triacylglycerol(20:2/32:1)	0.0841	/	/	/	/	/	/			
TG(20:2/34:1)	Triacylglycerol(20:2/34:1)	0.0432	/	/	/	/	/	/			
TG(20:2/34:2)	Triacylglycerol(20:2/34:2)	0.0865	/	/	/	/	/	/			
TG(20:2/34:3)	Triacylglycerol(20:2/34:3)	0.0561	/	/	/	/	/	/			
TG(20:2/34:4)	Triacylglycerol(20:2/34:4)	0.130	/	/	/	/	/	/			
TG(20:2/36:5)	Triacylglycerol(20:2/36:5)	0.120	/	/	/	/	/	/			
TG(20:3/32:0)	Triacylglycerol(20:3/32:0)	0.113	/	/	/	/	/	/			
TG(20:3/32:1)	Triacylglycerol(20:3/32:1)	0.0884	/	/	/	/	/	/			
TG(20:3/32:2)	Triacylglycerol(20:3/32:2)	0.0174	/	/	/	/	/	/			
TG(20:3/34:0)	Triacylglycerol(20:3/34:0)	0.0330	/	/	/	/	/	/			
TG(20:3/34:1)	Triacylglycerol(20:3/34:1)	0.0707	/	/	/	/	/	/			
TG(20:3/34:2)	Triacylglycerol(20:3/34:2)	0.0447	/	/	/	/	/	/			
TG(20:3/34:3)	Triacylglycerol(20:3/34:3)	0.0262	/	/	/	/	/	/			
TG(20:3/36:3)	Triacylglycerol(20:3/36:3)	0.0310	/	/	/	/	/	/			
TG(20:3/36:4)	Triacylglycerol(20:3/36:4)	0.0128	/	/	/	/	/	/			
TG(20:3/36:5)	Triacylglycerol(20:3/36:5)	0.0330	/	/	/	/	/	/			
TG(20:4/30:0)	Triacylglycerol(20:4/30:0)	0.0102	/	/	/	/	/	/			
TG(20:4/32:0)	Triacylglycerol(20:4/32:0)	0.0347	/	/	/	/	/	/			
TG(20:4/32:1)	Triacylglycerol(20:4/32:1)	0.0652	/	/	/	/	/	/			
TG(20:4/32:2)	Triacylglycerol(20:4/32:2)	0.0124	/	/	/	/	/	/			

TG(20:4/33:2)	Triacylglycerol(20:4/33:2)	0.0424	/	/	/	/	/	/			
TG(20:4/34:0)	Triacylglycerol(20:4/34:0)	0.0353	/	/	/	/	/	/			
TG(20:4/34:1)	Triacylglycerol(20:4/34:1)	0.0303	/	/	/	/	/	/			
TG(20:4/34:2)	Triacylglycerol(20:4/34:2)	0.0707	/	/	/	/	/	/			
TG(20:4/34:3)	Triacylglycerol(20:4/34:3)	0.0598	/	/	/	/	/	/			
TG(20:4/35:3)	Triacylglycerol(20:4/35:3)	0.0667	/	/	/	/	/	/			
TG(20:4/36:2)	Triacylglycerol(20:4/36:2)	0.0223	/	/	/	/	/	/			
TG(20:4/36:3)	Triacylglycerol(20:4/36:3)	0.0658	/	/	/	/	/	/			
TG(20:4/36:4)	Triacylglycerol(20:4/36:4)	0.0128	/	/	/	/	/	/			
TG(20:4/36:5)	Triacylglycerol(20:4/36:5)	0.0340	/	/	/	/	/	/			
TG(20:5/34:0)	Triacylglycerol(20:5/34:0)	0.0713	/	/	/	/	/	/			
TG(20:5/34:1)	Triacylglycerol(20:5/34:1)	0.0784	/	/	/	/	/	/			
TG(20:5/34:2)	Triacylglycerol(20:5/34:2)	0.0248	/	/	/	/	/	/			
TG(20:5/36:2)	Triacylglycerol(20:5/36:2)	0.0286	/	/	/	/	/	/			
TG(20:5/36:3)	Triacylglycerol(20:5/36:3)	0.0301	/	/	/	/	/	/			
TG(22:0/32:4)	Triacylglycerol(22:0/32:4)	0.0232	/	/	/	/	/	/			
TG(22:1/32:5)	Triacylglycerol(22:1/32:5)	0.0888	/	/	/	/	/	/			
TG(22:2/32:4)	Triacylglycerol(22:2/32:4)	0.0457	/	/	/	/	/	/			
TG(22:3/30:2)	Triacylglycerol(22:3/30:2)	0.0523	/	/	/	/	/	/			
TG(22:4/32:0)	Triacylglycerol(22:4/32:0)	0.0326	/	/	/	/	/	/			
TG(22:4/32:2)	Triacylglycerol(22:4/32:2)	0.0969	/	/	/	/	/	/			
TG(22:4/34:2)	Triacylglycerol(22:4/34:2)	0.0391	/	/	/	/	/	/			
TG(22:5/32:0)	Triacylglycerol(22:5/32:0)	0.0217	/	/	/	/	/	/			
TG(22:5/32:1)	Triacylglycerol(22:5/32:1)	0.106	/	/	/	/	/	/			
TG(22:5/34:1)	Triacylglycerol(22:5/34:1)	0.0314	/	/	/	/	/	/			
TG(22:5/34:2)	Triacylglycerol(22:5/34:2)	0.0608	/	/	/	/	/	/			
TG(22:5/34:3)	Triacylglycerol(22:5/34:3)	0.0765	/	/	/	/	/	/			
TG(22:6/32:0)	Triacylglycerol(22:6/32:0)	0.0626	/	/	/	/	/	/			
TG(22:6/32:1)	Triacylglycerol(22:6/32:1)	0.0230	/	/	/	/	/	/			
TG(22:6/34:1)	Triacylglycerol(22:6/34:1)	0.0245	/	/	/	/	/	/			
TG(22:6/34:2)	Triacylglycerol(22:6/34:2)	0.0144	/	/	/	/	/	/			

TG(22:6/34:3)	Triacylglycerol(22:6/34:3)	0.0765	/	/	/	/	/	/			

Supplementary Table S7. Mean Concentrations and Standard Deviations of Detected Metabolites for the Two Time Points in the Intervention Study

Analyte	V1 mean ± SD (μM)	V2 mean ± SD (μM)
1-Methylnicotinamide	0.103 ± 0.0591	0.109 ± 0.0580
1,3-Diaminopropane	0.610 ± 0.201	0.559 ± 0.163
2-Hydroxy-2-methylbutyric acid	0.169 ± 0.122	0.215 ± 0.184
2-Hydroxy-3-methylvaleric acid	0.687 ± 0.375	0.649 ± 0.374
2-Hydroxybutyric acid	31.4 ± 14.2	33.2 ± 21.1
2-hydroxyglutaric acid	0.412 ± 0.209	0.388 ± 0.160
2-Hydroxyisobutyric acid	1.50 ± 1.19	2.40 ± 4.29
2-Hydroxyisovaleric acid	6.75 ± 4.16	7.01 ± 3.91
2-Hydroxyphenylacetic acid	7.59 ± 5.52	7.82 ± 5.21
2-Oxoisocaproic acid	67.2 ± 24.8	64.6 ± 34.9
2,5-Furandicarboxylic acid	0.105 ± 0.0911	0.0889 ± 0.0934
3-(3-Hydroxyphenyl)-3-hydroxypropanoic acid	0.156 ± 0.142	0.137 ± 0.105
3-Aminoisobutyric acid	1.99 ± 1.26	2.85 ± 2.72
3-Carboxy-4-methyl-5-propyl-2-furanpropionic acid	10.4 ± 8.37	9.74 ± 6.61
3-Deoxyglucosone	0.0508 ± 0.0196	0.0530 ± 0.0259
3-Hydroxybutyric acid	52.1 ± 47.0	48.0 ± 29.3
3-Hydroxyisobutyric acid	10.8 ± 3.74	10.3 ± 3.44
3-Hydroxyisovaleric acid	3.60 ± 4.86	2.50 ± 1.07
3-Hydroxyphenylacetic acid	0.110 ± 0.0834	0.157 ± 0.123
3-Indoleacetic acid	1.45 ± 0.959	1.55 ± 1.04
3-Methyladipic acid	0.0410 ± 0.0195	0.0562 ± 0.0397
4-Hydroxyphenylacetic acid	0.344 ± 0.154	0.274 ± 0.163
4-Hydroxyphenylpyruvic acid	0.806 ± 0.465	0.802 ± 0.444
5-Hydroxyindoleacetic acid	0.0397 ± 0.0263	0.0518 ± 0.0576
5-Oxoproline	53.0 ± 23.8	52.5 ± 23.3
7-Methylguanine	0.110 ± 0.0587	0.108 ± 0.0492
Acetoacetic acid	31.0 ± 23.6	32.6 ± 15.5

Analyte	V1 mean ± SD (μM)	V2 mean ± SD (μM)
Adenosine	0.133 ± 0.123	0.166 ± 0.143
Alanine	444 ± 208	422 ± 145
Allantoin	2.33 ± 0.632	2.10 ± 0.738
alpha-Aminoadipic acid	1.42 ± 0.564	1.45 ± 0.591
alpha-Aminobutyric acid	21.4 ± 7.28	22.6 ± 10.5
alpha-Ketoglutaric acid	22.4 ± 8.06	22.0 ± 10.5
alpha-Ketoisovaleric acid	18.7 ± 4.92	18.2 ± 9.51
Arginine	109 ± 53.3	98.2 ± 28.0
Argininic acid	27.0 ± 21.4	27.5 ± 22.4
Asparagine	45.8 ± 14.8	44.3 ± 11.8
Asymmetric dimethylarginine	0.674 ± 0.235	0.639 ± 0.144
Asymmetric dimethylarginine + Symmetric dimethylarginine	1.95 ± 0.898	1.85 ± 0.464
beta-Alanine	1.57 ± 0.431	1.37 ± 0.449
Betaine	141 ± 52.7	134 ± 39.2
C0	60.4 ± 36.9	56.6 ± 29.8
C2	7.49 ± 3.92	7.71 ± 2.76
C3	0.450 ± 0.254	0.421 ± 0.203
C4	0.178 ± 0.128	0.160 ± 0.0968
CE(14:0)	74.7 ± 47.8	56.0 ± 20.6
CE(14:1)	6.52 ± 4.43	5.06 ± 2.90
CE(15:0)	19.8 ± 10.3	17.6 ± 7.31
CE(16:0)	811 ± 343	742 ± 189
CE(16:1)	688 ± 343	581 ± 277
CE(17:0)	14.8 ± 8.00	12.3 ± 3.99
CE(17:1)	44.6 ± 21.1	38.6 ± 12.7
CE(18:0)	132 ± 64.9	125 ± 41.9
CE(18:1)	5480 ± 2550	5140 ± 1870
CE(18:2)	18700 ± 9170	17500 ± 6380
CE(18:3)	736 ± 431	623 ± 306

Analyte	V1 mean ± SD (μM)	V2 mean ± SD (μM)
CE(20:3)	520 ± 266	474 ± 180
CE(20:4)	5100 ± 2690	4490 ± 1590
CE(20:5)	965 ± 715	887 ± 602
CE(22:5)	55.5 ± 29.7	47.8 ± 17.8
CE(22:6)	650 ± 340	580 ± 245
Cer(d16:1/22:0)	0.0658 ± 0.0309	0.0601 ± 0.0246
Cer(d16:1/24:0)	0.112 ± 0.0491	0.0931 ± 0.0381
Cer(d18:1/16:0)	0.673 ± 0.258	0.698 ± 0.218
Cer(d18:1/18:0)	0.118 ± 0.0576	0.128 ± 0.0525
Cer(d18:1/22:0)	0.737 ± 0.331	0.686 ± 0.253
Cer(d18:1/23:0)	0.595 ± 0.240	0.608 ± 0.218
Cer(d18:1/24:0)	2.08 ± 0.882	1.95 ± 0.662
Cer(d18:1/24:1)	1.31 ± 0.546	1.36 ± 0.376
Cer(d18:2/22:0)	0.150 ± 0.0727	0.126 ± 0.0631
Cer(d18:2/24:0)	0.416 ± 0.198	0.350 ± 0.163
Cer(d18:2/24:1)	0.250 ± 0.110	0.242 ± 0.0922
Choline	12.4 ± 4.36	11.9 ± 2.54
cis-Aconitic acid	1.24 ± 0.466	1.23 ± 0.348
Citric acid	67.3 ± 18.4	63.8 ± 12.6
Citrulline	52.9 ± 29.1	50.4 ± 20.4
Creatine	26.1 ± 17.9	21.4 ± 13.4
Creatinine	99.7 ± 46.4	93.5 ± 32.4
Cystathionine	0.858 ± 1.00	0.917 ± 1.30
DG(16:0_18:2)	0.813 ± 0.742	0.728 ± 0.513
DG(16:1_18:2)	0.213 ± 0.176	0.222 ± 0.159
DG(18:1_18:1)	9.04 ± 5.52	9.51 ± 3.92
DG(18:1_18:2)	2.92 ± 1.81	3.00 ± 1.55
DG(18:2_18:2)	1.19 ± 1.22	1.06 ± 0.876
Dimethylglycine	5.96 ± 1.95	6.42 ± 2.26

Analyte	V1 mean ± SD (μM)	V2 mean ± SD (μM)
Ethanolamine	7.19 ± 1.81	7.20 ± 1.61
Ethylmalonic acid	0.252 ± 0.128	0.258 ± 0.212
Fumaric acid	0.786 ± 0.274	0.834 ± 0.277
gamma-Aminobutyric acid	0.921 ± 0.287	0.978 ± 0.426
Glutamic acid	63.1 ± 39.9	61.5 ± 34.2
Glutamine	700 ± 239	683 ± 138
Glutaric acid	0.144 ± 0.103	0.136 ± 0.165
Glyceric acid	4.60 ± 1.91	4.36 ± 1.45
Glycine	334 ± 176	310 ± 125
Guanidinopropionic acid	22.5 ± 13.8	20.7 ± 14.7
Guanidoacetic acid	3.76 ± 1.85	3.64 ± 1.07
LacCer(d18:1/14:0)	0.0838 ± 0.0391	0.0756 ± 0.0150
LacCer(d18:1/16:0)	2.30 ± 0.783	2.23 ± 0.618
LacCer(d18:1/18:0)	0.108 ± 0.0347	0.0976 ± 0.0285
LacCer(d18:1/20:0)	0.0481 ± 0.0177	0.0453 ± 0.0155
LacCer(d18:1/22:0)	0.146 ± 0.0509	0.142 ± 0.0449
LacCer(d18:1/24:0)	0.179 ± 0.0573	0.174 ± 0.0586
LacCer(d18:1/24:1)	0.564 ± 0.215	0.593 ± 0.185
Trihexosylceramide(d18:1/22:0)	0.0523 ± 0.0145	0.0499 ± 0.0201
Trihexosylceramide(d18:1/16:0)	0.356 ± 0.0975	0.371 ± 0.0986
Trihexosylceramide(d18:1/18:0)	0.0491 ± 0.0210	0.0516 ± 0.0230
Trihexosylceramide(d18:1/24:1)	0.135 ± 0.0476	0.142 ± 0.0493
GlcCer(d16:1/22:0)	0.0714 ± 0.0267	0.0704 ± 0.0287
GlcCer(d18:1/16:0)	0.669 ± 0.320	0.671 ± 0.274
GlcCer(d18:1/18:0)	0.117 ± 0.0664	0.108 ± 0.0573
GlcCer(d18:1/20:0)	0.127 ± 0.0708	0.131 ± 0.0691
GlcCer(d18:1/22:0)	1.01 ± 0.474	1.00 ± 0.428
GlcCer(d18:1/23:0)	0.546 ± 0.234	0.533 ± 0.194
GlcCer(d18:1/24:0)	1.49 ± 0.554	1.40 ± 0.509

Analyte	V1 mean ± SD (μM)	V2 mean ± SD (μM)
GlcCer(d18:1/24:1)	1.36 ± 0.622	1.37 ± 0.518
GlcCer(d18:2/20:0)	0.0383 ± 0.0223	0.0275 ± 0.0194
GlcCer(d18:2/22:0)	0.171 ± 0.104	0.147 ± 0.0809
GlcCer(d18:2/23:0)	0.0813 ± 0.0397	0.0785 ± 0.0366
GlcCer(d18:2/24:0)	0.232 ± 0.0945	0.225 ± 0.0993
Hexose	5990 ± 2140	5470 ± 1150
Hippuric acid	6.68 ± 5.67	8.58 ± 7.16
Histidine	99.8 ± 36.0	94.3 ± 25.2
Homoarginine	0.806 ± 0.462	0.724 ± 0.484
Hypoxanthine	2.30 ± 1.23	3.14 ± 2.38
Indole	13.1 ± 6.71	11.6 ± 3.91
Indole-3-propionic acid	0.841 ± 0.466	0.807 ± 0.615
Indolelactic acid	0.721 ± 0.512	0.655 ± 0.640
Indoxyl sulfate	5.50 ± 4.48	4.26 ± 4.99
Isocitric acid	2.62 ± 1.02	2.63 ± 1.01
Isoleucine	71.6 ± 23.4	65.3 ± 17.6
Kynurenic acid	0.137 ± 0.104	0.131 ± 0.0699
Kynurenine	3.24 ± 1.73	3.19 ± 1.41
Lactic acid	1080 ± 402	1190 ± 480
Leucine	144 ± 45.1	133 ± 33.7
Lysine	292 ± 92.4	269 ± 48.7
LysoPC a C14:0	1.15 ± 0.622	0.920 ± 0.373
LysoPC a C16:0	51.3 ± 24.5	46.6 ± 16.5
LysoPC a C16:1	1.29 ± 0.551	1.18 ± 0.442
LysoPC a C17:0	0.855 ± 0.425	0.791 ± 0.306
LysoPC a C18:0	17.1 ± 7.62	16.5 ± 6.75
LysoPC a C18:1	18.1 ± 8.97	17.5 ± 6.62
LysoPC a C18:2	20.8 ± 10.4	17.6 ± 5.86
LysoPC a C20:4	4.50 ± 3.48	3.59 ± 1.71

Analyte	V1 mean ± SD (μM)	V2 mean ± SD (μM)
Maleic acid	0.184 ± 0.127	0.147 ± 0.159
Malic acid	2.95 ± 1.03	2.92 ± 0.910
Methionine	32.5 ± 11.1	29.8 ± 7.36
Methionine sulfoxide	1.10 ± 0.471	1.01 ± 0.441
Methylhistidine	38.6 ± 37.2	32.1 ± 33.9
Methylmalonic acid	0.255 ± 0.305	0.201 ± 0.153
N-Acetyl-Alanine	1.65 ± 0.473	1.60 ± 0.463
N-Acetyl-Arginine	0.583 ± 0.268	0.672 ± 0.350
N-Acetyl-Asparagine	0.236 ± 0.110	0.210 ± 0.0867
N-Acetyl-Aspartic acid	0.197 ± 0.166	0.140 ± 0.0757
N-Acetyl-Glutamic acid	0.0727 ± 0.0308	0.0686 ± 0.0240
N-Acetyl-Glycine	4.29 ± 2.51	3.97 ± 1.68
N-Acetyl-Histidine	0.867 ± 0.679	0.726 ± 0.451
N-Acetyl-Methionine	0.370 ± 0.409	0.362 ± 0.379
N-Acetyl-Proline	0.0364 ± 0.0187	0.0346 ± 0.0206
N-Acetyl-Serine	0.967 ± 0.296	0.888 ± 0.229
N1-Acetyl-Lysine + N6-Acetyl-Lysine	1.35 ± 0.399	1.29 ± 0.653
N1-Acetylspermidine	0.123 ± 0.0938	0.122 ± 0.0708
N2-Acetyl-Ornithine	1.34 ± 1.27	1.32 ± 1.26
Nudifloramide	2.15 ± 0.987	2.13 ± 0.949
Ornithine	85.2 ± 27.3	88.1 ± 25.2
p-Cresol sulfate	26.2 ± 14.1	25.4 ± 17.7
p-Hydroxyhippuric acid	0.146 ± 0.0792	0.123 ± 0.0719
PC aa C30:0	4.60 ± 1.57	4.05 ± 1.02
PC aa C32:0	14.7 ± 4.79	13.2 ± 3.12
PC aa C32:1	19.8 ± 8.15	16.8 ± 5.44
PC aa C32:2	3.25 ± 1.44	2.95 ± 0.945
PC aa C32:3	0.395 ± 0.164	0.367 ± 0.130
PC aa C34:1	289 ± 112	273 ± 102

Analyte	V1 mean ± SD (μM)	V2 mean ± SD (μM)
PC aa C34:2	415 ± 140	379 ± 101
PC aa C34:3	11.4 ± 4.19	9.72 ± 3.80
PC aa C34:4	1.36 ± 0.710	1.03 ± 0.475
PC aa C36:0	7.92 ± 2.66	7.78 ± 3.31
PC aa C36:1	79.4 ± 27.7	78.6 ± 31.5
PC aa C36:2	277 ± 96.8	272 ± 85.9
PC aa C36:3	161 ± 58.2	156 ± 52.1
PC aa C36:4	202 ± 102	176 ± 60.1
PC aa C36:5	24.8 ± 15.9	23.1 ± 13.3
PC aa C36:6	0.967 ± 0.574	0.818 ± 0.460
PC aa C38:0	4.38 ± 2.04	4.31 ± 2.07
PC aa C38:1	9.94 ± 3.55	9.81 ± 3.12
PC aa C38:3	67.2 ± 30.2	63.9 ± 26.6
PC aa C38:4	135 ± 73.3	123 ± 50.6
PC aa C38:5	63.5 ± 33.3	58.3 ± 22.7
PC aa C38:6	96.4 ± 55.6	86.4 ± 39.9
PC aa C40:1	0.397 ± 0.135	0.387 ± 0.127
PC aa C40:2	0.537 ± 0.170	0.509 ± 0.156
PC aa C40:3	0.854 ± 0.330	0.811 ± 0.302
PC aa C40:4	3.71 ± 1.73	3.29 ± 1.42
PC aa C40:5	11.2 ± 5.98	10.1 ± 5.17
PC aa C40:6	33.8 ± 20.4	31.0 ± 18.0
PC aa C42:0	0.545 ± 0.244	0.504 ± 0.189
PC aa C42:1	0.309 ± 0.118	0.279 ± 0.0897
PC aa C42:2	0.219 ± 0.0845	0.200 ± 0.0627
PC aa C42:4	0.201 ± 0.0767	0.175 ± 0.0489
PC aa C42:5	0.295 ± 0.125	0.266 ± 0.0897
PC aa C42:6	0.382 ± 0.193	0.359 ± 0.191
PC ae C30:0	0.180 ± 0.0967	0.162 ± 0.0796

Analyte	V1 mean ± SD (μM)	V2 mean ± SD (μM)
PC ae C30:1	1.84 ± 0.675	1.75 ± 0.537
PC ae C30:2	0.0808 ± 0.0321	0.0733 ± 0.0269
PC ae C32:1	3.32 ± 1.01	3.13 ± 0.714
PC ae C32:2	0.647 ± 0.218	0.637 ± 0.249
PC ae C34:0	1.88 ± 0.576	1.68 ± 0.320
PC ae C34:1	10.1 ± 3.37	9.51 ± 2.49
PC ae C34:2	10.1 ± 2.78	9.13 ± 2.54
PC ae C34:3	6.02 ± 1.98	5.54 ± 1.86
PC ae C36:0	1.50 ± 0.452	1.48 ± 0.376
PC ae C36:1	8.19 ± 2.92	7.82 ± 2.07
PC ae C36:2	12.3 ± 4.07	11.8 ± 3.12
PC ae C36:3	7.94 ± 2.32	7.42 ± 2.21
PC ae C36:4	15.0 ± 5.04	13.3 ± 4.11
PC ae C36:5	10.7 ± 3.31	9.92 ± 3.55
PC ae C38:0	2.31 ± 1.06	2.18 ± 1.11
PC ae C38:1	5.38 ± 1.78	5.43 ± 1.61
PC ae C38:2	6.14 ± 2.04	6.14 ± 1.96
PC ae C38:3	4.28 ± 1.63	4.09 ± 1.19
PC ae C38:4	12.6 ± 5.39	11.6 ± 3.43
PC ae C38:5	19.6 ± 7.74	18.0 ± 5.59
PC ae C38:6	8.14 ± 3.20	7.59 ± 3.32
PC ae C40:1	1.99 ± 0.776	1.83 ± 0.648
PC ae C40:2	1.53 ± 0.593	1.53 ± 0.400
PC ae C40:3	1.12 ± 0.336	1.09 ± 0.221
PC ae C40:4	2.32 ± 0.841	2.10 ± 0.502
PC ae C40:5	3.66 ± 1.45	3.42 ± 0.983
PC ae C40:6	5.15 ± 2.54	4.79 ± 2.09
PC ae C42:0	0.458 ± 0.321	0.440 ± 0.244
PC ae C42:1	0.362 ± 0.149	0.331 ± 0.129

Analyte	V1 mean $\pm$ SD (μM)	V2 mean $\pm$ SD (μM)
PC ae C42:2	0.559 $\pm$ 0.198	0.554 $\pm$ 0.200
PC ae C42:3	0.807 $\pm$ 0.272	0.744 $\pm$ 0.204
PC ae C42:4	0.962 $\pm$ 0.302	0.878 $\pm$ 0.189
PC ae C42:5	1.71 $\pm$ 0.669	1.61 $\pm$ 0.425
PC ae C44:4	0.479 $\pm$ 0.164	0.440 $\pm$ 0.100
PC ae C44:5	1.67 $\pm$ 0.615	1.53 $\pm$ 0.379
PC ae C44:6	1.11 $\pm$ 0.472	0.991 $\pm$ 0.286
Phenylacetic acid	0.711 $\pm$ 0.370	0.719 $\pm$ 0.349
Phenylalanine	75.9 $\pm$ 21.1	69.0 $\pm$ 12.6
Pipecolic acid	2.84 $\pm$ 1.29	2.93 $\pm$ 1.30
Proline	234 $\pm$ 138	214 $\pm$ 103
Pyruvic acid	64.7 $\pm$ 22.0	75.3 $\pm$ 37.0
Quinolinic acid	0.425 $\pm$ 0.357	0.418 $\pm$ 0.292
Sarcosine	3.51 $\pm$ 0.889	3.26 $\pm$ 0.962
Serine	142 $\pm$ 59.8	143 $\pm$ 35.4
SM C16:0	108 $\pm$ 35.7	107 $\pm$ 26.4
SM C16:1	15.0 $\pm$ 6.12	14.5 $\pm$ 4.65
SM C18:0	21.5 $\pm$ 9.08	20.9 $\pm$ 6.11
SM C18:1	9.95 $\pm$ 4.51	9.62 $\pm$ 3.38
SM C20:2	0.425 $\pm$ 0.210	0.358 $\pm$ 0.134
SM C24:0	23.0 $\pm$ 8.01	23.1 $\pm$ 6.79
SM C24:1	72.2 $\pm$ 26.7	71.6 $\pm$ 20.8
SM C26:0	0.306 $\pm$ 0.0837	0.311 $\pm$ 0.0801
SM C26:1	0.622 $\pm$ 0.207	0.640 $\pm$ 0.176
SMOH C14:1	4.67 $\pm$ 1.66	4.51 $\pm$ 1.30
SMOH C16:1	2.83 $\pm$ 1.02	2.77 $\pm$ 0.748
SMOH C22:1	12.0 $\pm$ 3.88	12.3 $\pm$ 3.40
SMOH C22:2	10.4 $\pm$ 3.56	10.4 $\pm$ 3.00
SMOH C24:1	1.63 $\pm$ 0.508	1.69 $\pm$ 0.416

Analyte	V1 mean ± SD (μM)	V2 mean ± SD (μM)
Succinic acid	3.64 ± 1.00	3.78 ± 0.986
Tartaric acid	0.317 ± 0.314	0.482 ± 1.00
Taurine	77.5 ± 31.4	76.7 ± 27.1
TG(14:0/32:2)	0.373 ± 0.422	0.378 ± 0.611
TG(14:0/34:0)	0.519 ± 0.627	0.358 ± 0.367
TG(14:0/34:1)	5.13 ± 5.69	3.52 ± 3.72
TG(14:0/34:2)	3.30 ± 3.28	2.61 ± 3.26
TG(14:0/34:3)	0.694 ± 0.628	0.563 ± 0.721
TG(14:0/36:1)	1.51 ± 1.42	1.31 ± 1.07
TG(14:0/36:2)	10.2 ± 9.27	9.77 ± 7.35
TG(14:0/36:3)	7.92 ± 7.27	7.41 ± 8.36
TG(14:0/36:4)	2.18 ± 2.30	1.89 ± 2.78
TG(14:0/38:4)	0.217 ± 0.195	0.183 ± 0.224
TG(14:0/38:5)	0.195 ± 0.162	0.175 ± 0.211
TG(16:0/30:2)	0.478 ± 0.542	0.407 ± 0.726
TG(16:0/32:1)	8.25 ± 9.64	5.37 ± 5.46
TG(16:0/32:2)	3.37 ± 3.59	2.62 ± 3.40
TG(16:0/32:3)	0.474 ± 0.393	0.418 ± 0.567
TG(16:0/33:1)	0.998 ± 0.983	0.761 ± 0.717
TG(16:0/34:0)	3.17 ± 3.24	2.33 ± 1.59
TG(16:0/34:1)	33.3 ± 32.5	25.3 ± 16.5
TG(16:0/34:2)	29.5 ± 27.1	23.8 ± 17.8
TG(16:0/34:3)	7.15 ± 6.14	5.59 ± 4.30
TG(16:0/34:4)	0.656 ± 0.526	0.536 ± 0.483
TG(16:0/35:1)	0.776 ± 0.754	0.632 ± 0.481
TG(16:0/35:2)	1.58 ± 1.34	1.36 ± 0.929
TG(16:0/35:3)	0.577 ± 0.508	0.493 ± 0.370
TG(16:0/36:2)	119 ± 87.8	120 ± 67.8
TG(16:0/36:3)	95.4 ± 76.4	87.0 ± 54.6

Analyte	V1 mean ± SD (μM)	V2 mean ± SD (μM)
TG(16:0/36:4)	26.5 ± 27.0	22.3 ± 18.9
TG(16:0/36:5)	2.28 ± 1.75	2.12 ± 1.87
TG(16:0/38:1)	0.212 ± 0.169	0.188 ± 0.149
TG(16:0/38:2)	1.19 ± 0.981	1.11 ± 0.736
TG(16:0/38:3)	1.83 ± 1.58	1.69 ± 1.27
TG(16:0/38:4)	2.46 ± 2.19	2.09 ± 1.80
TG(16:0/38:5)	2.66 ± 2.22	2.22 ± 1.82
TG(16:0/38:6)	1.49 ± 1.24	1.23 ± 0.986
TG(16:0/38:7)	0.357 ± 0.315	0.305 ± 0.262
TG(16:0/40:6)	1.50 ± 1.10	1.32 ± 0.821
TG(16:0/40:7)	1.94 ± 1.33	1.89 ± 1.31
TG(16:0/40:8)	0.758 ± 0.596	0.669 ± 0.490
TG(16:1/32:0)	1.31 ± 1.75	0.722 ± 0.631
TG(16:1/32:1)	2.37 ± 2.30	1.91 ± 1.72
TG(16:1/34:0)	1.71 ± 1.65	1.29 ± 0.895
TG(16:1/34:1)	14.8 ± 13.3	13.0 ± 9.84
TG(16:1/34:2)	8.60 ± 6.87	7.75 ± 5.35
TG(16:1/34:3)	1.58 ± 1.20	1.43 ± 0.995
TG(16:1/36:1)	2.94 ± 2.08	3.20 ± 1.60
TG(16:1/36:2)	20.4 ± 14.3	23.6 ± 11.8
TG(16:1/36:3)	15.2 ± 11.7	15.4 ± 9.05
TG(16:1/36:4)	4.02 ± 3.90	3.74 ± 2.85
TG(16:1/36:5)	0.437 ± 0.299	0.415 ± 0.289
TG(16:1/38:3)	0.345 ± 0.282	0.364 ± 0.236
TG(16:1/38:4)	0.474 ± 0.366	0.429 ± 0.359
TG(16:1/38:5)	0.504 ± 0.347	0.418 ± 0.292
TG(17:0/34:1)	0.646 ± 0.572	0.546 ± 0.411
TG(17:0/34:2)	0.551 ± 0.475	0.442 ± 0.338
TG(17:0/36:3)	1.43 ± 1.09	1.41 ± 0.954

Analyte	V1 mean ± SD (μM)	V2 mean ± SD (μM)
TG(17:0/36:4)	0.457 ± 0.423	0.381 ± 0.371
TG(17:1/34:1)	1.22 ± 1.06	1.07 ± 0.675
TG(17:1/34:2)	0.699 ± 0.541	0.636 ± 0.495
TG(17:1/34:3)	0.131 ± 0.0839	0.140 ± 0.103
TG(17:1/36:3)	0.881 ± 0.563	0.848 ± 0.518
TG(17:1/36:4)	0.261 ± 0.260	0.243 ± 0.202
TG(18:0/32:0)	0.745 ± 0.776	0.557 ± 0.387
TG(18:0/32:1)	1.79 ± 1.85	1.38 ± 1.06
TG(18:0/32:2)	0.562 ± 0.495	0.495 ± 0.512
TG(18:0/34:2)	5.69 ± 4.87	4.93 ± 3.09
TG(18:0/34:3)	1.03 ± 0.832	0.912 ± 0.564
TG(18:0/36:1)	1.42 ± 1.16	1.46 ± 0.763
TG(18:0/36:2)	9.32 ± 6.50	10.2 ± 4.54
TG(18:0/36:3)	9.12 ± 7.26	9.22 ± 5.21
TG(18:0/36:4)	3.22 ± 3.60	3.00 ± 2.54
TG(18:0/36:5)	0.382 ± 0.260	0.348 ± 0.240
TG(18:0/38:6)	0.539 ± 0.406	0.539 ± 0.297
TG(18:0/38:7)	0.149 ± 0.101	0.133 ± 0.0761
TG(18:1/26:0)	0.423 ± 0.596	0.268 ± 0.429
TG(18:1/28:1)	0.767 ± 0.898	0.537 ± 0.667
TG(18:1/30:0)	6.40 ± 6.65	4.66 ± 5.11
TG(18:1/30:1)	6.00 ± 8.46	4.48 ± 5.38
TG(18:1/30:2)	1.50 ± 2.34	1.21 ± 2.17
TG(18:1/32:0)	20.4 ± 19.0	16.2 ± 10.8
TG(18:1/32:1)	35.0 ± 29.2	32.5 ± 23.1
TG(18:1/32:2)	10.1 ± 8.50	9.38 ± 8.82
TG(18:1/32:3)	0.856 ± 0.632	0.857 ± 0.758
TG(18:1/33:0)	1.01 ± 0.826	0.834 ± 0.514
TG(18:1/33:1)	4.15 ± 2.95	4.11 ± 2.57

Analyte	V1 mean ± SD (μM)	V2 mean ± SD (μM)
TG(18:1/33:2)	1.54 ± 1.09	1.53 ± 1.11
TG(18:1/34:1)	205 ± 154	210 ± 114
TG(18:1/34:2)	114 ± 84.3	112 ± 61.4
TG(18:1/34:3)	17.5 ± 12.9	17.7 ± 10.3
TG(18:1/34:4)	1.49 ± 1.16	1.47 ± 0.990
TG(18:1/35:2)	3.14 ± 2.11	3.30 ± 1.74
TG(18:1/35:3)	0.895 ± 0.653	0.946 ± 0.606
TG(18:1/36:0)	1.45 ± 1.13	1.55 ± 0.711
TG(18:1/36:1)	21.5 ± 14.3	24.3 ± 10.7
TG(18:1/36:2)	96.7 ± 64.0	118 ± 52.8
TG(18:1/36:3)	74.9 ± 62.2	78.0 ± 41.0
TG(18:1/36:4)	22.7 ± 28.0	20.8 ± 15.5
TG(18:1/36:5)	2.97 ± 2.25	3.11 ± 2.11
TG(18:1/36:6)	0.351 ± 0.243	0.375 ± 0.285
TG(18:1/38:5)	3.68 ± 2.45	3.55 ± 1.94
TG(18:1/38:6)	2.61 ± 1.70	2.47 ± 1.40
TG(18:1/38:7)	0.566 ± 0.415	0.527 ± 0.279
TG(18:2/28:0)	0.522 ± 0.666	0.430 ± 0.978
TG(18:2/30:0)	2.38 ± 2.67	1.84 ± 2.92
TG(18:2/30:1)	2.03 ± 2.61	1.66 ± 3.01
TG(18:2/32:0)	8.32 ± 8.26	6.18 ± 5.45
TG(18:2/32:1)	13.3 ± 11.2	11.6 ± 11.4
TG(18:2/32:2)	3.81 ± 3.97	3.53 ± 4.85
TG(18:2/33:0)	0.463 ± 0.386	0.421 ± 0.420
TG(18:2/33:1)	1.66 ± 1.31	1.54 ± 1.39
TG(18:2/33:2)	0.638 ± 0.628	0.614 ± 0.724
TG(18:2/34:0)	9.21 ± 7.78	8.06 ± 5.22
TG(18:2/34:1)	83.2 ± 65.2	75.4 ± 46.9
TG(18:2/34:2)	48.1 ± 49.2	41.4 ± 34.4

Analyte	V1 mean ± SD (μM)	V2 mean ± SD (μM)
TG(18:2/34:3)	6.71 ± 7.03	6.09 ± 5.19
TG(18:2/34:4)	0.615 ± 0.653	0.553 ± 0.461
TG(18:2/35:1)	1.41 ± 1.05	1.33 ± 1.04
TG(18:2/35:2)	1.24 ± 1.01	1.23 ± 0.964
TG(18:2/35:3)	0.399 ± 0.408	0.343 ± 0.341
TG(18:2/36:0)	0.864 ± 0.729	0.845 ± 0.535
TG(18:2/36:1)	9.85 ± 7.93	9.81 ± 5.58
TG(18:2/36:2)	39.6 ± 35.0	40.1 ± 22.8
TG(18:2/36:3)	29.9 ± 45.7	25.0 ± 24.9
TG(18:2/36:4)	11.7 ± 24.9	8.42 ± 11.3
TG(18:2/36:5)	1.14 ± 1.17	1.17 ± 1.15
TG(18:2/38:4)	1.30 ± 1.37	1.14 ± 0.923
TG(18:2/38:5)	1.54 ± 1.45	1.34 ± 1.04
TG(18:2/38:6)	1.18 ± 1.16	0.994 ± 0.697
TG(18:3/30:0)	0.248 ± 0.245	0.177 ± 0.271
TG(18:3/32:0)	0.830 ± 0.660	0.594 ± 0.544
TG(18:3/32:1)	1.19 ± 0.786	1.06 ± 1.06
TG(18:3/34:0)	0.793 ± 0.526	0.715 ± 0.517
TG(18:3/34:1)	7.33 ± 5.16	6.58 ± 4.41
TG(18:3/34:2)	3.93 ± 3.11	3.54 ± 2.86
TG(18:3/34:3)	0.576 ± 0.423	0.581 ± 0.484
TG(18:3/36:1)	0.944 ± 0.622	1.01 ± 0.601
TG(18:3/36:2)	4.44 ± 2.90	4.89 ± 2.62
TG(18:3/36:3)	2.97 ± 2.43	3.10 ± 2.41
TG(18:3/36:4)	0.856 ± 0.765	0.896 ± 0.805
TG(18:3/38:5)	0.201 ± 0.145	0.178 ± 0.133
TG(18:3/38:6)	0.141 ± 0.0859	0.118 ± 0.0985
TG(20:0/32:3)	0.198 ± 0.147	0.198 ± 0.121
TG(20:0/32:4)	0.162 ± 0.151	0.140 ± 0.103

Analyte	V1 mean ± SD (μM)	V2 mean ± SD (μM)
TG(20:1/32:1)	0.239 ± 0.202	0.208 ± 0.166
TG(20:1/34:1)	1.04 ± 0.820	1.02 ± 0.667
TG(20:1/34:2)	0.674 ± 0.577	0.644 ± 0.477
TG(20:1/34:3)	0.150 ± 0.125	0.160 ± 0.0949
TG(20:2/32:1)	0.326 ± 0.263	0.281 ± 0.252
TG(20:2/34:1)	1.24 ± 1.03	1.16 ± 0.820
TG(20:2/34:2)	0.806 ± 0.675	0.725 ± 0.598
TG(20:2/34:3)	0.161 ± 0.128	0.157 ± 0.147
TG(20:3/32:0)	0.345 ± 0.297	0.258 ± 0.314
TG(20:3/32:1)	0.560 ± 0.463	0.485 ± 0.505
TG(20:3/34:0)	0.322 ± 0.245	0.283 ± 0.238
TG(20:3/34:1)	2.93 ± 2.23	2.66 ± 2.22
TG(20:3/34:2)	1.84 ± 1.61	1.62 ± 1.45
TG(20:3/34:3)	0.331 ± 0.280	0.270 ± 0.239
TG(20:3/36:3)	1.28 ± 1.10	1.13 ± 0.812
TG(20:3/36:4)	0.423 ± 0.425	0.372 ± 0.322
TG(20:4/30:0)	0.250 ± 0.268	0.173 ± 0.331
TG(20:4/32:0)	0.881 ± 0.921	0.573 ± 0.692
TG(20:4/32:1)	1.02 ± 0.834	0.810 ± 1.03
TG(20:4/32:2)	0.329 ± 0.304	0.275 ± 0.339
TG(20:4/34:0)	0.847 ± 0.625	0.719 ± 0.568
TG(20:4/34:1)	6.50 ± 4.81	5.63 ± 4.24
TG(20:4/34:2)	4.11 ± 3.79	3.25 ± 3.05
TG(20:4/34:3)	0.633 ± 0.496	0.477 ± 0.395
TG(20:4/36:2)	5.16 ± 3.32	5.08 ± 2.97
TG(20:4/36:3)	3.14 ± 2.45	2.72 ± 2.09
TG(20:4/36:4)	1.06 ± 1.08	0.830 ± 0.772
TG(20:5/34:0)	0.216 ± 0.162	0.187 ± 0.126
TG(20:5/34:1)	1.70 ± 1.26	1.56 ± 1.13

Analyte	V1 mean $\pm$ SD (μM)	V2 mean $\pm$ SD (μM)
TG(20:5/34:2)	1.13 $\pm$ 0.977	0.991 $\pm$ 0.857
TG(20:5/36:2)	1.41 $\pm$ 0.985	1.43 $\pm$ 0.909
TG(20:5/36:3)	1.14 $\pm$ 1.20	0.927 $\pm$ 0.712
TG(22:4/34:2)	0.415 $\pm$ 0.344	0.341 $\pm$ 0.280
TG(22:5/32:0)	0.273 $\pm$ 0.228	0.219 $\pm$ 0.160
TG(22:5/32:1)	0.384 $\pm$ 0.257	0.316 $\pm$ 0.232
TG(22:5/34:1)	2.32 $\pm$ 1.60	2.08 $\pm$ 1.09
TG(22:5/34:2)	1.39 $\pm$ 1.02	1.22 $\pm$ 0.690
TG(22:6/32:0)	0.809 $\pm$ 0.728	0.617 $\pm$ 0.492
TG(22:6/32:1)	1.08 $\pm$ 0.844	0.964 $\pm$ 0.783
TG(22:6/34:1)	6.95 $\pm$ 5.12	6.77 $\pm$ 5.00
TG(22:6/34:2)	4.44 $\pm$ 3.44	4.13 $\pm$ 3.41
Threonic acid	8.78 $\pm$ 3.25	8.17 $\pm$ 2.03
Threonine	160 $\pm$ 77.9	154 $\pm$ 62.5
trans-4-Hydroxyproline	7.38 $\pm$ 3.72	6.35 $\pm$ 3.28
Trimethylamine	20.9 $\pm$ 9.87	20.1 $\pm$ 5.77
Trimethylamine N-Oxide	11.0 $\pm$ 7.51	12.5 $\pm$ 15.2
Tryptophan	60.2 $\pm$ 24.6	55.9 $\pm$ 16.4
Tyrosine	145 $\pm$ 45.6	137 $\pm$ 31.4
Urea	9460 $\pm$ 2450	9080 $\pm$ 3320
Uric acid	241 $\pm$ 75.1	239 $\pm$ 67.2
Uridine	4.05 $\pm$ 1.29	4.56 $\pm$ 1.46
Valine	285 $\pm$ 91.3	261 $\pm$ 63.4
Xanthine	1.02 $\pm$ 2.19	0.975 $\pm$ 1.81
